# Relationship between the Hemoglobin-to-Red Cell Distribution Width Ratio and All-Cause Mortality in Septic Patients with Atrial Fibrillation: Based on Propensity Score Matching Method

**DOI:** 10.3390/jcdd9110400

**Published:** 2022-11-18

**Authors:** Junhong Wang, Zirong Chen, Hongkuan Yang, Hua Li, Rudong Chen, Jiasheng Yu

**Affiliations:** Department of Neurosurgery, Tongji Hospital, Tongji Medical College, Huazhong University of Science and Technology, Wuhan 430074, China

**Keywords:** atrial fibrillation, sepsis, hemoglobin-to-red blood cell distribution width ratio, all-cause mortality, MIMIC-IV database

## Abstract

(1) Objective: To reveal the correlation between the hemoglobin-to-red cell distribution width ratio (HRR) and all-cause mortality (ACM) among the septic patients with atrial fibrillation. (2) Methods: Specific clinical information was collected from the Medical Information Mart for Intensive IV (MIMIC-IV) database. The optimal cut-off value of HRR was calculated through ROC curve analysis conducted by using the maximum Youden index for the prediction of survival status. In addition, univariable and multivariable Cox regressive analyses were carried out to assess the prognostic significance of HRR and the Kaplan-Meier (K-M) analysis was conducted to draw the survival curves. Then, the 1:1 propensity score matching (PSM) method was adopted to improve the reliability of research result while balancing the unintended influence of underlying confounders. (3) Results: There were 9228 patients participating in this retrospective cohort study. The optimal cut-off value of the HRR was determined as 5.877 for in-hospital mortality. The PSM was performed to identify 2931 pairs of score-matched patients, with balanced differences exhibited by nearly all variables. According to the K-M analysis, those patients with a lower HRR than 5.877 showed a significantly higher level of in-hospital mortality, 28-day mortality, and 90-day mortality, compared to the patients with HRR ≥ 5.877 (*p* < 0.001). After the adjustment of possible confounders, those patients whose HRR was below 5.877 had a significantly higher level of in-hospital mortality than the patients with HRR ≥ 5.877, as revealed by the multivariable Cox regression analysis (HR = 1.142, 95%CI: 1.210–1.648, *p* < 0.001). Similarly, the ACM remained substantially higher in those patients with a lower HRR than in the patients with higher HRR after PSM. (4) Conclusion: A lower HRR (<5.877) was evidently associated with an increased risk of ACM, which made it applicable as a prognostic predictor of clinical outcomes for those septic patients with atrial fibrillation.

## 1. Introduction

Sepsis, a life-threatening, dysfunctional infection response, is one of the leading causes of severe illness, affecting more than 1.5 million Americans and costing more than USD 20 billion annually [1]. The characteristics of sepsis are the release of proinflammatory cytokines, autonomic dysfunction, and possibly organ dysfunction [2]. With the decrease of in-hospital mortality in sepsis, the number of patients facing post-sepsis rehabilitation complications has increased [3]. Especially, the increased risk of adverse cardiovascular events in patients with sepsis, including atrial fibrillation, myocardial infarction, ischemic stroke, and heart failure [4,5]. Atrial fibrillation is a common and potentially fatal complication of sepsis [1,6,7,8], which increases the risk of serious adverse events in long-time observation [8].

For septic patients, the complete blood count is part of routine examination, in which hemoglobin (Hb) is treated as important and related to malnutrition and suppressed immune response. In addition, the low level of pre-treatment Hb concentration can be used to predict the adverse prognosis of patients with various cancers, including lung, gastric and breast cancers, esophageal squamous cell carcinoma, cervical cancer, and nasopharyngeal carcinoma [9,10,11,12,13]. As another major component of complete blood count, red cell distribution width (RDW) can be used as an index to measure the heterogeneity of those red blood cells circulating in peripheral bloodstream. Recently, RDW has been demonstrated as associated with the poor prognosis of cardiovascular, malignant diseases, and liver disease [14,15]. Recently, the Hb-to-RDW ratio (HRR) has been identified as a novel prognostic marker. In addition, there are plenty of studies suggesting a link between the low level of HRR and the poor outcomes of treatment of several malignant diseases [16,17,18,19,20,21].

Therefore, the present study is aimed at exploring the prognostic significance of the HRR in septic patients with atrial fibrillation and providing a simple and convenient indicator for high-risk patients.

## 2. Materials and Methods

### 2.1. Data Sources

We extracted the data from MIMIC-IV [22], a free and publicly available database. It is the improved version of MIMIC-III. We were allowed to extract data after we completed the training courses regulated by the National Institutes of Health (NIH) as well as the Protecting Human Research Participants examination. One author, Junhong Wang, was approved to utilize the database. In addition, the study has obtained the approval of the Institutional Review Boards of Beth Israel Deaconess Medical Center and the Massachusetts Institute of Technology (Cambridge, MA, USA), and further ethical approval was not needed. The findings of the study were reported following the Strengthening the Reporting of Observational Studies in Epidemiology guidelines [23].

### 2.2. Study Population

**Figure 1:**
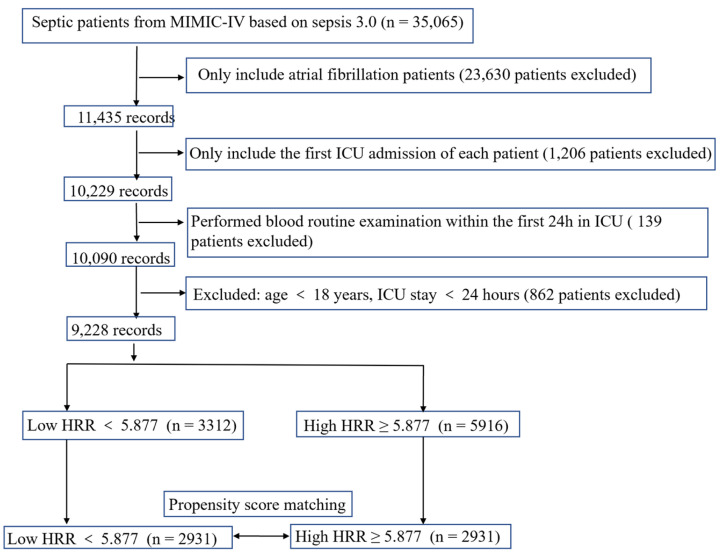
The flow chart of the study.

The total number of patients in the MIMIC-IV included 257,366 individuals from 2008 to 2019. Among them, 11,435 septic patients with atrial fibrillation were selected based on the record of ICD-9. For patients who were admitted to the ICU multiple times, only the first ICU admission data were included. Patients without HRR data within 24 h after admission were not included. ICU patients with length of stay was less than 24 h also excluded to avoid potential extremum value influence. Patients >18 years were enrolled in this study. Thus, only 9228 patients were included in this study. The workflow is shown in Figure 1.

### 2.3. Data Extraction

We extracted variables of patients on the first day that they were admitted to ICU from the MIMIC-IV database: (1) demographics: sex, age and ethnicity; (2) vital signs: systolic blood pressure (SBP), respiratory rate (RR), heart rate (HR), diastolic blood pressure (DBP), mean blood pressure (MBP), temperature and percutaneous oxygen saturation (SpO2); (3) comorbidities: myocardial infarct, congestive heart failure (CHF), chronic pulmonary disease, peripheral vascular disease etc.; (4) laboratory results: Hb, RDW, platelets, white blood cell (WBC) count, albumin, anion gap, bicarbonate, blood urea nitrogen (BUN), calcium, chloride, creatinine, sodium, lymphocytes, monocytes, neutrophils, international normalized ratio (INR), prothrombin time (PT), activated partial thromboplastin time (APTT), creatine kinase-MB isoenzyme (CK-MB), glucose, alanine aminotransferase (ALT), aspartate aminotransferase (AST) were measured during the first 24 h of being admitted to intensive care unit (ICU). If a variable was measured more than once in the first 24 h, we used the mean value; (5) the sequential organ failure assessment (SOFA) score, Acute Physiology Score III (APS III), Oxford Acute Severity of Illness Score (OASIS), and Glasgow Coma Scale (GCS) were considered to measure the admission severity; and (7) HRR calculation was based on the formula of HRR = Hb (g/L)/RDW (%) [24,25,26].

### 2.4. Endpoints

The primary outcome was in-hospital mortality. Secondary outcomes were 28-day mortality, 60-day mortality, 90-day mortality, ICU stay length, and hospital stay length.

### 2.5. Statistical Analysis

The continuous variates were displayed as average ± standard deviation (SD) or mid-value (interquartile range). Student’s t-test or the Mann-Whitney U-test were used according to the normality of the distribution. Categorical variates were displayed as case quantity (%), and the chi-square test (or Fisher’s exact approach) was utilized for analyses.

The optimal cut-off value of HRR was calculated through ROC curve analysis conducted by using the maximum Youden index for the prediction of survival status. Youden index = sensitivity + specificity −1. HRR was divided into low HRR (<5.877) group and high HRR (≥5.877) group.

In addition, univariable and multivariable Cox regressive analyses were carried out to assess the prognostic significance of HRR. The screening criteria of confounders: (1) the factor affected the research variates (with impact over 10%); (2) the outcome variates might be obviously impacted by some factors based on previous experiences; and (3) the variates with *p* value less than 0.05 in univariable analysis. In the multivariable, we performed some different statistical models to verify the stability of the results.

The crude model did not adjust variables. Model I made adjustment on variables of age, gender, and ethnicity, while Model II made adjustment on 10 other variables, including hematocrit, BUN, chloride, creatinine, SBP, DBP, MBP, Spo2, APS III, and hypertension. Model II made further adjustment on the other 25 variables in Model III, including myocardial infarct, peptic ulcer disease, paraplegia, congestive heart failure, metastatic solid tumor, cerebrovascular disease, renal disease, malignant cancer, platelets, sodium, anion gap, calcium, INR, PT, APTT, HR; RR, the use of warfarin, dopamine, vasopressin, antibiotic, and SOFA score, GCS, OASIS, and length of ICU stay.

Given the difficulty in achieving complete stochasticity for the screening of suffers, the propensity score matching (PSM) approach was adopted to balance the influence of selection bias and that of underlying confounders. The PSM analysis was conducted through the logistic regression model developed using age, sex, ethnicity, SBP, DBP, MBP, SpO2, hypertension, etc. The PSM degree was assessed against a standardized mean difference (SMD), and a lower threshold than 0.1 was treated as acceptable. For the pairs of patients with low HRR (<5.877) and high HRR (≥5.877), 1:1 matching was performed with a caliper of 0.02. Finally, 5862 propensity score-matched patients and 2931 pairs of score-matched patients were identified.

The subgroup analysis was conducted to reveal how HRR affected the in-hospital mortality from various perspectives including age (<65 and ≥65 years old), gender, comorbidities, SOFA (<3 and ≥3), APS III (<54 and ≥54), myocardial infarct, peptic ulcer disease, renal disease, paraplegia, congestive heart failure, chronic pulmonary disease, dementia, peripheral vascular disease, cerebrovascular disease, rheumatic disease, and peptic ulcer disease. We conducted the subgroup analyses under the assistance of a Cox regression model.

The statistic program packages R 3.3.2 (http://www.R-project.org, The R Foundation), Free Statistics software version 1.4 (Beijing, China) and SPSS 21.0 (IBM SPSS, Armonk, NY, USA) assisted in completing all analyses. The study carried out a two-tailed test and *p* < 0.05 reported statistical significance.

## 3. Results

### 3.1. Data Sources

We selected patients that met the preset standards (see Figure 1 for a flow chart).

### 3.2. Clinical Characteristics of Study Subjects

Table 1 listed the demographic data, vital signs, comorbidities, treatment, laboratory events, scores, as well as outcomes between survivor and non-survivor groups. Overall, the median age was 76.0 years old, and approximately 40.3% of these were women. The non-survivor group presented obviously lower HRR (median: 6.3 vs. 6.6, *p* < 0.001). Compared with the survivor group, the non-survivor group was older (78.0 vs. 75.0 years old, *p* < 0.001), and presented a higher comorbidity incidence, such as myocardial infarct, CHF, paraplegia, renal disease, malignant cancer, hypertension, as well as higher OASIS, APS III scores and lower GCS scores (all *p* values < 0.05) (Table 1). In terms of dynamic characteristics, the levels of platelets, anion gap, BUN, creatinine, INR, PT, and APTT in survivors decreased significantly (Table 1).

### 3.3. The Prognostic Significance of HRR before PSM

The ROC curve of HRR was plotted, and the AUC was 0.548 (95%CI, 0.533–0.564) (Figure S1). The optimal cut-off value of HRR was calculated through ROC curve analysis conducted by using the maximum Youden index for the prediction of survival status. Youden index = sensitivity + specificity − 1. The corresponding optimal cut-off value was 5.877, the evaluation sensitivity was 65.6% and the specificity was 42.9% (Table S1). Based on the cut-off value, 9228 patients were divided into low HRR (HRR < 5.877, *n* = 3312) group and high HRR (HRR ≥ 5.877, *n* = 5916) group. The demographics, coexisting diseases, vital signs, scoring, laboratory events, etc. are presented in Table 2. Compared with patients with HRR ≥ 5.877, patients with HRR < 5.877 group were at higher risk of 28-day mortality (21.7 vs. 15.9%, *p* < 0.001), 60-day mortality (24.0 vs. 17.8%, *p* < 0.001), 90-day mortality (24.8 vs. 18.4%, *p* < 0.001), in-hospital mortality (22.0 vs. 16.5%, *p* < 0.001), prolonged ICU stay (4.0 vs. 3.0 days, *p* = 0.008), and the comorbidity incidence was higher, such as congestive heart failure (*p* < 0.05) (Table 2).

### 3.4. Association between HRR and All-Cause Mortality in Septic Patients with Atrial Fibrillation before PSM

A univariable Cox regression analysis was used to select the variables of prognostic value for in-hospital mortality. The multivariable Cox regression analysis adjusted the selected variables, such as age (*p* < 0.001), gender (*p* < 0.001), myocardial infarct (*p* = 0.001), CHF (*p* < 0.001), cerebrovascular disease (*p* < 0.001), peptic ulcer disease (*p* = 0.001), malignant cancer (*p* < 0.001), metastatic solid tumor (*p* < 0.001), hypertension (*p* < 0.001), and other variables (*p* < 0.05). Table S2 and Table 3 list the univariable and multivariable analysis results.

Table 3 shows an unadjusted and a multivariable-adjusted correlation between HRR and in-hospital mortality. In the crude model, HRR was negatively correlated with in-hospital mortality (HR = 0.919, 95% CI: 0.892–0.946, *p* < 0.001). Age, sex, and ethnicity were adjusted in Model I, while Model II further adjusted the other 10 variables, including hematocrit, BUN, chloride, creatinine, SBP, DBP, MBP, Spo2, APS III, and hypertension.

**Figure 2:**
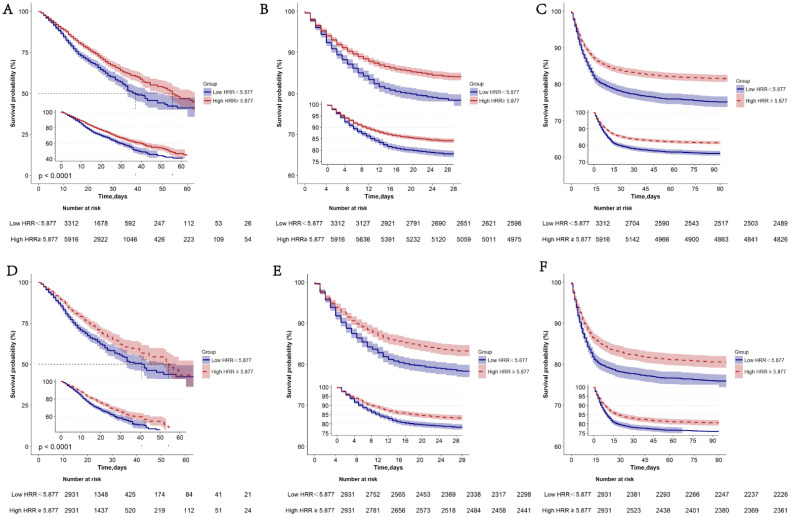
Kaplan–Meier survival curves of in-hospital mortality, 28-day mortality, and 90-day mortality classified into two groups according to HRR before (**A**–**C**) and after PSM (**D**–**F**). PSM, propensity score matching.

Model III further adjusted the other 25 variables. As a continuous variable, HRR was negatively related to the in-hospital mortality (Model I: HR = 0.915, 95% CI: 0.888–0.942, *p* < 0.001; Model II: HR = 0.834, 95% CI: 0.795–0.875, *p* < 0.001; Model III: HR = 0.873, 95% CI: 0.829–0.920, *p* < 0.001). Moreover, as a classification variable, in-hospital mortality increased remarkably for patients in HRR < 5.877 group compared with HRR ≥ 5.877 group (Model I: HR = 1.432, 95% CI: 1.286–1.596, *p* < 0.001; Model II: HR = 1.561, 95% CI: 1.352–1.803, *p* < 0.001; Model III: HR = 1.412, 95% CI: 1.210–1.648, *p* < 0.001). Figure 2 displayed the K-M curves of two groups. HRR < 5.877 group exhibited remarkably higher in-hospital mortality (Figure 2A), 28-day mortality (Figure 2B), and 90-day mortality (Figure 2C) compared with HRR ≥ 5.877 group (*p* < 0.001).

### 3.5. Subgroup Analysis

**Figure 3:**
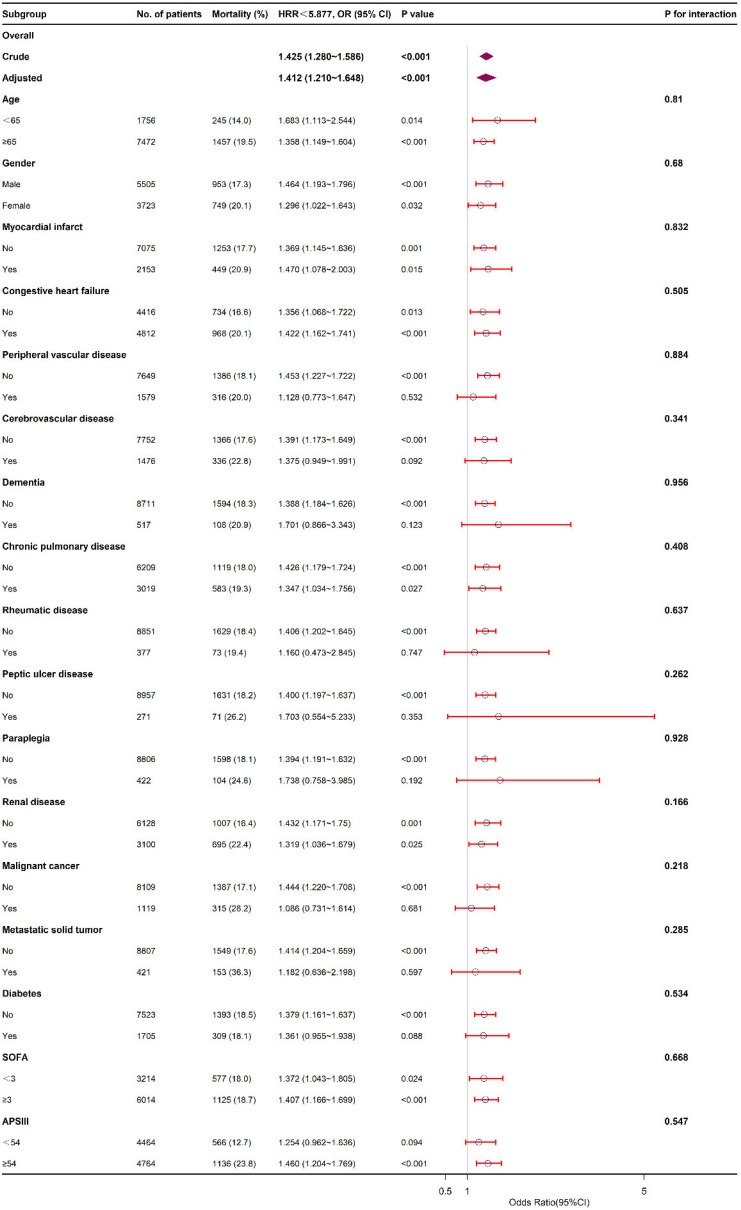
The relationship between HRR and in-hospital mortality in subgroup analysis. 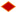 and 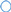 represent the HR value. Red bands present the 95% confidence interval.

The subgroup analysis was conducted to reveal how HRR affected the in-hospital mortality from various perspectives including age (<65 and ≥65 years old), gender, comorbidities, SOFA (<3 and ≥3), APS III (<54 and ≥54) (Figure 3). Accordingly, HRR < 5.877 group presented higher in-hospital mortality rate compared with HRR ≥ 5.877 group in all subgroups. The study paid attention to analyzing the interactions between HRR and all subgroup factors, finding no obvious interaction (*p* > 0.05).

### 3.6. Prognostic Value of HRR after PSM

**Figure 4:**
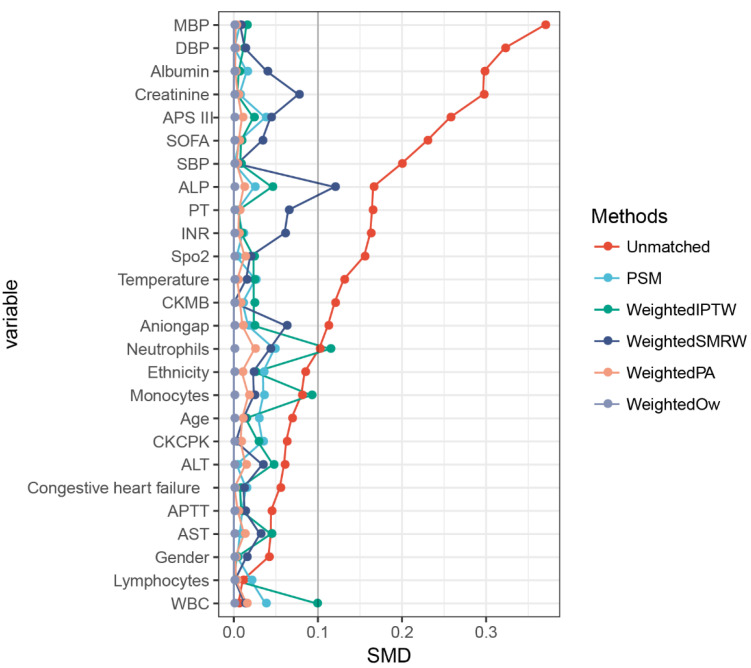
The results of matching. A standardized mean difference (SMD) was used to examine the degree of PSM. A threshold of less than 0.1 was considered acceptable. PSM, propensity score matching [27]; weighted IPTW, weighted the inverse probability of treatment [28]; weighted SMRW, weighted the standardized mortality ratio weighting [28]; weighted PA, weighted pairwise algorithmic [29]; weighted OW, weighted overlap weight [30].

Considering the imbalanced baseline features of the two groups, a 1:1 ratio PSM was performed to balance the latent confounders, with 2931 pairs of score-matched sufferers obtained. The difference between the two groups was balanced for nearly all variables, with a satisfactory matching performance achieved (Figure 4).

The clinical characteristics of septic patients with atrial fibrillation after PSM are shown in Table 4. After PSM, the two groups still presented obvious difference in terms of the 28-day mortality (21.7 vs. 16.7%, *p* < 0.001), 60-day mortality (23.3 vs. 18.9%, *p* < 0.001), 90-day mortality (24.1 vs. 19.4%, *p* < 0.001), and in-hospital mortality (21.6 vs. 17.2%, *p* < 0.001) (Table 4).

### 3.7. Association between HRR and All-Cause Mortality in Septic Patients with Atrial Fibrillation after PSM

In the unadjusted model, as a continuous variable, the HR of HRR level for in-hospital mortality was 0.931 (95% CI: 0.895–0.968). As a categorical variable, HRR < 5.877 group presented remarkably higher in-hospital mortality compared with HRR ≥ 5.877 group (95% CI: 1.321, 1.159–1.504). According to multivariable Cox regression analysis for patients after PSM, the in-hospital mortality in HRR < 5.877 group remained obviously higher relative to HRR ≥ 5.877 group (Model I: HR = 1.320, 95% CI: 1.158–1.504, *p* < 0.001; Model II: HR = 1.366, 95% CI: 1.149–1.623, *p* < 0.001; Model III: HR = 1.374, 95% CI: 1.144–1.649, *p* < 0.001) (Table 3). Additionally, Figure 2 displayed the Kaplan-Meier survival curves. After PSM, HRR < 5.877 group exhibited an obviously higher in-hospital mortality (Figure 2D), 28-day mortality (Figure 2E), and 90-day mortality (Figure 2F) compared with HRR ≥ 5.877 group (*p* < 0.001).

## 4. Discussion

The study included 3312 (HRR < 5.877) and 5916 (HRR ≥ 5.877) septic patients with atrial fibrillation from the MIMIC-IV database. The univariable, multivariable regression analysis and PSM were performed to relieve the interference of possible confounding factors on the in-hospital mortality. This large retrospective cohort study suggested that a lower level of HRR was more likely to have a higher risk of all-cause mortality. This is the first study to investigate the influence of the HRR on the prognosis of septic patients with atrial fibrillation.

RDW was a reliable indicator of the size distribution and anisocytosis of red blood cells. In recent years, studies have shown that RDW was an effective predictor of prognosis in many pathological conditions, such as cardiovascular diseases [31,32], sepsis [33,34,35], ischemic stroke [36], influenza [37], etc. Hb was an important component and related to malnutrition and decreased immune response. Julián N Acosta et al. conducted a study of 4172 non-traumatic intracerebral hemorrhage patients and found that higher admission Hb levels were related to a lower risk of poor outcomes in intracerebral hemorrhage [38]. I. Gauthier et al. [39] explored the relationship between hemoglobin levels and the outcomes of adjuvant chemotherapy in resected non-small-cell lung cancer and found that lower Hb (<120 g/L) was predicted for in-hospital stay and worse poor quality of life.

The prognosis value of Hb or RDW has been reported separately among patients with cardiovascular disease. However, the research on HRR was limited. Xiu et al. conducted a retrospective cohort study of 6046 coronary atherosclerotic heart disease patients with percutaneous coronary intervention and found that low levels of the HRR (HRR < 10.25) increased long-term all-cause mortality and cardiac mortality by 1.470 times and 1.479 times, respectively [40]. The study by Eldad Rahamim et al. demonstrated that HRR was a significant independent predictor for cardiovascular hospitalizations in heart failure patients and decreasing quantiles of the HRR were related to reduced survival rates [26]. Qu et al. [25] analyzed 233 elderly patients with coronary heart disease and found that HRR was a stronger predictor of frailty than Hb or RDW and lower HRR (HRR < 9.76) was an independent risk factor for frailty in elderly patients with coronary heart disease. In addition, a series of studies have demonstrated that a low level of HRR was linked to poor outcomes in several malignant diseases [41,42].

However, as far as we know, the prognosis effect of HRR in septic patients with atrial fibrillation has remained unclear. Here, how HRR affected in-hospital mortality in septic patients with AF was investigated. The retrospective cohort study involved 9228 patients, and the cut-off value of HRR was considered to divide them into two groups. Compared with patients with HRR ≥ 5.877 group, the HRR < 5.877 group exhibited higher risk of 28-day mortality (21.7 vs. 15.9%, *p* < 0.001), 60-day mortality (24.0 vs. 17.8%, *p* < 0.001), 90-day mortality (24.8 vs. 18.4%, *p* < 0.001), in-hospital mortality (22.0 vs. 16.5%, *p* < 0.001), prolonged ICU stay (4.0 vs. 3.0 days, *p* = 0.008), with higher incidence of comorbidities.

Several hypothesized mechanisms have been proposed to explain the reason why lower HRR leads to adverse outcomes in septic patients, with atrial fibrillation. Firstly, higher RDW in the normal range may imply RBC disruption or, more commonly, ineffective erythropoiesis [43]. Moreover, higher RDW also reflects an underlying inflammatory state and is related to adverse outcomes [44,45]. Zsolt Förhécz et al. performed a retrospective cohort study involving 195 patients suffering chronic heart failure and found the correlation between RDW and inflammatory markers like C-reactive protein, interleukin-6, soluble tumor necrosis factor (TNF) receptor I and II [46]. Secondly, the oxygen carrying capacity was mainly determined by the Hb level. The reduction of Hb value indicated that the oxygen supply to the myocardium downstream of coronary arteries was significantly reduced, and the oxygen supply of tissue was limited, which may cause atrial fibrillation. Moreover, inflammation reaction was very important in sepsis, and low hemoglobin level was an important indicator of the underlying inflammatory process.

The advantages of this research are as follows. On the one hand, statistical reliability was improved by a large sample size. On the other hand, the selection bias was reduced by the lower missing HRR. Despite these findings, there remain some limitations on this research. Firstly, this is a single-center study and multicenter studies are required to verify the conclusions. Secondly, the data on HRR was collected during the first 24 h in ICU, and it was difficult to analyze the dynamic changes of HRR. Thirdly, the optimal cut-off value of HRR was calculated through ROC curve analysis, with the maximum Youden index adopted to predict the survival status. HRR was considered applicable to divide patients into low HRR (<5.877) group and high HRR (≥5.877) group. However, the AUC of HRR was 0.548 (95% CI, 0.533–0.564), which was lower than expected. Therefore, it is necessary to verify the results through further studies. Lastly, this is a retrospective observational study, with attempt made to adjust the factors by PSM and multivariable analysis. In spite of this, there remained some confounding factors left unmeasured, including lactic acid, C-reactive protein, inotropic equivalent, shock, and other possible factors.

## 5. Conclusions

In conclusion, this was the first time the prognostic significance of HRR in septic patients with atrial fibrillation has been investigated. A lower HRR (<5.877) was associated with a higher risk of all-cause mortality and can serve as a prognostic predictor of clinical outcomes in septic patients with atrial fibrillation.

## Figures and Tables

**Table 1:** The baseline clinical characteristics of septic patients with atrial fibrillation.

Variables	Total (*n* = 9228)	Survival (*n* = 7526)	Non-Survival (*n* = 1702)	*p*-Value
Demographic				
Female, *n* (%)	3723 (40.3)	2974 (39.5)	749 (44)	<0.001
Age, years	76.0 (67.0, 84.0)	75.0 (67.0, 83.0)	78.0 (70.0, 85.0)	<0.001
Ethnicity, *n* (%)				<0.001
Asian	244 (2.6)	208 (2.8)	36 (2.1)	
White	6793 (73.6)	5613 (74.6)	1180 (69.3)	
Black	658 (7.1)	542 (7.2)	116 (6.8)	
Other	1533 (16.6)	1163 (15.5)	370 (21.7)	
Vital signs				
HR, beats/minute	85.0 (75.0, 97.0)	84.0 (75.0, 96.0)	88.0 (75.0, 102.0)	<0.001
SBP, mmHg	111.0 (104.0, 120.0)	111.0 (104.0, 119.0)	113.0 (103.0, 133.0)	<0.001
DBP, mmHg	59.0 (53.0, 65.0)	58.0 (53.0, 64.0)	61.0 (55.0, 69.0)	<0.001
MBP, mmHg	74.0 (69.0, 80.0)	74.0 (69.0, 79.0)	75.5 (69.0, 84.0)	<0.001
RR, times/minute	19.0 (17.0, 22.0)	19.0 (17.0, 22.0)	20.0 (18.0, 23.0)	<0.001
Temperature, °C	36.8 (36.6, 37.0)	36.8 (36.6, 37.0)	36.8 (36.5, 37.1)	0.149
SpO2, %	97.0 (96.0, 98.0)	97.0 (96.0, 98.0)	97.0 (95.0, 98.0)	<0.001
Comorbidities, *n* (%)				
Myocardial infarct	2153 (23.3)	1704 (22.6)	449 (26.4)	0.001
Congestive heart failure	4812 (52.1)	3844 (51.1)	968 (56.9)	<0.001
Peripheral vascular disease	1579 (17.1)	1263 (16.8)	316 (18.6)	0.084
Cerebrovascular disease	1476 (16.0)	1140 (15.1)	336 (19.7)	<0.001
Chronic pulmonary disease	3019 (32.7)	2436 (32.4)	583 (34.3)	0.142
Rheumatic disease	377 (4.1)	304 (4)	73 (4.3)	0.688
Peptic ulcer disease	271 (2.9)	200 (2.7)	71 (4.2)	0.001
Paraplegia	422 (4.6)	318 (4.2)	104 (6.1)	<0.001
Renal disease	3100 (33.6)	2405 (32)	695 (40.8)	<0.001
Malignant cancer	1119 (12.1)	804 (10.7)	315 (18.5)	<0.001
Diabetes	1705 (18.5)	1396 (18.5)	309 (18.2)	0.731
Hypertension	543 (5.9)	173 (2.3)	370 (21.7)	<0.001
Therapies, *n* (%)				
Warfarin	737 (8.0)	656 (8.7)	81 (4.8)	<0.001
Amiodarone	1739 (18.8)	1432 (19)	307 (18)	0.364
Dopamine	514 (5.6)	388 (5.2)	126 (7.4)	<0.001
Epinephrine	937 (10.2)	777 (10.3)	160 (9.4)	0.274
Vasopressin	1256 (13.6)	907 (12.1)	349 (20.5)	<0.001
Antibiotic	1801 (19.5)	1509 (20.1)	292 (17.2)	0.007
Ventilation	4823 (52.3)	3906 (51.9)	917 (53.9)	0.148
Laboratory events				
Hemoglobin, g/dL	10.0 (8.6, 11.5)	9.9 (8.6, 11.5)	10.0 (8.6, 11.5)	0.890
RDW, %	15.0 (13.9, 16.8)	14.9 (13.8, 16.6)	15.7 (14.4, 17.6)	<0.001
HRR	6.5 (5.3, 7.9)	6.6 (5.4, 8.0)	6.3 (5.0, 7.7)	<0.001
Platelets, 109/L	198.0 (145.0, 268.0)	195.0 (145.0, 263.0)	206.0 (148.0, 288.0)	<0.001
WBC, 109/L	13.9 (10.0, 18.8)	14.1 (10.1, 18.9)	13.4 (9.6, 18.4)	0.007
Albumin, g/dL	3.2 (3.2, 3.2)	3.2 (3.2, 3.2)	3.2 (3.1, 3.2)	0.706
Anion gap, mmol/L	16.0 (13.0, 19.0)	16.0 (13.0, 19.0)	17.0 (15.0, 21.0)	<0.001
Bicarbonate, mmol/L	24.0 (22.0, 27.0)	24.0 (22.0, 27.0)	24.0 (21.0, 28.0)	0.177
Bun, mg/dL	28.0 (18.0, 46.0)	27.0 (18.0, 44.0)	33.5 (21.0, 54.0)	<0.001
Calcium, mg/dL	8.6 (8.1, 8.9)	8.6 (8.1, 8.9)	8.6 (8.2, 9.1)	<0.001
Chloride, mmol/L	106.0 (101.0, 110.0)	106.0 (102.0, 110.0)	104.0 (100.0, 109.0)	<0.001
Creatinine, mg/dL	1.3 (0.9, 2.1)	1.2 (0.9, 2.0)	1.5 (1.0, 2.5)	<0.001
Sodium, mmol/L	140.0 (137.0, 142.0)	140.0 (137.0, 142.0)	140.0 (137.0, 143.0)	0.049
Lymphocytes, 10^9^/L	88.6 (2.2, 88.6)	88.6 (2.4, 88.6)	88.6 (1.5, 88.6)	<0.001
Monocytes, 10^9^/L	34.9 (1.3, 35.3)	34.9 (1.4, 35.0)	34.9 (1.1, 36.5)	0.096
Neutrophils, 10^9^/L	642.2 (17.6, 753.6)	642.2 (19.0, 750.6)	642.2 (13.8, 774.5)	0.012
INR	1.5 (1.3, 2.0)	1.5 (1.3, 2.0)	1.6 (1.3, 2.3)	<0.001
PT, s	16.5 (14.1, 21.8)	16.3 (14.1, 21.8)	17.5 (14.0, 25.1)	<0.001
APTT, s	36.4 (30.4, 48.0)	36.1 (30.4, 48.0)	37.7 (30.5, 51.5)	<0.001
ALT, IU/L	146.0 (23.0, 146.0)	146.0 (24.0, 146.0)	52.0 (20.0, 146.0)	<0.001
AST, U/L	218.5 (35.0, 255.0)	255.0 (37.0, 255.0)	85.0 (30.0, 255.0)	<0.001
CK-MB, U/L	19.0 (8.0, 19.0)	19.0 (9.0, 19.0)	19.0 (5.0, 19.0)	<0.001
Glucose, mg/dL	642.2 (17.6, 753.6)	642.2 (19.0, 750.6)	642.2 (13.8, 774.5)	0.012
Scores				
APSIII	54.0 (40.0, 73.0)	52.0 (39.0, 70.0)	65.0 (48.0, 85.0)	<0.001
SOFA	3.0 (2.0, 5.0)	3.0 (2.0, 5.0)	3.0 (2.0, 5.0)	0.084
GCS	13.0 (9.0, 15.0)	14.0 (10.0, 15.0)	12.0 (7.0, 14.0)	<0.001
OASIS	36.0 (30.0, 43.0)	35.0 (29.0, 42.0)	39.0 (32.0, 45.0)	<0.001
Outcomes				
Length of ICU stay, days	4.0 (2.0, 7.0)	3.0 (2.0, 6.0)	5.0 (3.0, 10.0)	<0.001
Length of hospital stay, days	9.0 (6.0, 16.0)	10.0 (6.0, 16.0)	9.0 (4.0, 15.0)	<0.001

**Table 2:** The clinical characteristics of septic patients with atrial fibrillation before PSM.

Characteristic	Before PSM			
	All Patients	Low HRR < 5.877	High HRR ≥ 5.877	*p*
N	9228	3312	5916	
Demographic				
Female, *n* (%)	3723 (40.3)	1379 (41.6)	2344 (39.6)	0.061
Age, years	76.0 (67.0, 84.0)	76.0 (68.0, 84.0)	76.0 (67.0, 83.0)	0.005
Ethnicity, *n* (%)				0.002
Asian	244 (2.6)	92 (2.8)	152 (2.6)	
White	6793 (73.6)	2498 (75.4)	4295 (72.6)	
Black	658 (7.1)	237 (7.2)	421 (7.1)	
Other	1533 (16.6)	485 (14.6)	1048 (17.7)	
Vital signs				
HR, beats/minute	85 (75, 97)	85 (75, 97)	84 (75, 97)	0.937
SBP, mmHg	111 (104, 120)	109 (102, 118)	112 (105, 122)	<0.001
DBP, mmHg	59 (53, 65)	57 (52, 63)	60 (54, 66)	<0.001
MBP, mmHg	74 (69, 80)	72 (67, 77)	75 (70, 81)	<0.001
RR, times/minute	19 (17, 22)	19 (17, 22)	19 (17, 22)	0.904
Temperature, °C	36.8 (36.6, 37.0)	36.8 (36.5, 37.0)	36.8 (36.6, 37.1)	<0.001
SpO2, %	97 (96, 98)	97 (96, 99)	97 (96, 98)	<0.001
Comorbidities, *n* (%)				
Myocardial infarct	2153 (23.3)	799 (24.1)	1354 (22.9)	0.186
Congestive heart failure	4812 (52.1)	1785 (53.9)	3027 (51.2)	0.013
Peripheral vascular disease	1579 (17.1)	580 (17.5)	999 (16.9)	0.461
Cerebrovascular disease	1476 (16.0)	534 (16.1)	942 (15.9)	0.824
Dementia	517 (5.6)	174 (5.3)	343 (5.8)	0.297
Chronic pulmonary disease	3019 (32.7)	1094 (33)	1925 (32.5)	0.645
Rheumatic disease	377 (4.1)	135 (4.1)	242 (4.1)	1
Peptic ulcer disease	271 (2.9)	103 (3.1)	168 (2.8)	0.501
Paraplegia	422 (4.6)	133 (4)	289 (4.9)	0.062
Renal disease	3100 (33.6)	1138 (34.4)	1962 (33.2)	0.253
Malignant cancer	1119 (12.1)	387 (11.7)	732 (12.4)	0.348
Metastatic solid tumor	421 (4.6)	145 (4.4)	276 (4.7)	0.56
Diabetes	1705 (18.5)	612 (18.5)	1093 (18.5)	1
Hypertension	543 (5.9)	159 (4.8)	384 (6.5)	0.001
Therapies, *n* (%)				
Warfarin	737 (8.0)	271 (8.2)	466 (7.9)	0.632
Amiodarone	1739 (18.8)	539 (16.3)	1200 (20.3)	<0.001
Dopamine	514 (5.6)	180 (5.4)	334 (5.6)	0.707
Epinephrine	937 (10.2)	343 (10.4)	594 (10)	0.656
Vasopressin	1256 (13.6)	503 (15.2)	753 (12.7)	0.001
Antibiotic	1801 (19.5)	554 (16.7)	1247 (21.1)	<0.001
Ventilation	4823 (52.3)	1706 (51.5)	3117 (52.7)	0.287
Laboratory events				
Hemoglobin, g/dL	10.0 (8.6, 11.5)	8.2 (7.5, 9.0)	11.0 (9.9, 12.2)	<0.001
RDW, %	15.0 (13.9, 16.8)	17.2 (15.6, 18.8)	14.3 (13.5, 15.4)	<0.001
HRR	6.5 (5.3, 7.9)	4.9 (4.3, 5.4)	7.5 (6.6, 8.6)	<0.001
Platelets, 109/L	198.0 (145.0, 268.0)	200.0 (138.0, 289.0)	196.0 (149.0, 258.0)	0.630
WBC, 109/L	13.9 (10.0, 18.8)	13.3 (9.1, 18.7)	14.2 (10.5, 18.9)	<0.001
Albumin, g/dL	3.2 (3.2, 3.2)	3.2 (3.0, 3.2)	3.2 (3.2, 3.2)	<0.001
Anion gap, mmol/L	16.0 (13.0, 19.0)	16.0 (14.0, 20.0)	16.0 (13.0, 19.0)	<0.001
Bicarbonate, mmol/L	24.0 (22.0, 27.0)	24.0 (21.0, 27.0)	24.0 (22.0, 27.0)	0.012
Bun, mg/dL	28.0 (18.0, 46.0)	34.0 (21.0, 57.0)	25.0 (17.0, 39.0)	<0.001
Calcium, mg/dL	8.6 (8.1, 8.9)	8.6 (8.1, 8.9)	8.6 (8.2, 8.9)	<0.001
Chloride, mmol/L	106.0 (101.0, 110.0)	105.0 (100.0, 110.0)	106.0 (102.0, 110.0)	<0.001
Creatinine, mg/dL	1.3 (0.9, 2.1)	1.5 (1.0, 2.6)	1.2 (0.9, 1.8)	<0.001
Sodium, mmol/L	140.0 (137.0, 142.0)	140.0 (137.0, 143.0)	140.0 (137.0, 142.0)	0.001
Lymphocytes, 109/L	88.6 (2.2, 88.6)	88.6 (1.5, 88.6)	88.6 (2.8, 88.6)	<0.001
Monocytes, 109/L	34.9 (1.3, 35.3)	34.9 (1.1, 34.9)	34.9 (1.6, 38.9)	<0.001
Neutrophils, 109/L	642.2 (17.6, 753.6)	642.2 (14.2, 642.2)	642.2 (21.1, 824.8)	<0.001
INR	1.5 (1.3, 2.0)	1.6 (1.3, 2.3)	1.4 (1.2, 2.0)	<0.001
PT, s	16.5 (14.1, 21.8)	17.7 (14.8, 24.1)	15.9 (13.8, 21.6)	<0.001
APTT, s	36.4 (30.4, 48.0)	37.6 (31.3, 49.6)	35.6 (29.9, 48.0)	<0.001
ALT, IU/L	146.0 (23.0, 146.0)	67.0 (19.0, 146.0)	146.0 (26.0, 146.0)	<0.001
AST, U/L	218.5 (35.0, 255.0)	105.5 (30.0, 255.0)	255.0 (39.0, 255.0)	<0.001
CK-MB, U/L	19.0 (8.0, 19.0)	19.0 (7.0, 19.0)	19.0 (9.0, 19.0)	<0.001
Glucose, mg/dL	131.5 (115.3, 158.6)	130.6 (112.0, 158.8)	132.0 (116.7, 158.5)	0.002
Scores				
APSIII	54.0 (40.0, 73.0)	59.0 (45.0, 76.0)	52.0 (38.0, 71.0)	<0.001
SOFA	3.0 (2.0, 5.0)	3.0 (2.0, 5.0)	3.0 (2.0, 4.0)	<0.001
GCS	13.0 (9.0, 15.0)	13.0 (9.0, 14.0)	13.0 (9.0, 15.0)	0.683
OASIS	36.0 (30.0, 43.0)	36.0 (30.0, 43.0)	36.0 (30.0, 43.0)	0.120
Outcomes				
28-day mortality, *n* (%)	1660 (18.0)	718 (21.7)	942 (15.9)	<0.001
60-day mortality, *n* (%)	1850 (20.0)	795 (24)	1055 (17.8)	<0.001
90-day mortality, *n* (%)	1913 (20.7)	823 (24.8)	1090 (18.4)	<0.001
In-hospital mortality, *n* (%)	1702 (18.4)	727 (22)	975 (16.5)	<0.001
Length of ICU stay, days	4.0 (2.0, 7.0)	4.0 (2.0, 7.0)	3.0 (2.0, 7.0)	0.008
Length of hospital stay, days	9.0 (6.0, 16.0)	10.0 (6.0, 16.0)	9.0 (6.0, 16.0)	0.134

**Table 3:** Multivariable Cox regression analyses for in-hospital mortality in septic patients with atrial fibrillation before and after PSM.

Characteristic	Non-Adjust Model		Model I		Model II		Model III	
	HR (95% CI)	*p*-Value	HR (95% CI)	*p*-Value	HR (95% CI)	*p*-Value	HR (95% CI)	*p*-Value
Before PSM								
HRR	0.919 (0.892~0.946)	<0.001	0.915 (0.888~0.942)	<0.001	0.834 (0.795~0.875)	<0.001	0.873 (0.829~0.920)	<0.001
High HRR (≥5.877)	1 (Ref.)		1 (Ref.)		1 (Ref.)		1 (Ref.)	
Low HRR (<5.877)	1.425 (1.280~1.586)	<0.001	1.432 (1.286~1.596)	<0.001	1.561 (1.352~1.803)	<0.001	1.412 (1.210~1.648)	<0.001
After PSM								
HRR	0.931 (0.895~0.968)	<0.001	0.930 (0.894~0.968)	0.0003	0.887 (0.835~0.943)	0.0001	0.902 (0.846~0.962)	0.0006
High HRR (≥5.877)	1 (Ref.)		1 (Ref.)		1 (Ref.)		1 (Ref.)	
Low HRR (<5.877)	1.321 (1.159~1.504)	<0.001	1.320 (1.158~1.504)	<0.001	1.366 (1.149~1.623)	<0.001	1.374 (1.144~1.649)	<0.001

**Table 4:** The clinical characteristics of septic patients with atrial fibrillation after PSM.

Characteristic	After PSM			
	All Patients	Low HRR < 5.877	High HRR ≥ 5.877	*p*
N	5862	2931	2931	
Demographic				
Female, *n* (%)	2388 (40.7)	1197 (40.8)	1191 (40.6)	0.894
Age, years	76.0 (67.0, 84.0)	76.0 (68.0, 84.0)	76.0 (67.0, 84.0)	0.345
Ethnicity, *n* (%)				0.622
Asian	151 (2.6)	77 (2.6)	74 (2.5)	
White	4433 (75.6)	2228 (76)	2205 (75.2)	
Black	393 (6.7)	201 (6.9)	192 (6.6)	
Other	885 (15.1)	425 (14.5)	460 (15.7)	
Vital signs				
HR, beats/minute	84 (75.0, 97.0)	85 (75.0, 97.0)	84 (75.0, 96.0)	0.091
SBP, mmHg	110 (103.0, 119.0)	110 (103.0, 119.0)	110 (103.0, 118.0)	0.531
DBP, mmHg	57 (52.0, 63.0)	57 (52.0, 63.0)	58 (52.0, 63.0)	0.898
MBP, mmHg	73 (68.0, 78.0)	73 (67.5, 78.0)	72 (68.0, 77.0)	0.911
RR, times/minute	19 (17.0, 22.0)	19 (17.0, 22.0)	19 (17.0, 22.0)	0.831
Temperature, °C	36.8 (36.5, 37.0)	36.8 (36.6, 37.0)	36.8 (36.5, 37.0)	0.958
SpO2, %	97 (96.0, 99.0)	97 (96.0, 99.0)	97 (96.0, 99.0)	0.758
Comorbidities, *n* (%)				
Myocardial infarct	1363 (23.3)	704 (24)	659 (22.5)	0.174
Congestive heart failure	3091 (52.7)	1556 (53.1)	1535 (52.4)	0.601
Peripheral vascular disease	992 (16.9)	510 (17.4)	482 (16.4)	0.347
Cerebrovascular disease	927 (15.8)	470 (16)	457 (15.6)	0.668
Dementia	312 (5.3)	153 (5.2)	159 (5.4)	0.771
Chronic pulmonary disease	1957 (33.4)	982 (33.5)	975 (33.3)	0.868
Rheumatic disease	247 (4.2)	121 (4.1)	126 (4.3)	0.795
Peptic ulcer disease	178 (3.0)	84 (2.9)	94 (3.2)	0.493
Paraplegia	571 (9.7)	291 (9.9)	280 (9.6)	0.66
Renal disease	250 (4.3)	111 (3.8)	139 (4.7)	0.081
Malignant cancer	1937 (33.0)	976 (33.3)	961 (32.8)	0.697
Metastatic solid tumor	726 (12.4)	355 (12.1)	371 (12.7)	0.552
Diabetes	198 (3.4)	107 (3.7)	91 (3.1)	0.278
Hypertension	280 (4.8)	132 (4.5)	148 (5)	0.358
Therapies, *n* (%)				
Warfarin	497 (8.5)	244 (8.3)	253 (8.6)	0.708
Amiodarone	1116 (19.0)	486 (16.6)	630 (21.5)	<0.001
Dopamine	343 (5.9)	155 (5.3)	188 (6.4)	0.075
Epinephrine	643 (11.0)	306 (10.4)	337 (11.5)	0.21
Vasopressin	888 (15.1)	414 (14.1)	474 (16.2)	0.032
Antibiotic	1101 (18.8)	518 (17.7)	583 (19.9)	0.032
Ventilation	3075 (52.5)	1508 (51.5)	1567 (53.5)	0.129
Laboratory events				
Hemoglobin, g/dL	9.4 (8.2, 10.8)	8.3 (7.5, 9.1)	10.7 (9.7, 11.9)	<0.001
RDW, %	15.6 (14.2, 17.4)	17.1 (15.6, 18.7)	14.5 (13.6, 15.6)	<0.001
HRR	5.9 (5.0, 7.2)	5.0 (4.4, 5.4)	7.2 (6.5, 8.2)	<0.001
Platelets, 109/L	198.0 (143.0, 271.0)	201.0 (140.0, 286.0)	194.0 (145.0, 258.0)	0.141
WBC, 109/L	13.9 (9.8, 18.9)	13.3 (9.1, 18.5)	14.3 (10.5, 19.2)	<0.001
Albumin, g/dL	3.2 (3.2, 3.2)	3.2 (3.2, 3.2)	3.2 (3.2, 3.2)	0.609
Anion gap, mmol/L	16.0 (13.0, 19.0)	16.0 (13.0, 19.0)	16.0 (13.0, 19.0)	0.785
Bicarbonate, mmol/L	24.0 (22.0, 27.0)	24.0 (22.0, 27.0)	24.0 (22.0, 27.0)	0.209
BUN, mg/dL	31.0 (20.0, 50.0)	33.0 (21.0, 53.0)	29.0 (19.0, 47.0)	<0.001
Calcium, mg/dL	8.6 (8.1, 8.9)	8.6 (8.1, 8.9)	8.6 (8.1, 8.9)	0.393
Chloride, mmol/L	106.0 (101.0, 110.0)	105.0 (101.0, 110.0)	106.0 (102.0, 110.0)	<0.001
Creatinine, mg/dL	1.4 (1.0, 2.3)	1.4 (1.0, 2.4)	1.3 (0.9, 2.2)	0.003
Sodium, mmol/L	140.0 (137.0, 142.0)	140.0 (137.0, 143.0)	140.0 (137.0, 142.0)	0.130
Lymphocytes, 109/L	88.6 (2.0, 88.6)	88.6 (1.6, 88.6)	88.6 (2.3, 88.6)	0.004
Monocytes, 109/L	34.9 (1.2, 34.9)	34.9 (1.1, 34.9)	34.9 (1.4, 36.2)	<0.001
Neutrophils, 109/L	642.2 (16.3, 714.6)	642.2 (14.3, 647.4)	642.2 (19.2, 783.0)	<0.001
INR	1.6 (1.3, 2.1)	1.6 (1.3, 2.2)	1.5 (1.3, 2.0)	<0.001
PT, s	16.9 (14.4, 22.8)	17.4 (14.7, 23.4)	16.5 (14.1, 21.8)	<0.001
APTT, s	36.9 (30.6, 48.6)	37.2 (31.1, 48.3)	36.5 (30.2, 49.0)	0.050
ALT, IU/L	126.0 (22.0, 146.0)	76.0 (20.0, 146.0)	146.0 (25.0, 146.0)	<0.001
AST, U/L	176.0 (35.0, 255.0)	119.0 (31.0, 255.0)	255.0 (39.0, 255.0)	<0.001
CK-MB, U/L	19.0 (7.0, 19.0)	19.0 (7.0, 19.0)	19.0 (7.0, 19.0)	0.928
Glucose, mg/dL	131.4 (114.6, 159.7)	131.0 (112.5, 158.8)	132.2 (116.0, 160.4)	0.037
Scores				
APSIII	57.0 (43.0, 75.0)	57.0 (44.0, 73.0)	57.0 (42.0, 77.0)	0.633
SOFA	3.0 (2.0, 5.0)	3.0 (2.0, 5.0)	3.0 (2.0, 5.0)	0.636
GCS	13.0 (9.0, 14.0)	14.0 (10.0, 15.0)	13.0 (9.0, 14.0)	<0.001
OASIS	36.0 (30.0, 43.0)	36.0 (30.0, 42.0)	37.0 (31.0, 44.0)	<0.001
Outcomes				
28-day mortality, *n* (%)	1125 (19.2)	635 (21.7)	490 (16.7)	<0.001
60-day mortality, *n* (%)	1237 (21.1)	684 (23.3)	553 (18.9)	<0.001
90-day mortality, *n* (%)	1275 (21.8)	705 (24.1)	570 (19.4)	<0.001
In-hospital mortality, *n* (%)	1137 (19.4)	632 (21.6)	505 (17.2)	<0.001
Length of ICU stay, days	4.0 (2.0, 7.0)	3.0 (2.0, 7.0)	4.0 (2.0, 7.0)	0.326
Length of hospital stay, days	9.0 (6.0, 15.0)	9.0 (6.0, 14.0)	9.0 (6.0, 16.0)	0.004

## Data Availability

All data in the article can be obtained from the MIMIC-IV database (https://mimic.physionet.org/) (accessed on 17 October 2021).

## Supplementary Materials

The following supporting information can be downloaded at: https://www.mdpi.com/article/10.3390/jcdd9110400/s1. Figure S1: The ROC curve of HRR; Table S1: The best cut-off value, specificity, sensitivity, and Youden Index of HRR; Table S2: Univariable Cox regression analyses for in-hospital mortality in septic patients with atrial fibrillation.

## Abbreviations

HR: heart rate; SBP, systolic blood pressure; DBP, diastolic blood pressure; MBP, mean blood pressure; RR, respiratory rate; SpO2, percutaneous oxygen saturation; RDW, red cell distribution width; HRR, hemoglobin/red cell distribution width; BUN, blood urea nitrogen; INR, international normalized ratio; PT, prothrombin time; APTT, activated partial thromboplastin time; ALT, alanine aminotransferase; AST, aspartate aminotransferase; CK-MB, creatine kinase-MB isoenzyme; APS III, acute physiology score III; SOFA, sequential organ failure assessment score; GCS, Glasgow coma score; OASIS, oxford acute severity of illness score; ICU, Intensive care unit; PSM, propensity score matching; HRR, hemoglobin-to-red cell distribution width ratio; OR, odds ratio; CI, confidence interval; K-M, Kaplan-Meier; SMD, standardized mean difference.
