# Supplementary material for: Relationship between the Hemoglobin-to-Red Cell Distribution Width Ratio and All-Cause Mortality in Septic Patients with Atrial Fibrillation: Based on Propensity Score Matching Method
Source: J Cardiovasc Dev Dis. 2022 Nov 18;9(11):400. doi: 10.3390/jcdd9110400 (PMC9696521; doi:10.3390/jcdd9110400)
Supplement: Supplementary file 1 [file jcdd-09-00400-s001.zip › jcdd-1954214-Supplementary.pdf]

Supplementary Materials

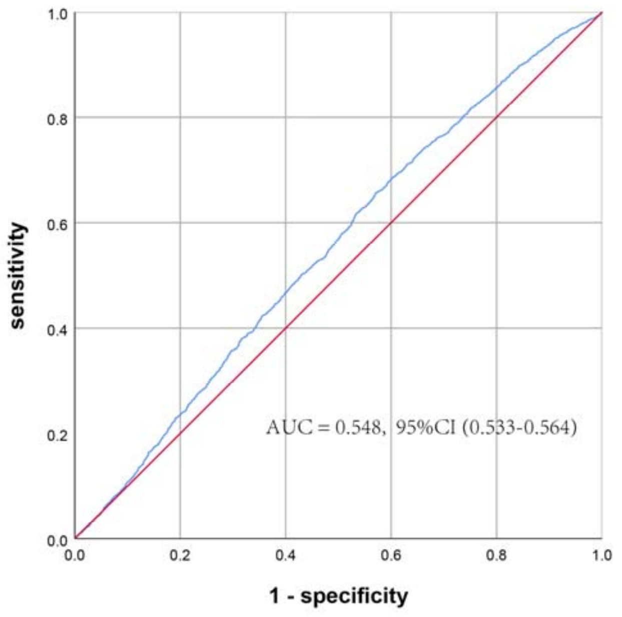

Figure S1. The ROC curve of HRR.

Table S1: The best cut-off value, specificity, sensitivity and Youden Index of HRR.

Cut-off value	Sensitivity	1 - Specificity	Youden Index
5.877075628	0.656	0.571	0.0850
5.878120487	0.656	0.571	0.0850
5.884033613	0.655	0.57	0.0850
5.885895118	0.655	0.57	0.0850
5.886300386	0.655	0.57	0.0850
5.889990756	0.654	0.569	0.0850
5.722717459	0.687	0.603	0.0840
5.723287888	0.687	0.603	0.0840
5.734105344	0.684	0.6	0.0840
5.734779926	0.684	0.6	0.0840
5.866294227	0.659	0.575	0.0840
5.86746507	0.659	0.575	0.0840
5.870807254	0.658	0.574	0.0840
5.873109449	0.657	0.573	0.0840
5.874562937	0.657	0.573	0.0840
5.875997437	0.656	0.572	0.0840
5.878583129	0.655	0.571	0.0840

5.88057041	0.655	0.571	0.0840
5.882352941	0.655	0.571	0.0840
5.888047687	0.654	0.57	0.0840
5.890941914	0.653	0.569	0.0840
5.89168238	0.653	0.569	0.0840
5.892374517	0.653	0.569	0.0840
5.893448439	0.653	0.569	0.0840
6.064665472	0.617	0.533	0.0840
6.065740741	0.617	0.533	0.0840
5.717967599	0.687	0.604	0.0830
5.721935853	0.687	0.604	0.0830
5.722382787	0.687	0.604	0.0830
5.723911071	0.686	0.603	0.0830
5.724387806	0.686	0.603	0.0830
5.729818684	0.685	0.602	0.0830
5.731350735	0.685	0.602	0.0830
5.733639144	0.684	0.601	0.0830
5.735864113	0.683	0.6	0.0830
5.737069513	0.683	0.6	0.0830
5.738674944	0.683	0.6	0.0830
5.740192856	0.683	0.6	0.0830
5.741338112	0.683	0.6	0.0830
5.742589364	0.683	0.6	0.0830
5.762150634	0.679	0.596	0.0830
5.763300377	0.679	0.596	0.0830
5.771620326	0.678	0.595	0.0830
5.772084902	0.678	0.595	0.0830
5.864035938	0.66	0.577	0.0830
5.864429592	0.659	0.576	0.0830
5.865291721	0.659	0.576	0.0830
5.868914345	0.658	0.575	0.0830
5.869565217	0.658	0.575	0.0830
5.870105992	0.658	0.575	0.0830
5.875353107	0.656	0.573	0.0830
5.894996746	0.652	0.569	0.0830
5.896694827	0.652	0.569	0.0830
5.91430632	0.649	0.566	0.0830
5.915063552	0.649	0.566	0.0830
6.012865062	0.628	0.545	0.0830
6.026065546	0.626	0.543	0.0830
6.027883027	0.625	0.542	0.0830
6.031140191	0.624	0.541	0.0830
6.032401941	0.624	0.541	0.0830

6.055379898	0.619	0.536	0.0830
6.057768509	0.618	0.535	0.0830
6.058608845	0.618	0.535	0.0830
6.059714795	0.617	0.534	0.0830
6.06155303	0.617	0.534	0.0830
6.062746063	0.617	0.534	0.0830
6.063754128	0.617	0.534	0.0830
6.067041199	0.616	0.533	0.0830
6.070744902	0.616	0.533	0.0830
6.075011721	0.616	0.533	0.0830
6.076436222	0.616	0.533	0.0830
6.079772727	0.615	0.532	0.0830
6.080540541	0.615	0.532	0.0830
6.081476213	0.615	0.532	0.0830
6.082893714	0.615	0.532	0.0830
6.084126717	0.615	0.532	0.0830
6.085646936	0.615	0.532	0.0830
5.72491426	0.685	0.603	0.0820
5.725498646	0.685	0.603	0.0820
5.727224834	0.685	0.603	0.0820
5.732095697	0.684	0.602	0.0820
5.732908705	0.684	0.602	0.0820
5.743962047	0.682	0.6	0.0820
5.745268602	0.682	0.6	0.0820
5.746491395	0.682	0.6	0.0820
5.747814715	0.682	0.6	0.0820
5.752161079	0.681	0.599	0.0820
5.753056415	0.68	0.598	0.0820
5.753807301	0.68	0.598	0.0820
5.754453463	0.68	0.598	0.0820
5.755265467	0.68	0.598	0.0820
5.756694856	0.68	0.598	0.0820
5.759327987	0.679	0.597	0.0820
5.760541537	0.679	0.597	0.0820
5.764297386	0.678	0.596	0.0820
5.76556462	0.678	0.596	0.0820
5.768925965	0.678	0.596	0.0820
5.773502805	0.677	0.595	0.0820
5.775024478	0.677	0.595	0.0820
5.775899293	0.677	0.595	0.0820
5.777087647	0.676	0.594	0.0820
5.778499278	0.676	0.594	0.0820
5.7797838	0.676	0.594	0.0820

5.781451821	0.675	0.593	0.0820
5.782722728	0.675	0.593	0.0820
5.855808375	0.661	0.579	0.0820
5.856748224	0.661	0.579	0.0820
5.858507734	0.661	0.579	0.0820
5.860970788	0.66	0.578	0.0820
5.862971656	0.66	0.578	0.0820
5.898358236	0.651	0.569	0.0820
5.899950847	0.651	0.569	0.0820
5.901130231	0.651	0.569	0.0820
5.902208561	0.651	0.569	0.0820
5.904144794	0.651	0.569	0.0820
5.907761829	0.65	0.568	0.0820
5.910520297	0.65	0.568	0.0820
5.912179222	0.649	0.567	0.0820
5.913193627	0.649	0.567	0.0820
5.915861662	0.648	0.566	0.0820
5.916695065	0.648	0.566	0.0820
5.684192372	0.692	0.61	0.0820
6.013528955	0.627	0.545	0.0820
6.014511804	0.627	0.545	0.0820
6.015331297	0.627	0.545	0.0820
6.025242873	0.626	0.544	0.0820
6.026943663	0.625	0.543	0.0820
6.02889028	0.624	0.542	0.0820
6.029781259	0.624	0.542	0.0820
6.030342552	0.624	0.542	0.0820
6.033288702	0.623	0.541	0.0820
6.034001156	0.623	0.541	0.0820
6.034992859	0.623	0.541	0.0820
6.036044162	0.623	0.541	0.0820
6.037160607	0.623	0.541	0.0820
6.040967562	0.622	0.54	0.0820
6.042416067	0.621	0.539	0.0820
6.043560756	0.621	0.539	0.0820
6.048145644	0.62	0.538	0.0820
6.048884906	0.62	0.538	0.0820
6.050169065	0.62	0.538	0.0820
6.051118733	0.619	0.537	0.0820
6.051956815	0.619	0.537	0.0820
6.053526674	0.619	0.537	0.0820
6.056740443	0.618	0.536	0.0820
6.077677225	0.615	0.533	0.0820

6.078988414	0.615	0.533	0.0820
5.705701913	0.687	0.606	0.0810
5.710084034	0.687	0.606	0.0810
5.714285714	0.687	0.606	0.0810
5.749251497	0.681	0.6	0.0810
5.750816993	0.681	0.6	0.0810
5.758369031	0.679	0.598	0.0810
5.780468769	0.675	0.594	0.0810
5.784423408	0.674	0.593	0.0810
5.787593985	0.674	0.593	0.0810
5.789473684	0.674	0.593	0.0810
5.790911705	0.673	0.592	0.0810
5.792516327	0.673	0.592	0.0810
5.855092719	0.661	0.58	0.0810
5.90577604	0.65	0.569	0.0810
5.906236508	0.65	0.569	0.0810
5.917763555	0.647	0.566	0.0810
5.918953788	0.647	0.566	0.0810
5.920296431	0.647	0.566	0.0810
5.925048271	0.646	0.565	0.0810
5.925925926	0.646	0.565	0.0810
5.932768362	0.644	0.563	0.0810
5.570949185	0.712	0.631	0.0810
5.573144788	0.711	0.63	0.0810
5.574241568	0.711	0.63	0.0810
5.57523511	0.711	0.63	0.0810
5.576340326	0.711	0.63	0.0810
5.665703276	0.696	0.615	0.0810
5.669446451	0.695	0.614	0.0810
5.6704174	0.695	0.614	0.0810
5.671186749	0.694	0.613	0.0810
5.672078205	0.694	0.613	0.0810
5.673136743	0.694	0.613	0.0810
5.673958084	0.694	0.613	0.0810
5.677251344	0.693	0.612	0.0810
5.67821585	0.693	0.612	0.0810
5.683256673	0.692	0.611	0.0810
5.685603008	0.691	0.61	0.0810
5.686887255	0.691	0.61	0.0810
5.688061377	0.691	0.61	0.0810
5.689138963	0.691	0.61	0.0810
5.690572267	0.69	0.609	0.0810
5.691898527	0.69	0.609	0.0810

5.692869175	0.69	0.609	0.0810
5.693937551	0.69	0.609	0.0810
5.694904341	0.69	0.609	0.0810
5.695783385	0.69	0.609	0.0810
5.696586114	0.689	0.608	0.0810
5.697322058	0.689	0.608	0.0810
5.698299575	0.689	0.608	0.0810
5.701575042	0.689	0.608	0.0810
5.704913096	0.688	0.607	0.0810
5.705324839	0.688	0.607	0.0810
5.936491935	0.643	0.562	0.0810
6.011570358	0.628	0.547	0.0810
6.01208735	0.628	0.547	0.0810
6.012464083	0.628	0.547	0.0810
6.015942581	0.626	0.545	0.0810
6.019179805	0.626	0.545	0.0810
6.02274563	0.626	0.545	0.0810
6.024118267	0.626	0.545	0.0810
6.038348444	0.622	0.541	0.0810
6.039614748	0.622	0.541	0.0810
6.044576892	0.62	0.539	0.0810
6.045854684	0.62	0.539	0.0810
6.04720791	0.62	0.539	0.0810
6.088350056	0.613	0.532	0.0810
6.089984577	0.613	0.532	0.0810
6.090798061	0.613	0.532	0.0810
6.092042895	0.613	0.532	0.0810
5.793777361	0.672	0.592	0.0800
5.79516317	0.672	0.592	0.0800
5.796277997	0.672	0.592	0.0800
5.797959009	0.672	0.592	0.0800
5.799408284	0.672	0.592	0.0800
5.820049813	0.668	0.588	0.0800
5.822070015	0.668	0.588	0.0800
5.822503516	0.668	0.588	0.0800
5.823157111	0.667	0.587	0.0800
5.823852618	0.667	0.587	0.0800
5.825995197	0.667	0.587	0.0800
5.828017714	0.667	0.587	0.0800
5.828396144	0.666	0.586	0.0800
5.830952381	0.666	0.586	0.0800
5.833333333	0.665	0.585	0.0800
5.835585586	0.665	0.585	0.0800

5.837994063	0.665	0.585	0.0800
5.838717746	0.664	0.584	0.0800
5.839463087	0.664	0.584	0.0800
5.840792079	0.664	0.584	0.0800
5.844783236	0.663	0.583	0.0800
5.846202582	0.663	0.583	0.0800
5.847473876	0.663	0.583	0.0800
5.84850491	0.663	0.583	0.0800
5.850454228	0.662	0.582	0.0800
5.85206229	0.662	0.582	0.0800
5.854290408	0.661	0.581	0.0800
5.922309755	0.646	0.566	0.0800
5.923868748	0.646	0.566	0.0800
5.927034819	0.645	0.565	0.0800
5.928357571	0.645	0.565	0.0800
5.934408602	0.643	0.563	0.0800
5.467146283	0.73	0.65	0.0800
5.571973828	0.711	0.631	0.0800
5.577406262	0.71	0.63	0.0800
5.57806037	0.71	0.63	0.0800
5.57858933	0.71	0.63	0.0800
5.579328757	0.71	0.63	0.0800
5.579910321	0.71	0.63	0.0800
5.591751556	0.708	0.628	0.0800
5.592662801	0.708	0.628	0.0800
5.664537774	0.696	0.616	0.0800
5.667557932	0.695	0.615	0.0800
5.668619503	0.695	0.615	0.0800
5.67491649	0.693	0.613	0.0800
5.676379505	0.693	0.613	0.0800
5.679742859	0.692	0.612	0.0800
5.681145777	0.692	0.612	0.0800
5.681818182	0.692	0.612	0.0800
5.682439146	0.692	0.612	0.0800
5.704461669	0.688	0.608	0.0800
5.938293147	0.642	0.562	0.0800
5.939240117	0.642	0.562	0.0800
5.939621782	0.642	0.562	0.0800
5.940513047	0.642	0.562	0.0800
5.941602728	0.642	0.562	0.0800
5.96213569	0.638	0.558	0.0800
5.96329417	0.638	0.558	0.0800
5.966825513	0.637	0.557	0.0800

5.967741935	0.637	0.557	0.0800
5.968367092	0.637	0.557	0.0800
5.970107635	0.637	0.557	0.0800
5.976992496	0.634	0.554	0.0800
5.978244805	0.634	0.554	0.0800
5.981543974	0.634	0.554	0.0800
5.985658476	0.633	0.553	0.0800
5.987457734	0.632	0.552	0.0800
6.005617978	0.628	0.548	0.0800
6.093232616	0.612	0.532	0.0800
6.094212278	0.612	0.532	0.0800
6.095282484	0.612	0.532	0.0800
6.096725693	0.611	0.531	0.0800
6.098425878	0.611	0.531	0.0800
6.099959855	0.611	0.531	0.0800
6.106066693	0.61	0.53	0.0800
5.800763359	0.671	0.592	0.0790
5.801997927	0.671	0.592	0.0790
5.80333247	0.671	0.592	0.0790
5.804396753	0.671	0.592	0.0790
5.807417423	0.67	0.591	0.0790
5.80981717	0.669	0.59	0.0790
5.811655405	0.669	0.59	0.0790
5.813226744	0.669	0.59	0.0790
5.813953488	0.669	0.59	0.0790
5.815473476	0.668	0.589	0.0790
5.817587641	0.668	0.589	0.0790
5.838329803	0.664	0.585	0.0790
5.842478826	0.663	0.584	0.0790
5.843764669	0.663	0.584	0.0790
5.852965632	0.661	0.582	0.0790
5.929401993	0.644	0.565	0.0790
5.93063352	0.644	0.565	0.0790
5.931203516	0.644	0.565	0.0790
5.931787969	0.644	0.565	0.0790
5.411361805	0.742	0.663	0.0790
5.41264927	0.742	0.663	0.0790
5.413773287	0.742	0.663	0.0790
5.41783054	0.74	0.661	0.0790
5.419420871	0.74	0.661	0.0790
5.420767038	0.74	0.661	0.0790
5.422110979	0.74	0.661	0.0790
5.423132012	0.74	0.661	0.0790

5.425279041	0.739	0.66	0.0790
5.426982473	0.739	0.66	0.0790
5.427853553	0.739	0.66	0.0790
5.435340022	0.737	0.658	0.0790
5.436069523	0.737	0.658	0.0790
5.436870805	0.737	0.658	0.0790
5.449269999	0.734	0.655	0.0790
5.449586826	0.734	0.655	0.0790
5.449867725	0.734	0.655	0.0790
5.456864564	0.733	0.654	0.0790
5.465351662	0.731	0.652	0.0790
5.466252588	0.73	0.651	0.0790
5.467803097	0.729	0.65	0.0790
5.468365148	0.729	0.65	0.0790
5.46918163	0.729	0.65	0.0790
5.470100747	0.729	0.65	0.0790
5.472335543	0.728	0.649	0.0790
5.473328592	0.728	0.649	0.0790
5.474272273	0.728	0.649	0.0790
5.475525406	0.728	0.649	0.0790
5.482899339	0.727	0.648	0.0790
5.48465393	0.727	0.648	0.0790
5.485912698	0.726	0.647	0.0790
5.492957746	0.725	0.646	0.0790
5.493392453	0.725	0.646	0.0790
5.495005183	0.725	0.646	0.0790
5.496882383	0.724	0.645	0.0790
5.498538012	0.724	0.645	0.0790
5.508501713	0.722	0.643	0.0790
5.509593059	0.722	0.643	0.0790
5.510783859	0.722	0.643	0.0790
5.512092075	0.722	0.643	0.0790
5.513167013	0.721	0.642	0.0790
5.514109698	0.721	0.642	0.0790
5.514928699	0.721	0.642	0.0790
5.516196447	0.721	0.642	0.0790
5.569241264	0.712	0.633	0.0790
5.570045026	0.712	0.633	0.0790
5.580752923	0.709	0.63	0.0790
5.582108717	0.709	0.63	0.0790
5.586325439	0.708	0.629	0.0790
5.589148703	0.708	0.629	0.0790
5.590729981	0.708	0.629	0.0790

5.593812967	0.707	0.628	0.0790
5.595130258	0.707	0.628	0.0790
5.5966696	0.707	0.628	0.0790
5.597655182	0.706	0.627	0.0790
5.598913043	0.706	0.627	0.0790
5.601204819	0.706	0.627	0.0790
5.604745573	0.705	0.626	0.0790
5.623150888	0.703	0.624	0.0790
5.625	0.703	0.624	0.0790
5.64331494	0.699	0.62	0.0790
5.644666535	0.699	0.62	0.0790
5.646110057	0.699	0.62	0.0790
5.647956893	0.699	0.62	0.0790
5.663493133	0.696	0.617	0.0790
5.942443064	0.641	0.562	0.0790
5.943456543	0.641	0.562	0.0790
5.944250194	0.641	0.562	0.0790
5.945195195	0.641	0.562	0.0790
5.946657183	0.641	0.562	0.0790
5.94754042	0.641	0.562	0.0790
5.950143667	0.64	0.561	0.0790
5.951650599	0.64	0.561	0.0790
5.953069089	0.64	0.561	0.0790
5.956251494	0.639	0.56	0.0790
5.958175459	0.639	0.56	0.0790
5.959584505	0.638	0.559	0.0790
5.960428017	0.638	0.559	0.0790
5.961064797	0.638	0.559	0.0790
5.964882256	0.637	0.558	0.0790
5.971722622	0.636	0.557	0.0790
5.972688292	0.636	0.557	0.0790
5.973590168	0.635	0.556	0.0790
5.974434371	0.635	0.556	0.0790
5.975226262	0.635	0.556	0.0790
5.975970559	0.634	0.555	0.0790
5.984550227	0.633	0.554	0.0790
5.985124972	0.633	0.554	0.0790
5.986378799	0.632	0.553	0.0790
5.987051626	0.632	0.553	0.0790
5.987839137	0.631	0.552	0.0790
5.988198023	0.631	0.552	0.0790
5.988536329	0.631	0.552	0.0790
6.101161923	0.61	0.531	0.0790

6.102129509	0.61	0.531	0.0790
6.103230103	0.61	0.531	0.0790
6.104273633	0.61	0.531	0.0790
6.10495716	0.61	0.531	0.0790
6.10712639	0.609	0.53	0.0790
6.107583491	0.609	0.53	0.0790
6.109447771	0.609	0.53	0.0790
6.111111111	0.609	0.53	0.0790
6.112698413	0.608	0.529	0.0790
6.114467698	0.608	0.529	0.0790
6.114878798	0.608	0.529	0.0790
6.116377486	0.608	0.529	0.0790
5.805524657	0.67	0.592	0.0780
5.806451613	0.67	0.592	0.0780
5.808603381	0.669	0.591	0.0780
5.403012779	0.744	0.666	0.0780
5.404566057	0.743	0.665	0.0780
5.41418869	0.741	0.663	0.0780
5.415515654	0.741	0.663	0.0780
5.416666667	0.741	0.663	0.0780
5.429339478	0.738	0.66	0.0780
5.430285552	0.738	0.66	0.0780
5.431281171	0.738	0.66	0.0780
5.432584816	0.737	0.659	0.0780
5.433298439	0.737	0.659	0.0780
5.43415431	0.737	0.659	0.0780
5.438048246	0.736	0.658	0.0780
5.439078465	0.736	0.658	0.0780
5.440368455	0.736	0.658	0.0780
5.441676671	0.736	0.658	0.0780
5.443412478	0.735	0.657	0.0780
5.44440658	0.735	0.657	0.0780
5.446393686	0.735	0.657	0.0780
5.448018528	0.735	0.657	0.0780
5.448496905	0.734	0.656	0.0780
5.448909873	0.734	0.656	0.0780
5.452272727	0.733	0.655	0.0780
5.454545455	0.733	0.655	0.0780
5.459476894	0.732	0.654	0.0780
5.459946407	0.732	0.654	0.0780
5.460324508	0.732	0.654	0.0780
5.460759612	0.732	0.654	0.0780
5.465712777	0.73	0.652	0.0780

5.471143174	0.728	0.65	0.0780
5.477821265	0.727	0.649	0.0780
5.479839022	0.727	0.649	0.0780
5.48107685	0.727	0.649	0.0780
5.485575589	0.726	0.648	0.0780
5.486957995	0.725	0.647	0.0780
5.489000478	0.725	0.647	0.0780
5.491576912	0.725	0.647	0.0780
5.496435974	0.724	0.646	0.0780
5.50147929	0.723	0.645	0.0780
5.503157142	0.723	0.645	0.0780
5.503615837	0.723	0.645	0.0780
5.504746973	0.723	0.645	0.0780
5.505973546	0.723	0.645	0.0780
5.506787745	0.722	0.644	0.0780
5.507633884	0.722	0.644	0.0780
5.518360949	0.72	0.642	0.0780
5.520476456	0.72	0.642	0.0780
5.521930226	0.72	0.642	0.0780
5.522821937	0.72	0.642	0.0780
5.561813752	0.713	0.635	0.0780
5.562522042	0.713	0.635	0.0780
5.563147094	0.713	0.635	0.0780
5.583618835	0.708	0.63	0.0780
5.602623259	0.705	0.627	0.0780
5.603350807	0.705	0.627	0.0780
5.604130169	0.705	0.627	0.0780
5.606601825	0.704	0.626	0.0780
5.608286858	0.704	0.626	0.0780
5.609110853	0.704	0.626	0.0780
5.610433604	0.704	0.626	0.0780
5.612007168	0.704	0.626	0.0780
5.613938244	0.704	0.626	0.0780
5.615705809	0.704	0.626	0.0780
5.620676494	0.703	0.625	0.0780
5.621108404	0.703	0.625	0.0780
5.62670765	0.702	0.624	0.0780
5.628578908	0.702	0.624	0.0780
5.628940794	0.702	0.624	0.0780
5.629384351	0.701	0.623	0.0780
5.630906769	0.701	0.623	0.0780
5.63254765	0.701	0.623	0.0780
5.638340818	0.7	0.622	0.0780

5.649102806	0.698	0.62	0.0780
5.649534082	0.698	0.62	0.0780
5.650945714	0.698	0.62	0.0780
5.654967159	0.697	0.619	0.0780
5.656533575	0.697	0.619	0.0780
5.659136048	0.697	0.619	0.0780
5.661071032	0.697	0.619	0.0780
5.662207654	0.696	0.618	0.0780
5.948539753	0.64	0.562	0.0780
5.953977849	0.639	0.561	0.0780
5.954627327	0.639	0.561	0.0780
5.989633301	0.63	0.552	0.0780
5.990778514	0.63	0.552	0.0780
5.995495495	0.63	0.552	0.0780
6.118034056	0.607	0.529	0.0780
6.118912019	0.607	0.529	0.0780
5.397276335	0.744	0.667	0.0770
5.398863636	0.744	0.667	0.0770
5.400534759	0.744	0.667	0.0770
5.401264686	0.744	0.667	0.0770
5.401879352	0.744	0.667	0.0770
5.406406406	0.742	0.665	0.0770
5.408106219	0.742	0.665	0.0770
5.409881968	0.742	0.665	0.0770
5.442607423	0.735	0.658	0.0770
5.462736891	0.731	0.654	0.0770
5.464798577	0.731	0.654	0.0770
5.523865669	0.719	0.642	0.0770
5.525395657	0.719	0.642	0.0770
5.52697699	0.719	0.642	0.0770
5.527794251	0.719	0.642	0.0770
5.552344252	0.714	0.637	0.0770
5.558526441	0.713	0.636	0.0770
5.563645028	0.712	0.635	0.0770
5.566045796	0.712	0.635	0.0770
5.568522047	0.712	0.635	0.0770
5.618438156	0.703	0.626	0.0770
5.633357105	0.7	0.623	0.0770
5.635083227	0.7	0.623	0.0770
5.636973764	0.7	0.623	0.0770
5.639316314	0.699	0.622	0.0770
5.640280262	0.699	0.622	0.0770
5.641741871	0.699	0.622	0.0770

5.652173913	0.697	0.62	0.0770
5.653467909	0.697	0.62	0.0770
6.119810782	0.606	0.529	0.0770
6.12071535	0.606	0.529	0.0770
6.121353724	0.606	0.529	0.0770
6.121972153	0.606	0.529	0.0770
6.12999232	0.603	0.526	0.0770
6.131169621	0.603	0.526	0.0770
6.133893557	0.602	0.525	0.0770
6.134711553	0.602	0.525	0.0770
5.370899471	0.752	0.676	0.0760
5.389415973	0.747	0.671	0.0760
5.389840656	0.746	0.67	0.0760
5.39137054	0.746	0.67	0.0760
5.392964292	0.746	0.67	0.0760
5.39504284	0.745	0.669	0.0760
5.395516145	0.745	0.669	0.0760
5.395861529	0.745	0.669	0.0760
5.3964325	0.744	0.668	0.0760
5.528398232	0.718	0.642	0.0760
5.529128959	0.718	0.642	0.0760
5.530069011	0.718	0.642	0.0760
5.538601377	0.716	0.64	0.0760
5.540054443	0.716	0.64	0.0760
5.541784974	0.715	0.639	0.0760
5.542512909	0.715	0.639	0.0760
5.544866071	0.715	0.639	0.0760
5.547160128	0.715	0.639	0.0760
5.548956718	0.714	0.638	0.0760
6.123022243	0.605	0.529	0.0760
6.123813257	0.605	0.529	0.0760
6.124515504	0.605	0.529	0.0760
6.125327225	0.604	0.528	0.0760
6.126207507	0.604	0.528	0.0760
6.128099944	0.603	0.527	0.0760
6.131991773	0.602	0.526	0.0760
6.132965009	0.602	0.526	0.0760
6.135666481	0.601	0.525	0.0760
6.136363636	0.601	0.525	0.0760
6.137147335	0.601	0.525	0.0760
5.357997266	0.755	0.68	0.0750
5.358983848	0.755	0.68	0.0750
5.361882312	0.754	0.679	0.0750

5.364410872	0.753	0.678	0.0750
5.365218496	0.753	0.678	0.0750
5.366542648	0.752	0.677	0.0750
5.368179578	0.752	0.677	0.0750
5.369748944	0.752	0.677	0.0750
5.371884498	0.751	0.676	0.0750
5.372737377	0.751	0.676	0.0750
5.373641994	0.751	0.676	0.0750
5.37457483	0.75	0.675	0.0750
5.375672043	0.75	0.675	0.0750
5.376614254	0.75	0.675	0.0750
5.37783615	0.75	0.675	0.0750
5.379267357	0.75	0.675	0.0750
5.386752137	0.747	0.672	0.0750
5.389055223	0.747	0.672	0.0750
5.39359891	0.745	0.67	0.0750
5.394338118	0.745	0.67	0.0750
5.394736842	0.745	0.67	0.0750
5.531320575	0.717	0.642	0.0750
5.531914894	0.717	0.642	0.0750
5.532624113	0.717	0.642	0.0750
5.533962264	0.717	0.642	0.0750
5.53515274	0.717	0.642	0.0750
5.536674347	0.716	0.641	0.0750
5.540970907	0.715	0.64	0.0750
5.54769523	0.714	0.639	0.0750
5.548166151	0.714	0.639	0.0750
5.548583792	0.714	0.639	0.0750
6.126964097	0.603	0.528	0.0750
6.13858577	0.6	0.525	0.0750
6.139795692	0.6	0.525	0.0750
5.323627985	0.761	0.687	0.0740
5.325381141	0.761	0.687	0.0740
5.339373464	0.758	0.684	0.0740
5.341261999	0.758	0.684	0.0740
5.34204033	0.758	0.684	0.0740
5.359296573	0.754	0.68	0.0740
5.360461454	0.754	0.68	0.0740
5.362723666	0.753	0.679	0.0740
5.363683451	0.753	0.679	0.0740
5.379931897	0.749	0.675	0.0740
5.380275871	0.749	0.675	0.0740
5.380693582	0.748	0.674	0.0740

5.382783883	0.748	0.674	0.0740
5.384615385	0.748	0.674	0.0740
6.140827613	0.599	0.525	0.0740
6.141518316	0.599	0.525	0.0740
6.141932132	0.599	0.525	0.0740
5.314485514	0.762	0.689	0.0730
5.315570505	0.762	0.689	0.0730
5.327277055	0.76	0.687	0.0730
5.328707261	0.76	0.687	0.0730
5.329144343	0.76	0.687	0.0730
5.329505824	0.759	0.686	0.0730
5.331501832	0.759	0.686	0.0730
5.335060449	0.758	0.685	0.0730
5.336933108	0.758	0.685	0.0730
5.337458245	0.758	0.685	0.0730
5.342988602	0.757	0.684	0.0730
5.343713397	0.757	0.684	0.0730
5.344913647	0.757	0.684	0.0730
5.346567086	0.757	0.684	0.0730
5.352526926	0.756	0.683	0.0730
5.356296547	0.755	0.682	0.0730
6.142494561	0.598	0.525	0.0730
6.143323996	0.598	0.525	0.0730
6.144184581	0.598	0.525	0.0730
6.144914855	0.598	0.525	0.0730
6.146699772	0.598	0.525	0.0730
6.234931009	0.58	0.507	0.0730
6.235624587	0.579	0.506	0.0730
6.237977528	0.579	0.506	0.0730
6.240300752	0.579	0.506	0.0730
6.240868128	0.579	0.506	0.0730
6.242231229	0.578	0.505	0.0730
5.309493401	0.763	0.691	0.0720
5.310539645	0.762	0.69	0.0720
5.311617232	0.762	0.69	0.0720
5.313254831	0.762	0.69	0.0720
5.314147688	0.762	0.69	0.0720
5.318778307	0.761	0.689	0.0720
5.321840781	0.761	0.689	0.0720
5.348029716	0.756	0.684	0.0720
5.34957784	0.756	0.684	0.0720
5.351215574	0.756	0.684	0.0720
5.353238265	0.755	0.683	0.0720

5.353933031	0.755	0.683	0.0720
5.354584709	0.755	0.683	0.0720
5.355014983	0.755	0.683	0.0720
5.355320747	0.755	0.683	0.0720
6.148398398	0.597	0.525	0.0720
6.148858486	0.597	0.525	0.0720
6.163156355	0.594	0.522	0.0720
6.163952787	0.594	0.522	0.0720
6.168992699	0.593	0.521	0.0720
6.169683497	0.593	0.521	0.0720
6.171651786	0.592	0.52	0.0720
6.172357253	0.592	0.52	0.0720
6.173668075	0.591	0.519	0.0720
6.175483616	0.591	0.519	0.0720
6.191829004	0.588	0.516	0.0720
6.193365103	0.588	0.516	0.0720
6.220221214	0.583	0.511	0.0720
6.221576227	0.583	0.511	0.0720
6.222999223	0.583	0.511	0.0720
6.224470893	0.583	0.511	0.0720
6.229039813	0.582	0.51	0.0720
6.233321473	0.58	0.508	0.0720
6.234167068	0.58	0.508	0.0720
6.241372745	0.578	0.506	0.0720
6.241824477	0.578	0.506	0.0720
6.242599404	0.577	0.505	0.0720
6.243080405	0.577	0.505	0.0720
6.246693122	0.577	0.505	0.0720
5.306692509	0.763	0.692	0.0710
5.307952273	0.763	0.692	0.0710
6.597930839	0.502	0.431	0.0710
6.605499439	0.5	0.429	0.0710
6.149246805	0.596	0.525	0.0710
6.151635721	0.596	0.525	0.0710
6.153846154	0.596	0.525	0.0710
6.155870445	0.595	0.524	0.0710
6.158215661	0.595	0.524	0.0710
6.158738491	0.595	0.524	0.0710
6.160791226	0.594	0.523	0.0710
6.16247643	0.594	0.523	0.0710
6.164898548	0.593	0.522	0.0710
6.1660401	0.593	0.522	0.0710
6.167748918	0.593	0.522	0.0710

6.170820669	0.592	0.521	0.0710
6.17724053	0.59	0.519	0.0710
6.17817721	0.59	0.519	0.0710
6.179059615	0.59	0.519	0.0710
6.182512144	0.589	0.518	0.0710
6.183708317	0.589	0.518	0.0710
6.184590812	0.589	0.518	0.0710
6.193789119	0.587	0.516	0.0710
6.194841012	0.587	0.516	0.0710
6.195985596	0.587	0.516	0.0710
6.196751058	0.587	0.516	0.0710
6.198006754	0.587	0.516	0.0710
6.199973864	0.586	0.515	0.0710
6.201333853	0.586	0.515	0.0710
6.202379472	0.586	0.515	0.0710
6.203794059	0.586	0.515	0.0710
6.210165226	0.585	0.514	0.0710
6.211650668	0.585	0.514	0.0710
6.217082467	0.584	0.513	0.0710
6.218730457	0.583	0.512	0.0710
6.225790329	0.582	0.511	0.0710
6.227493261	0.582	0.511	0.0710
6.229508197	0.581	0.51	0.0710
6.230138714	0.581	0.51	0.0710
6.231326644	0.581	0.51	0.0710
6.232380385	0.581	0.51	0.0710
6.253078818	0.575	0.504	0.0710
5.297655271	0.764	0.694	0.0700
5.299279847	0.764	0.694	0.0700
5.300875634	0.764	0.694	0.0700
5.301609121	0.763	0.693	0.0700
5.302940413	0.763	0.693	0.0700
5.304372726	0.763	0.693	0.0700
5.305500249	0.763	0.693	0.0700
5.306122449	0.763	0.693	0.0700
6.58869129	0.504	0.434	0.0700
6.593804746	0.503	0.433	0.0700
6.59497379	0.503	0.433	0.0700
6.596483452	0.502	0.432	0.0700
6.599319728	0.501	0.431	0.0700
6.600653595	0.501	0.431	0.0700
6.601935646	0.5	0.43	0.0700
6.603168844	0.5	0.43	0.0700

6.604355928	0.5	0.43	0.0700
6.606601732	0.499	0.429	0.0700
6.60816913	0.499	0.429	0.0700
6.610382825	0.499	0.429	0.0700
6.612236737	0.499	0.429	0.0700
6.61332992	0.499	0.429	0.0700
6.615701836	0.499	0.429	0.0700
6.618176047	0.498	0.428	0.0700
6.635217391	0.495	0.425	0.0700
6.642063406	0.494	0.424	0.0700
6.642596455	0.494	0.424	0.0700
6.70695195	0.482	0.412	0.0700
6.159180344	0.594	0.524	0.0700
6.180165418	0.589	0.519	0.0700
6.181186869	0.589	0.519	0.0700
6.186010729	0.588	0.518	0.0700
6.18727518	0.588	0.518	0.0700
6.188988095	0.588	0.518	0.0700
6.19047619	0.588	0.518	0.0700
6.205638057	0.585	0.515	0.0700
6.206896552	0.585	0.515	0.0700
6.20784388	0.585	0.515	0.0700
6.208970768	0.585	0.515	0.0700
6.212569482	0.584	0.514	0.0700
6.213651733	0.584	0.514	0.0700
6.215250965	0.584	0.514	0.0700
6.256570438	0.574	0.504	0.0700
6.257146591	0.574	0.504	0.0700
6.257489327	0.574	0.504	0.0700
6.257866614	0.574	0.504	0.0700
6.258283959	0.574	0.504	0.0700
6.259267395	0.573	0.503	0.0700
6.264714029	0.572	0.502	0.0700
6.265441513	0.572	0.502	0.0700
6.266244726	0.572	0.502	0.0700
6.26713615	0.571	0.501	0.0700
6.281269543	0.569	0.499	0.0700
6.295206972	0.566	0.496	0.0700
5.290510128	0.765	0.696	0.0690
5.290851483	0.765	0.696	0.0690
5.295707472	0.764	0.695	0.0690
6.564651337	0.509	0.44	0.0690
6.587063967	0.504	0.435	0.0690

6.587768441	0.504	0.435	0.0690
6.590028189	0.503	0.434	0.0690
6.591750842	0.503	0.434	0.0690
6.592999593	0.503	0.434	0.0690
6.618876328	0.497	0.428	0.0690
6.619382964	0.497	0.428	0.0690
6.620203983	0.497	0.428	0.0690
6.621155638	0.496	0.427	0.0690
6.622069089	0.496	0.427	0.0690
6.62294659	0.496	0.427	0.0690
6.623790223	0.496	0.427	0.0690
6.627562956	0.495	0.426	0.0690
6.628239203	0.495	0.426	0.0690
6.628892456	0.495	0.426	0.0690
6.629824133	0.495	0.426	0.0690
6.6403125	0.494	0.425	0.0690
6.640923187	0.494	0.425	0.0690
6.641506209	0.494	0.425	0.0690
6.643106893	0.493	0.424	0.0690
6.657032755	0.49	0.421	0.0690
6.666666667	0.49	0.421	0.0690
6.687905844	0.484	0.415	0.0690
6.688526705	0.484	0.415	0.0690
6.688965456	0.484	0.415	0.0690
6.689422181	0.484	0.415	0.0690
6.690911976	0.483	0.414	0.0690
6.691452897	0.483	0.414	0.0690
6.704228035	0.482	0.413	0.0690
6.705213904	0.482	0.413	0.0690
6.70623459	0.482	0.413	0.0690
6.707695804	0.481	0.412	0.0690
6.708467647	0.481	0.412	0.0690
6.709269089	0.481	0.412	0.0690
6.710101868	0.481	0.412	0.0690
6.71280774	0.48	0.411	0.0690
6.713786214	0.479	0.41	0.0690
6.716987581	0.478	0.409	0.0690
6.718153626	0.478	0.409	0.0690
6.728977405	0.477	0.408	0.0690
6.258748104	0.573	0.504	0.0690
6.259852293	0.572	0.503	0.0690
6.262265209	0.572	0.503	0.0690
6.268131175	0.57	0.501	0.0690

6.270423033	0.57	0.501	0.0690
6.272740637	0.57	0.501	0.0690
6.279778786	0.569	0.5	0.0690
6.282917533	0.568	0.499	0.0690
6.284749035	0.568	0.499	0.0690
6.289815378	0.567	0.498	0.0690
6.290856655	0.567	0.498	0.0690
6.29391197	0.566	0.497	0.0690
6.297498797	0.565	0.496	0.0690
6.298956949	0.565	0.496	0.0690
6.299895317	0.565	0.496	0.0690
6.300973949	0.565	0.496	0.0690
6.303173919	0.564	0.495	0.0690
6.303832681	0.564	0.495	0.0690
6.307255245	0.563	0.494	0.0690
6.30820857	0.563	0.494	0.0690
5.287657219	0.765	0.697	0.0680
5.288906594	0.765	0.697	0.0680
5.290088827	0.765	0.697	0.0680
5.292561469	0.764	0.696	0.0680
5.294117647	0.764	0.696	0.0680
6.309124321	0.562	0.494	0.0680
6.309842119	0.562	0.494	0.0680
6.311108583	0.562	0.494	0.0680
6.313923106	0.562	0.494	0.0680
6.342724868	0.557	0.489	0.0680
6.348255869	0.556	0.488	0.0680
6.349785656	0.556	0.488	0.0680
6.355042017	0.554	0.486	0.0680
6.516491482	0.518	0.45	0.0680
6.517686226	0.518	0.45	0.0680
6.51875293	0.518	0.45	0.0680
6.550509956	0.512	0.444	0.0680
6.552889096	0.511	0.443	0.0680
6.554337951	0.51	0.442	0.0680
6.556531704	0.51	0.442	0.0680
6.561504777	0.509	0.441	0.0680
6.563458589	0.509	0.441	0.0680
6.565575278	0.508	0.44	0.0680
6.56671462	0.508	0.44	0.0680
6.570598007	0.507	0.439	0.0680
6.575959521	0.506	0.438	0.0680
6.585338639	0.504	0.436	0.0680

6.624601911	0.495	0.427	0.0680
6.625383436	0.495	0.427	0.0680
6.626492903	0.495	0.427	0.0680
6.64359613	0.492	0.424	0.0680
6.644065459	0.492	0.424	0.0680
6.646882705	0.49	0.422	0.0680
6.647228834	0.49	0.422	0.0680
6.6765286	0.485	0.417	0.0680
6.68656876	0.485	0.417	0.0680
6.686931776	0.485	0.417	0.0680
6.690151327	0.483	0.415	0.0680
6.692018508	0.482	0.414	0.0680
6.69292804	0.482	0.414	0.0680
6.698729501	0.482	0.414	0.0680
6.710967856	0.48	0.412	0.0680
6.711869082	0.48	0.412	0.0680
6.714807091	0.478	0.41	0.0680
6.715873189	0.478	0.41	0.0680
6.721443966	0.477	0.409	0.0680
6.725705329	0.477	0.409	0.0680
6.727833895	0.477	0.409	0.0680
6.73016449	0.476	0.408	0.0680
6.731397687	0.476	0.408	0.0680
6.734013605	0.475	0.407	0.0680
6.773172724	0.466	0.398	0.0680
6.774193548	0.466	0.398	0.0680
6.273900865	0.569	0.501	0.0680
6.275185936	0.569	0.501	0.0680
6.276617166	0.569	0.501	0.0680
6.278221015	0.569	0.501	0.0680
6.286796537	0.567	0.499	0.0680
6.288593482	0.567	0.499	0.0680
6.292548511	0.566	0.498	0.0680
6.301726598	0.564	0.496	0.0680
6.302556818	0.564	0.496	0.0680
6.305040155	0.563	0.495	0.0680
6.306275333	0.563	0.495	0.0680
6.736849882	0.474	0.406	0.0680
6.743305144	0.472	0.404	0.0680
6.744186047	0.472	0.404	0.0680
5.278482798	0.767	0.7	0.0670
5.279345461	0.767	0.7	0.0670
5.281294509	0.766	0.699	0.0670

5.281870711	0.766	0.699	0.0670
5.283071096	0.766	0.699	0.0670
5.286990263	0.765	0.698	0.0670
6.315789474	0.561	0.494	0.0670
6.317403939	0.561	0.494	0.0670
6.319231425	0.561	0.494	0.0670
6.319722222	0.561	0.494	0.0670
6.324555629	0.56	0.493	0.0670
6.327327806	0.56	0.493	0.0670
6.328619462	0.56	0.493	0.0670
6.330237435	0.559	0.492	0.0670
6.33234714	0.559	0.492	0.0670
6.338412992	0.558	0.491	0.0670
6.342028004	0.557	0.49	0.0670
6.343070362	0.556	0.489	0.0670
6.344055584	0.556	0.489	0.0670
6.345490716	0.556	0.489	0.0670
6.346729618	0.556	0.489	0.0670
6.350858157	0.555	0.488	0.0670
6.357379376	0.553	0.486	0.0670
6.47943662	0.526	0.459	0.0670
6.480223464	0.526	0.459	0.0670
6.480964204	0.526	0.459	0.0670
6.487290862	0.524	0.457	0.0670
6.488322428	0.524	0.457	0.0670
6.48895566	0.524	0.457	0.0670
6.489713964	0.524	0.457	0.0670
6.501533742	0.521	0.454	0.0670
6.503281994	0.521	0.454	0.0670
6.506436706	0.52	0.453	0.0670
6.507392912	0.519	0.452	0.0670
6.509001811	0.519	0.452	0.0670
6.510847511	0.519	0.452	0.0670
6.512392901	0.519	0.452	0.0670
6.513721805	0.518	0.451	0.0670
6.514718615	0.518	0.451	0.0670
6.515640274	0.518	0.451	0.0670
6.519162179	0.517	0.45	0.0670
6.520538074	0.517	0.45	0.0670
6.528178936	0.516	0.449	0.0670
6.530012005	0.516	0.449	0.0670
6.531202076	0.515	0.448	0.0670
6.53256262	0.515	0.448	0.0670

6.534640523	0.515	0.448	0.0670
6.536130281	0.514	0.447	0.0670
6.537387194	0.514	0.447	0.0670
6.545440118	0.512	0.445	0.0670
6.547190819	0.512	0.445	0.0670
6.548457411	0.512	0.445	0.0670
6.558790672	0.509	0.442	0.0670
6.55982467	0.509	0.442	0.0670
6.567605758	0.507	0.44	0.0670
6.56890739	0.507	0.44	0.0670
6.57223114	0.506	0.439	0.0670
6.573230141	0.506	0.439	0.0670
6.57438452	0.506	0.439	0.0670
6.576878892	0.505	0.438	0.0670
6.578064288	0.505	0.438	0.0670
6.579796265	0.505	0.438	0.0670
6.581461821	0.504	0.437	0.0670
6.583064706	0.504	0.437	0.0670
6.644516072	0.491	0.424	0.0670
6.644949066	0.491	0.424	0.0670
6.645365455	0.491	0.424	0.0670
6.645955542	0.491	0.424	0.0670
6.687308282	0.484	0.417	0.0670
6.732679739	0.475	0.408	0.0670
6.735402494	0.474	0.407	0.0670
6.768074031	0.467	0.4	0.0670
6.775254669	0.465	0.398	0.0670
6.77741964	0.465	0.398	0.0670
6.779092253	0.465	0.398	0.0670
6.779661017	0.465	0.398	0.0670
6.987468867	0.425	0.358	0.0670
6.990864257	0.424	0.357	0.0670
6.992744098	0.424	0.357	0.0670
6.993235523	0.424	0.357	0.0670
6.993664541	0.423	0.356	0.0670
6.994042342	0.423	0.356	0.0670
6.997109827	0.423	0.356	0.0670
7.006178725	0.421	0.354	0.0670
7.006586074	0.421	0.354	0.0670
7.007050996	0.421	0.354	0.0670
7.007586643	0.421	0.354	0.0670
7.010034569	0.421	0.354	0.0670
7.013042005	0.421	0.354	0.0670

5.029243766	0.817	0.75	0.0670
6.738359544	0.473	0.406	0.0670
6.739935588	0.473	0.406	0.0670
6.741156887	0.472	0.405	0.0670
6.741998638	0.472	0.405	0.0670
6.745108896	0.471	0.404	0.0670
6.746509849	0.471	0.404	0.0670
6.747477716	0.471	0.404	0.0670
6.74898374	0.471	0.404	0.0670
6.750796178	0.471	0.404	0.0670
6.753279622	0.471	0.404	0.0670
6.755143018	0.471	0.404	0.0670
6.757688723	0.47	0.403	0.0670
6.759592035	0.469	0.402	0.0670
6.760963508	0.469	0.402	0.0670
6.761976782	0.469	0.402	0.0670
5.188089623	0.784	0.718	0.0660
5.188934217	0.784	0.718	0.0660
5.189531303	0.784	0.718	0.0660
5.190356556	0.784	0.718	0.0660
5.191782261	0.783	0.717	0.0660
5.192838929	0.783	0.717	0.0660
5.19408768	0.783	0.717	0.0660
5.203690533	0.782	0.716	0.0660
5.207716963	0.78	0.714	0.0660
5.208333333	0.78	0.714	0.0660
5.208817829	0.78	0.714	0.0660
5.209441582	0.78	0.714	0.0660
5.213525836	0.779	0.713	0.0660
5.21450482	0.779	0.713	0.0660
5.280200991	0.766	0.7	0.0660
5.285357556	0.765	0.699	0.0660
6.320621762	0.56	0.494	0.0660
6.321541302	0.56	0.494	0.0660
6.322209863	0.56	0.494	0.0660
6.33436853	0.558	0.492	0.0660
6.335640795	0.558	0.492	0.0660
6.336543582	0.558	0.492	0.0660
6.337618736	0.558	0.492	0.0660
6.339333548	0.557	0.491	0.0660
6.340666348	0.557	0.491	0.0660
6.351776305	0.554	0.488	0.0660
6.352571217	0.554	0.488	0.0660

6.357820293	0.552	0.486	0.0660
6.358203097	0.552	0.486	0.0660
6.361008933	0.552	0.486	0.0660
6.363636364	0.552	0.486	0.0660
6.366028708	0.55	0.484	0.0660
6.368734336	0.55	0.484	0.0660
6.369644883	0.548	0.482	0.0660
6.370116692	0.548	0.482	0.0660
6.370669056	0.548	0.482	0.0660
6.375905797	0.547	0.481	0.0660
6.377382175	0.547	0.481	0.0660
6.37863155	0.547	0.481	0.0660
6.474589559	0.528	0.462	0.0660
6.47511499	0.527	0.461	0.0660
6.476341282	0.527	0.461	0.0660
6.477630074	0.527	0.461	0.0660
6.47843033	0.526	0.46	0.0660
6.482120051	0.525	0.459	0.0660
6.48356681	0.525	0.459	0.0660
6.484611742	0.525	0.459	0.0660
6.491106411	0.523	0.457	0.0660
6.492341955	0.523	0.457	0.0660
6.493021903	0.523	0.457	0.0660
6.494928429	0.523	0.457	0.0660
6.496995214	0.522	0.456	0.0660
6.498587571	0.522	0.456	0.0660
6.5047603	0.52	0.454	0.0660
6.523064687	0.516	0.45	0.0660
6.524606469	0.516	0.45	0.0660
6.525884401	0.516	0.45	0.0660
6.53950104	0.513	0.447	0.0660
6.540710522	0.513	0.447	0.0660
6.541116943	0.513	0.447	0.0660
6.54228163	0.512	0.446	0.0660
6.543663762	0.512	0.446	0.0660
6.646524025	0.49	0.424	0.0660
6.762797854	0.468	0.402	0.0660
6.763855831	0.468	0.402	0.0660
6.765586474	0.467	0.401	0.0660
6.76669218	0.467	0.401	0.0660
6.769708552	0.466	0.4	0.0660
6.770919939	0.466	0.4	0.0660
6.771902721	0.466	0.4	0.0660

6.780241467	0.464	0.398	0.0660
6.782019351	0.463	0.397	0.0660
6.783421257	0.463	0.397	0.0660
6.784670008	0.463	0.397	0.0660
6.786796537	0.463	0.397	0.0660
6.788099978	0.463	0.397	0.0660
6.790584116	0.462	0.396	0.0660
6.795225953	0.461	0.395	0.0660
6.796227555	0.461	0.395	0.0660
6.798876404	0.46	0.394	0.0660
6.801360544	0.459	0.393	0.0660
6.803727408	0.459	0.393	0.0660
6.805144642	0.459	0.393	0.0660
6.807869777	0.458	0.392	0.0660
6.809163295	0.458	0.392	0.0660
6.810313381	0.458	0.392	0.0660
6.811202507	0.458	0.392	0.0660
6.813204509	0.458	0.392	0.0660
6.816734221	0.457	0.391	0.0660
6.818181818	0.457	0.391	0.0660
6.868849444	0.446	0.38	0.0660
6.870488653	0.446	0.38	0.0660
6.87287415	0.446	0.38	0.0660
6.985797744	0.425	0.359	0.0660
6.988941838	0.424	0.358	0.0660
7.002824859	0.421	0.355	0.0660
7.005818871	0.421	0.355	0.0660
7.014407131	0.42	0.354	0.0660
7.015527203	0.42	0.354	0.0660
5.025790327	0.817	0.751	0.0660
5.026596497	0.817	0.751	0.0660
5.027181139	0.817	0.751	0.0660
5.027778635	0.817	0.751	0.0660
5.028090774	0.817	0.751	0.0660
5.028575161	0.817	0.751	0.0660
5.029762959	0.816	0.75	0.0660
5.030121575	0.816	0.75	0.0660
5.030679465	0.816	0.75	0.0660
5.031251221	0.816	0.75	0.0660
5.050252525	0.813	0.747	0.0660
5.050762729	0.813	0.747	0.0660
6.756037953	0.47	0.404	0.0660
6.756756757	0.47	0.404	0.0660

5.051825994	0.812	0.747	0.0650
5.052911534	0.812	0.747	0.0650
5.054068272	0.812	0.747	0.0650
5.055250305	0.812	0.747	0.0650
5.055867665	0.812	0.747	0.0650
5.059173669	0.811	0.746	0.0650
5.06024971	0.811	0.746	0.0650
5.06289557	0.81	0.745	0.0650
5.085230024	0.806	0.741	0.0650
5.085960591	0.806	0.741	0.0650
5.090364725	0.805	0.74	0.0650
5.091466815	0.805	0.74	0.0650
5.092880147	0.804	0.739	0.0650
5.093753662	0.804	0.739	0.0650
5.096157797	0.803	0.738	0.0650
5.098688482	0.802	0.737	0.0650
5.101355979	0.801	0.736	0.0650
5.102744546	0.801	0.736	0.0650
5.103807471	0.801	0.736	0.0650
5.104530886	0.801	0.736	0.0650
5.106382979	0.8	0.735	0.0650
5.107148324	0.8	0.735	0.0650
5.108304661	0.8	0.735	0.0650
5.133511586	0.796	0.731	0.0650
5.134412487	0.796	0.731	0.0650
5.180018148	0.787	0.722	0.0650
5.182704291	0.786	0.721	0.0650
5.184215629	0.785	0.72	0.0650
5.185185185	0.785	0.72	0.0650
5.186342593	0.784	0.719	0.0650
5.191048263	0.783	0.718	0.0650
5.195827794	0.782	0.717	0.0650
5.197295253	0.782	0.717	0.0650
5.198870056	0.782	0.717	0.0650
5.201156069	0.782	0.717	0.0650
5.202507421	0.782	0.717	0.0650
5.205078907	0.781	0.716	0.0650
5.206856081	0.78	0.715	0.0650
5.210053577	0.779	0.714	0.0650
5.210896961	0.779	0.714	0.0650
5.212016782	0.779	0.714	0.0650
5.214888845	0.778	0.713	0.0650
5.216222534	0.778	0.713	0.0650

5.217391304	0.778	0.713	0.0650
5.219953003	0.777	0.712	0.0650
5.220357011	0.777	0.712	0.0650
5.221759086	0.777	0.712	0.0650
5.223824519	0.777	0.712	0.0650
5.225262776	0.776	0.711	0.0650
5.226539589	0.776	0.711	0.0650
5.227272727	0.776	0.711	0.0650
5.228015449	0.776	0.711	0.0650
5.230327144	0.775	0.71	0.0650
5.231278655	0.775	0.71	0.0650
5.23217311	0.775	0.71	0.0650
5.264715042	0.769	0.704	0.0650
5.266469428	0.769	0.704	0.0650
5.2710047	0.768	0.703	0.0650
5.272233202	0.768	0.703	0.0650
5.273349938	0.768	0.703	0.0650
5.274348939	0.768	0.703	0.0650
5.274977316	0.767	0.702	0.0650
5.275651489	0.767	0.702	0.0650
5.276227765	0.767	0.702	0.0650
5.277079844	0.767	0.702	0.0650
6.369237185	0.549	0.484	0.0650
6.372618374	0.547	0.482	0.0650
6.374634503	0.547	0.482	0.0650
6.379839221	0.546	0.481	0.0650
6.380973523	0.546	0.481	0.0650
6.382278835	0.546	0.481	0.0650
6.468827052	0.529	0.464	0.0650
6.485667486	0.524	0.459	0.0650
6.496582826	0.522	0.457	0.0650
6.789222312	0.462	0.397	0.0650
6.792468953	0.461	0.396	0.0650
6.794382462	0.461	0.396	0.0650
6.79713031	0.46	0.395	0.0650
6.797569215	0.46	0.395	0.0650
6.806392236	0.458	0.393	0.0650
6.81505072	0.457	0.392	0.0650
6.819686936	0.456	0.391	0.0650
6.822360732	0.456	0.391	0.0650
6.823926868	0.456	0.391	0.0650
6.824860575	0.455	0.39	0.0650
6.826491516	0.455	0.39	0.0650

6.827771598	0.455	0.39	0.0650
6.828612641	0.455	0.39	0.0650
6.866169154	0.446	0.381	0.0650
6.867068273	0.446	0.381	0.0650
6.876806358	0.445	0.38	0.0650
6.879716312	0.444	0.379	0.0650
6.881558442	0.444	0.379	0.0650
6.883587427	0.444	0.379	0.0650
6.884651936	0.443	0.378	0.0650
6.885736723	0.443	0.378	0.0650
6.886822382	0.443	0.378	0.0650
6.888153054	0.443	0.378	0.0650
6.951545585	0.431	0.366	0.0650
6.957793033	0.43	0.365	0.0650
6.959261893	0.43	0.365	0.0650
6.96249414	0.429	0.364	0.0650
6.964240102	0.429	0.364	0.0650
6.966629588	0.429	0.364	0.0650
6.980498924	0.426	0.361	0.0650
6.982744372	0.425	0.36	0.0650
6.983683604	0.425	0.36	0.0650
6.984525804	0.425	0.36	0.0650
6.98510937	0.425	0.36	0.0650
7.017381286	0.419	0.354	0.0650
7.019250545	0.419	0.354	0.0650
7.020572073	0.419	0.354	0.0650
7.02208868	0.419	0.354	0.0650
7.023847076	0.419	0.354	0.0650
7.044739851	0.415	0.35	0.0650
7.045454545	0.415	0.35	0.0650
5.024524537	0.817	0.752	0.0650
5.031646837	0.815	0.75	0.0650
5.032052599	0.815	0.75	0.0650
5.033334815	0.814	0.749	0.0650
5.033785326	0.814	0.749	0.0650
5.034248182	0.814	0.749	0.0650
5.049509804	0.813	0.748	0.0650
5.056498979	0.811	0.747	0.0640
5.057478858	0.811	0.747	0.0640
5.058481532	0.811	0.747	0.0640
5.061352002	0.81	0.746	0.0640
5.062114198	0.81	0.746	0.0640
5.063696852	0.809	0.745	0.0640

5.064518815	0.809	0.745	0.0640
5.065362269	0.809	0.745	0.0640
5.06622807	0.809	0.745	0.0640
5.084272323	0.806	0.742	0.0640
5.087482147	0.805	0.741	0.0640
5.089288878	0.805	0.741	0.0640
5.092308566	0.804	0.74	0.0640
5.094940512	0.803	0.739	0.0640
5.096930786	0.802	0.738	0.0640
5.097563297	0.802	0.738	0.0640
5.098039216	0.802	0.738	0.0640
5.100004445	0.801	0.737	0.0640
5.105639042	0.8	0.736	0.0640
5.109292881	0.799	0.735	0.0640
5.110500611	0.799	0.735	0.0640
5.111735331	0.799	0.735	0.0640
5.112997957	0.799	0.735	0.0640
5.1312345	0.796	0.732	0.0640
5.13245614	0.796	0.732	0.0640
5.135873578	0.795	0.731	0.0640
5.136799162	0.795	0.731	0.0640
5.138505218	0.794	0.73	0.0640
5.139276847	0.794	0.73	0.0640
5.140254937	0.794	0.73	0.0640
5.141044004	0.794	0.73	0.0640
5.14205004	0.794	0.73	0.0640
5.1621669	0.79	0.726	0.0640
5.164303196	0.79	0.726	0.0640
5.16611479	0.79	0.726	0.0640
5.167225951	0.79	0.726	0.0640
5.173416199	0.788	0.724	0.0640
5.17462189	0.788	0.724	0.0640
5.175352286	0.788	0.724	0.0640
5.176887776	0.787	0.723	0.0640
5.177938197	0.787	0.723	0.0640
5.179213772	0.787	0.723	0.0640
5.180451536	0.786	0.722	0.0640
5.181035021	0.786	0.722	0.0640
5.181914451	0.786	0.722	0.0640
5.183086451	0.785	0.721	0.0640
5.206045511	0.78	0.716	0.0640
5.21845175	0.777	0.713	0.0640
5.219646207	0.777	0.713	0.0640

5.229321614	0.775	0.711	0.0640
5.233728734	0.774	0.71	0.0640
5.235096723	0.774	0.71	0.0640
5.236694678	0.774	0.71	0.0640
5.24640761	0.773	0.709	0.0640
5.255756149	0.771	0.707	0.0640
5.256776557	0.771	0.707	0.0640
5.258201058	0.77	0.706	0.0640
5.259499759	0.77	0.706	0.0640
5.259927933	0.77	0.706	0.0640
5.260266137	0.77	0.706	0.0640
5.263157895	0.769	0.705	0.0640
5.267741935	0.768	0.704	0.0640
5.269139141	0.768	0.704	0.0640
5.269865674	0.768	0.704	0.0640
6.383797054	0.545	0.481	0.0640
6.385078777	0.545	0.481	0.0640
6.386319471	0.545	0.481	0.0640
6.388888889	0.544	0.48	0.0640
6.391691253	0.543	0.479	0.0640
6.39495333	0.542	0.478	0.0640
6.39620383	0.542	0.478	0.0640
6.465126906	0.529	0.465	0.0640
6.46641604	0.529	0.465	0.0640
6.466866267	0.529	0.465	0.0640
6.472473605	0.528	0.464	0.0640
6.830127104	0.454	0.39	0.0640
6.843671679	0.451	0.387	0.0640
6.84543784	0.451	0.387	0.0640
6.846731835	0.451	0.387	0.0640
6.849854385	0.45	0.386	0.0640
6.851122776	0.449	0.385	0.0640
6.852499352	0.449	0.385	0.0640
6.853992781	0.449	0.385	0.0640
6.855092311	0.449	0.385	0.0640
6.858058608	0.448	0.384	0.0640
6.859719738	0.447	0.383	0.0640
6.860889492	0.447	0.383	0.0640
6.861507998	0.447	0.383	0.0640
6.862223613	0.447	0.383	0.0640
6.863325212	0.446	0.382	0.0640
6.864788484	0.446	0.382	0.0640
6.878796804	0.444	0.38	0.0640

6.879206758	0.444	0.38	0.0640
6.889566396	0.442	0.378	0.0640
6.890717806	0.442	0.378	0.0640
6.891541801	0.442	0.378	0.0640
6.897642951	0.441	0.377	0.0640
6.903993856	0.439	0.375	0.0640
6.904761905	0.439	0.375	0.0640
6.905618362	0.439	0.375	0.0640
6.907184778	0.439	0.375	0.0640
6.908053649	0.438	0.374	0.0640
6.908651735	0.438	0.374	0.0640
6.909830007	0.438	0.374	0.0640
6.911166906	0.438	0.374	0.0640
6.923076923	0.436	0.372	0.0640
6.925225612	0.435	0.371	0.0640
6.927739438	0.435	0.371	0.0640
6.928852643	0.434	0.37	0.0640
6.930824598	0.434	0.37	0.0640
6.940737489	0.432	0.368	0.0640
6.941925815	0.432	0.368	0.0640
6.943559802	0.432	0.368	0.0640
6.945276114	0.432	0.368	0.0640
6.946336335	0.432	0.368	0.0640
6.950787061	0.431	0.367	0.0640
6.952757021	0.43	0.366	0.0640
6.955082062	0.43	0.366	0.0640
6.960742388	0.429	0.365	0.0640
6.968719453	0.428	0.364	0.0640
6.970763978	0.428	0.364	0.0640
6.974496426	0.427	0.363	0.0640
6.976026414	0.427	0.363	0.0640
6.977580726	0.427	0.363	0.0640
6.979141519	0.426	0.362	0.0640
6.981690298	0.425	0.361	0.0640
7.025054922	0.418	0.354	0.0640
7.026171741	0.418	0.354	0.0640
7.030621786	0.417	0.353	0.0640
7.033370412	0.417	0.353	0.0640
7.035960379	0.416	0.352	0.0640
7.038255361	0.416	0.352	0.0640
7.039736842	0.415	0.351	0.0640
7.040408163	0.415	0.351	0.0640
7.041534924	0.415	0.351	0.0640

7.043139339	0.415	0.351	0.0640
7.046217206	0.414	0.35	0.0640
7.048669789	0.414	0.35	0.0640
7.051652586	0.413	0.349	0.0640
7.053143344	0.413	0.349	0.0640
7.054529043	0.412	0.348	0.0640
7.055004622	0.412	0.348	0.0640
7.211999554	0.381	0.317	0.0640
7.213700234	0.381	0.317	0.0640
7.21829408	0.38	0.316	0.0640
7.218738979	0.38	0.316	0.0640
4.969511062	0.826	0.762	0.0640
4.970410057	0.825	0.761	0.0640
4.97092925	0.825	0.761	0.0640
5.011961722	0.817	0.753	0.0640
5.032468902	0.814	0.75	0.0640
5.032896161	0.814	0.75	0.0640
5.034723897	0.813	0.749	0.0640
5.036566487	0.813	0.749	0.0640
5.042019749	0.813	0.749	0.0640
5.046974241	0.813	0.749	0.0640
5.048548265	0.813	0.749	0.0640
5.067117117	0.808	0.745	0.0630
5.068030359	0.808	0.745	0.0630
5.068808787	0.808	0.745	0.0630
5.069284434	0.808	0.745	0.0630
5.083335905	0.806	0.743	0.0630
5.114289446	0.798	0.735	0.0630
5.115074951	0.798	0.735	0.0630
5.115743222	0.798	0.735	0.0630
5.116963064	0.798	0.735	0.0630
5.119064493	0.798	0.735	0.0630
5.123711866	0.797	0.734	0.0630
5.124172526	0.797	0.734	0.0630
5.124689055	0.797	0.734	0.0630
5.130380091	0.796	0.733	0.0630
5.137553924	0.794	0.731	0.0630
5.143682907	0.793	0.73	0.0630
5.144718103	0.793	0.73	0.0630
5.145563183	0.793	0.73	0.0630
5.146628827	0.793	0.73	0.0630
5.147493909	0.793	0.73	0.0630
5.154959339	0.792	0.729	0.0630

5.160432395	0.79	0.727	0.0630
5.16816228	0.789	0.726	0.0630
5.169303677	0.789	0.726	0.0630
5.170261286	0.789	0.726	0.0630
5.171434169	0.789	0.726	0.0630
5.176174993	0.787	0.724	0.0630
5.239368475	0.773	0.71	0.0630
5.241010511	0.773	0.71	0.0630
5.242311277	0.773	0.71	0.0630
5.243572841	0.773	0.71	0.0630
5.244902039	0.773	0.71	0.0630
5.247570265	0.772	0.709	0.0630
5.248422867	0.772	0.709	0.0630
5.249309392	0.772	0.709	0.0630
5.250698324	0.772	0.709	0.0630
5.254669664	0.771	0.708	0.0630
5.261787281	0.769	0.706	0.0630
6.387992832	0.544	0.481	0.0630
6.389710717	0.543	0.48	0.0630
6.390754994	0.543	0.48	0.0630
6.393481443	0.542	0.479	0.0630
6.397287176	0.541	0.478	0.0630
6.397682495	0.541	0.478	0.0630
6.398924731	0.541	0.478	0.0630
6.460150743	0.53	0.467	0.0630
6.461629596	0.53	0.467	0.0630
6.462999834	0.529	0.466	0.0630
6.463751516	0.529	0.466	0.0630
6.831642026	0.453	0.39	0.0630
6.832815735	0.453	0.39	0.0630
6.833932854	0.453	0.39	0.0630
6.834987706	0.453	0.39	0.0630
6.836839166	0.453	0.39	0.0630
6.84040747	0.452	0.389	0.0630
6.842105263	0.452	0.389	0.0630
6.848155468	0.45	0.387	0.0630
6.848899958	0.45	0.387	0.0630
6.855508008	0.448	0.385	0.0630
6.85640648	0.448	0.385	0.0630
6.89227363	0.441	0.378	0.0630
6.893297381	0.441	0.378	0.0630
6.894174666	0.441	0.378	0.0630
6.895480831	0.441	0.378	0.0630

6.896551724	0.441	0.378	0.0630
6.898979492	0.44	0.377	0.0630
6.899904801	0.44	0.377	0.0630
6.900996623	0.44	0.377	0.0630
6.902317129	0.44	0.377	0.0630
6.912258192	0.437	0.374	0.0630
6.913518696	0.437	0.374	0.0630
6.918421822	0.436	0.373	0.0630
6.920840787	0.436	0.373	0.0630
6.928338002	0.434	0.371	0.0630
6.932924335	0.433	0.37	0.0630
6.933819951	0.433	0.37	0.0630
6.93489522	0.433	0.37	0.0630
6.936491935	0.433	0.37	0.0630
6.939537009	0.432	0.369	0.0630
6.947308417	0.431	0.368	0.0630
6.949203279	0.431	0.368	0.0630
6.972154025	0.427	0.364	0.0630
6.972725019	0.427	0.364	0.0630
6.973328592	0.427	0.364	0.0630
7.027799228	0.417	0.354	0.0630
7.028778468	0.417	0.354	0.0630
7.03468324	0.416	0.353	0.0630
7.050820882	0.413	0.35	0.0630
7.05538514	0.411	0.348	0.0630
7.057189542	0.411	0.348	0.0630
7.058823529	0.411	0.348	0.0630
7.062718531	0.41	0.347	0.0630
7.21009334	0.381	0.318	0.0630
7.214737794	0.38	0.317	0.0630
7.215549482	0.38	0.317	0.0630
7.216201968	0.38	0.317	0.0630
7.217269979	0.38	0.317	0.0630
7.220578567	0.379	0.316	0.0630
7.226584064	0.378	0.315	0.0630
7.237824038	0.376	0.313	0.0630
4.959890495	0.828	0.765	0.0630
4.964531871	0.828	0.765	0.0630
4.965276103	0.828	0.765	0.0630
4.969134626	0.826	0.763	0.0630
4.969878425	0.825	0.762	0.0630
4.971263419	0.824	0.761	0.0630
4.971589992	0.824	0.761	0.0630

4.971909226	0.824	0.761	0.0630
4.972221365	0.824	0.761	0.0630
4.972526643	0.824	0.761	0.0630
5.06993349	0.807	0.745	0.0620
5.070925553	0.807	0.745	0.0620
5.073027719	0.807	0.745	0.0620
5.075192221	0.807	0.745	0.0620
5.077561328	0.807	0.745	0.0620
5.079789492	0.807	0.745	0.0620
5.081524147	0.806	0.744	0.0620
5.082420071	0.806	0.744	0.0620
5.121969359	0.797	0.735	0.0620
5.126602564	0.796	0.734	0.0620
5.128869403	0.796	0.734	0.0620
5.129701904	0.796	0.734	0.0620
5.148591363	0.792	0.73	0.0620
5.149477165	0.792	0.73	0.0620
5.151537416	0.792	0.73	0.0620
5.154006704	0.792	0.73	0.0620
5.155764752	0.791	0.729	0.0620
5.156741352	0.791	0.729	0.0620
5.157563721	0.791	0.729	0.0620
5.158565203	0.791	0.729	0.0620
5.251597605	0.771	0.709	0.0620
5.252481559	0.771	0.709	0.0620
5.253700923	0.771	0.709	0.0620
6.401219512	0.54	0.478	0.0620
6.402658361	0.54	0.478	0.0620
6.40368604	0.54	0.478	0.0620
6.40486157	0.54	0.478	0.0620
6.409353557	0.539	0.477	0.0620
6.411010558	0.538	0.476	0.0620
6.411989223	0.538	0.476	0.0620
6.412628609	0.538	0.476	0.0620
6.452550638	0.531	0.469	0.0620
6.45641953	0.53	0.468	0.0620
6.457738095	0.53	0.468	0.0620
6.458980331	0.53	0.468	0.0620
6.838472486	0.452	0.39	0.0620
6.915789474	0.436	0.374	0.0620
6.917550726	0.436	0.374	0.0620
6.918023606	0.436	0.374	0.0620
6.938137755	0.432	0.37	0.0620

7.060661765	0.41	0.348	0.0620
7.064077227	0.409	0.347	0.0620
7.065942029	0.409	0.347	0.0620
7.070746133	0.408	0.346	0.0620
7.153878108	0.39	0.328	0.0620
7.157117429	0.39	0.328	0.0620
7.16012857	0.39	0.328	0.0620
7.163649836	0.389	0.327	0.0620
7.203883073	0.382	0.32	0.0620
7.205425649	0.382	0.32	0.0620
7.20683728	0.381	0.319	0.0620
7.208547267	0.381	0.319	0.0620
7.224014337	0.378	0.316	0.0620
7.226041912	0.378	0.316	0.0620
7.227903209	0.377	0.315	0.0620
7.229322696	0.377	0.315	0.0620
7.23024948	0.377	0.315	0.0620
7.233373478	0.376	0.314	0.0620
7.235442329	0.376	0.314	0.0620
7.239034887	0.375	0.313	0.0620
7.240321557	0.375	0.313	0.0620
7.242484527	0.375	0.313	0.0620
7.243842116	0.375	0.313	0.0620
7.24523565	0.375	0.313	0.0620
4.921032945	0.833	0.771	0.0620
4.929045894	0.832	0.77	0.0620
4.931031202	0.832	0.77	0.0620
4.942855277	0.829	0.767	0.0620
4.945931728	0.828	0.766	0.0620
4.947088466	0.828	0.766	0.0620
4.948431685	0.828	0.766	0.0620
4.952623581	0.828	0.766	0.0620
4.965751818	0.827	0.765	0.0620
4.966214674	0.827	0.765	0.0620
4.966665185	0.827	0.765	0.0620
4.968353163	0.826	0.764	0.0620
4.968748779	0.826	0.764	0.0620
4.972825284	0.823	0.761	0.0620
4.973258973	0.823	0.761	0.0620
4.974082131	0.823	0.761	0.0620
4.9772685	0.822	0.76	0.0620
4.988789238	0.822	0.76	0.0620
5.080647492	0.806	0.745	0.0610

5.159405068	0.79	0.729	0.0610
6.405739379	0.539	0.478	0.0610
6.406717814	0.539	0.478	0.0610
6.407818166	0.539	0.478	0.0610
6.413418291	0.537	0.476	0.0610
6.414443722	0.537	0.476	0.0610
6.416502394	0.537	0.476	0.0610
6.418414683	0.537	0.476	0.0610
6.419336003	0.537	0.476	0.0610
6.443698732	0.533	0.472	0.0610
6.44624182	0.532	0.471	0.0610
6.447034464	0.532	0.471	0.0610
6.447727926	0.532	0.471	0.0610
6.453694541	0.53	0.469	0.0610
6.454463582	0.53	0.469	0.0610
6.455361329	0.53	0.469	0.0610
7.075876504	0.406	0.345	0.0610
7.077422577	0.406	0.345	0.0610
7.079107024	0.406	0.345	0.0610
7.06716792	0.408	0.347	0.0610
7.068866434	0.408	0.347	0.0610
7.071625888	0.407	0.346	0.0610
7.072496968	0.407	0.346	0.0610
7.074000332	0.407	0.346	0.0610
7.146847566	0.391	0.33	0.0610
7.15117657	0.391	0.33	0.0610
7.151706943	0.391	0.33	0.0610
7.152108307	0.391	0.33	0.0610
7.152547829	0.391	0.33	0.0610
7.153031225	0.39	0.329	0.0610
7.160892075	0.389	0.328	0.0610
7.161726242	0.389	0.328	0.0610
7.162641365	0.389	0.328	0.0610
7.164766718	0.388	0.327	0.0610
7.189812564	0.384	0.323	0.0610
7.190931733	0.384	0.323	0.0610
7.198726115	0.383	0.322	0.0610
7.202589078	0.382	0.321	0.0610
7.231736817	0.376	0.315	0.0610
7.24734948	0.374	0.313	0.0610
7.249161074	0.374	0.313	0.0610
7.250954198	0.374	0.313	0.0610
7.252327825	0.374	0.313	0.0610

7.255769564	0.372	0.311	0.0610
7.256890013	0.372	0.311	0.0610
7.257603687	0.372	0.311	0.0610
7.258661888	0.372	0.311	0.0610
7.259766616	0.372	0.311	0.0610
4.884389201	0.838	0.777	0.0610
4.896192529	0.837	0.776	0.0610
4.901475442	0.836	0.775	0.0610
4.903842203	0.835	0.774	0.0610
4.905059488	0.835	0.774	0.0610
4.906246338	0.835	0.774	0.0610
4.907403879	0.835	0.774	0.0610
4.910711122	0.834	0.773	0.0610
4.911761653	0.834	0.773	0.0610
4.91278775	0.834	0.773	0.0610
4.918456584	0.833	0.772	0.0610
4.922678358	0.832	0.771	0.0610
4.923467396	0.832	0.771	0.0610
4.924050146	0.832	0.771	0.0610
4.925175399	0.832	0.771	0.0610
4.926468821	0.832	0.771	0.0610
4.92718275	0.832	0.771	0.0610
4.932420091	0.831	0.77	0.0610
4.93377193	0.831	0.77	0.0610
4.934637731	0.831	0.77	0.0610
4.935481185	0.831	0.77	0.0610
4.936303148	0.831	0.77	0.0610
4.937885802	0.83	0.769	0.0610
4.938647998	0.83	0.769	0.0610
4.939391713	0.83	0.769	0.0610
4.940467753	0.83	0.769	0.0610
4.9421946	0.829	0.768	0.0610
4.943501021	0.828	0.767	0.0610
4.944437585	0.828	0.767	0.0610
4.967103839	0.826	0.765	0.0610
4.967736564	0.826	0.765	0.0610
4.974746831	0.822	0.761	0.0610
4.974999375	0.822	0.761	0.0610
4.976041452	0.822	0.761	0.0610
6.421555375	0.536	0.476	0.0600
6.423599362	0.536	0.476	0.0600
6.424411715	0.535	0.475	0.0600
6.424725736	0.535	0.475	0.0600

6.426720947	0.535	0.475	0.0600
6.430659983	0.535	0.475	0.0600
6.432934779	0.535	0.475	0.0600
6.433343726	0.534	0.474	0.0600
6.43383748	0.534	0.474	0.0600
6.43513937	0.534	0.474	0.0600
6.436475911	0.534	0.474	0.0600
6.438875052	0.533	0.473	0.0600
6.44117348	0.533	0.473	0.0600
6.445113788	0.532	0.472	0.0600
6.448681397	0.531	0.471	0.0600
6.449489752	0.531	0.471	0.0600
6.450658523	0.531	0.471	0.0600
7.081812652	0.405	0.345	0.0600
7.0859035	0.404	0.344	0.0600
7.086353444	0.404	0.344	0.0600
7.087610884	0.404	0.344	0.0600
7.166010499	0.387	0.327	0.0600
7.167670683	0.387	0.327	0.0600
7.16924301	0.387	0.327	0.0600
7.170431976	0.387	0.327	0.0600
7.171733212	0.387	0.327	0.0600
7.173163418	0.387	0.327	0.0600
7.180347617	0.385	0.325	0.0600
7.182153323	0.385	0.325	0.0600
7.183503319	0.385	0.325	0.0600
7.184546616	0.385	0.325	0.0600
7.186342593	0.384	0.324	0.0600
7.188521242	0.384	0.324	0.0600
7.193012713	0.383	0.323	0.0600
7.195607151	0.383	0.323	0.0600
7.197210963	0.383	0.323	0.0600
7.201190476	0.382	0.322	0.0600
7.25313419	0.373	0.313	0.0600
7.254211544	0.373	0.313	0.0600
7.260710235	0.371	0.311	0.0600
7.263385748	0.371	0.311	0.0600
7.265906025	0.371	0.311	0.0600
7.331439394	0.358	0.298	0.0600
7.337895917	0.357	0.297	0.0600
7.338419587	0.357	0.297	0.0600
7.343203671	0.356	0.296	0.0600
7.344714506	0.356	0.296	0.0600

7.346308894	0.356	0.296	0.0600
7.347711812	0.355	0.295	0.0600
4.87389479	0.84	0.78	0.0600
4.883175019	0.838	0.778	0.0600
4.88697318	0.837	0.777	0.0600
4.890096618	0.837	0.777	0.0600
4.891695339	0.837	0.777	0.0600
4.892279725	0.837	0.777	0.0600
4.89304507	0.837	0.777	0.0600
4.894725177	0.837	0.777	0.0600
4.89672947	0.836	0.776	0.0600
4.8974332	0.836	0.776	0.0600
4.898644021	0.836	0.776	0.0600
4.89966443	0.836	0.776	0.0600
4.900331126	0.836	0.776	0.0600
4.900826175	0.836	0.776	0.0600
4.902593295	0.835	0.775	0.0600
4.908533185	0.834	0.774	0.0600
4.909635275	0.834	0.774	0.0600
4.913790256	0.833	0.773	0.0600
4.914769976	0.833	0.773	0.0600
4.916190655	0.833	0.773	0.0600
4.93710443	0.83	0.77	0.0600
4.941518468	0.829	0.769	0.0600
6.424041742	0.535	0.476	0.0590
6.437140805	0.533	0.474	0.0590
6.437928082	0.533	0.474	0.0590
7.08452381	0.404	0.345	0.0590
7.089079917	0.403	0.344	0.0590
7.09087541	0.403	0.344	0.0590
7.093396588	0.403	0.344	0.0590
7.095684394	0.402	0.343	0.0590
7.097769813	0.402	0.343	0.0590
7.099001037	0.401	0.342	0.0590
7.099914179	0.401	0.342	0.0590
7.101020496	0.401	0.342	0.0590
7.102860502	0.4	0.341	0.0590
7.104355717	0.4	0.341	0.0590
7.106090698	0.4	0.341	0.0590
7.107178128	0.399	0.34	0.0590
7.107935876	0.399	0.34	0.0590
7.108904367	0.399	0.34	0.0590
7.110243056	0.399	0.34	0.0590

7.138844302	0.392	0.333	0.0590
7.142857143	0.392	0.333	0.0590
7.174742781	0.386	0.327	0.0590
7.176743315	0.386	0.327	0.0590
7.178700645	0.386	0.327	0.0590
7.266426859	0.37	0.311	0.0590
7.266873706	0.37	0.311	0.0590
7.329369001	0.358	0.299	0.0590
7.33530572	0.357	0.298	0.0590
7.337470222	0.357	0.298	0.0590
7.34068351	0.356	0.297	0.0590
7.349739113	0.354	0.295	0.0590
7.351967277	0.354	0.295	0.0590
4.711790173	0.868	0.809	0.0590
4.866488414	0.841	0.782	0.0590
4.867195767	0.841	0.782	0.0590
4.87045241	0.84	0.781	0.0590
4.872445659	0.84	0.781	0.0590
4.873257084	0.84	0.781	0.0590
4.875457535	0.839	0.78	0.0590
4.877885132	0.839	0.78	0.0590
4.879372563	0.839	0.78	0.0590
4.881652661	0.838	0.779	0.0590
4.882491025	0.838	0.779	0.0590
7.101861001	0.4	0.342	0.0580
7.111893584	0.398	0.34	0.0580
7.113385008	0.398	0.34	0.0580
7.134324633	0.392	0.334	0.0580
7.134667192	0.392	0.334	0.0580
7.269904009	0.369	0.311	0.0580
7.328979835	0.358	0.3	0.0580
7.353889943	0.353	0.295	0.0580
7.355105305	0.353	0.295	0.0580
7.356931233	0.353	0.295	0.0580
7.363152138	0.352	0.294	0.0580
7.364602975	0.351	0.293	0.0580
7.365067163	0.351	0.293	0.0580
7.366845257	0.351	0.293	0.0580
7.368421053	0.351	0.293	0.0580
7.370107962	0.35	0.292	0.0580
7.372028823	0.35	0.292	0.0580
7.37754898	0.349	0.291	0.0580
7.378679563	0.349	0.291	0.0580

4.707438531	0.868	0.81	0.0580
4.710266585	0.868	0.81	0.0580
4.712342781	0.867	0.809	0.0580
4.713009737	0.867	0.809	0.0580
4.714642444	0.867	0.809	0.0580
4.721626133	0.866	0.808	0.0580
4.722920156	0.866	0.808	0.0580
4.723772235	0.866	0.808	0.0580
4.774031448	0.856	0.798	0.0580
4.775156476	0.856	0.798	0.0580
4.776594733	0.856	0.798	0.0580
4.796309467	0.852	0.794	0.0580
4.806080791	0.851	0.793	0.0580
4.824850999	0.847	0.789	0.0580
4.826583801	0.847	0.789	0.0580
4.828565831	0.847	0.789	0.0580
4.830503064	0.846	0.788	0.0580
4.83183772	0.846	0.788	0.0580
4.832375325	0.846	0.788	0.0580
4.832934609	0.846	0.788	0.0580
4.834249084	0.846	0.788	0.0580
4.845829636	0.844	0.786	0.0580
4.847711812	0.844	0.786	0.0580
4.851115709	0.843	0.785	0.0580
4.851778077	0.843	0.785	0.0580
4.85794996	0.842	0.784	0.0580
4.861494782	0.841	0.783	0.0580
4.862446076	0.841	0.783	0.0580
4.863939282	0.841	0.783	0.0580
4.865587513	0.841	0.783	0.0580
4.86807296	0.84	0.782	0.0580
4.8687655	0.84	0.782	0.0580
4.880235227	0.838	0.78	0.0580
4.581143187	0.885	0.828	0.0570
4.584349593	0.884	0.827	0.0570
4.585500606	0.884	0.827	0.0570
4.58581131	0.884	0.827	0.0570
4.586808064	0.884	0.827	0.0570
4.590679451	0.883	0.826	0.0570
4.591515852	0.883	0.826	0.0570
4.592429995	0.882	0.825	0.0570
4.593259595	0.882	0.825	0.0570
4.594045265	0.882	0.825	0.0570

4.65042216	0.875	0.818	0.0570
4.651784604	0.875	0.818	0.0570
4.653247234	0.875	0.818	0.0570
7.114739288	0.397	0.34	0.0570
7.115974516	0.397	0.34	0.0570
7.117105738	0.397	0.34	0.0570
7.118823529	0.397	0.34	0.0570
7.120606061	0.397	0.34	0.0570
7.12175714	0.396	0.339	0.0570
7.126092642	0.394	0.337	0.0570
7.129109864	0.394	0.337	0.0570
7.132067943	0.394	0.337	0.0570
7.133952152	0.392	0.335	0.0570
7.272727273	0.368	0.311	0.0570
7.279161665	0.367	0.31	0.0570
7.287435456	0.366	0.309	0.0570
7.292242173	0.365	0.308	0.0570
7.321548042	0.36	0.303	0.0570
7.323389154	0.36	0.303	0.0570
7.326912835	0.359	0.302	0.0570
7.359020564	0.352	0.295	0.0570
7.360330836	0.352	0.295	0.0570
7.361537151	0.352	0.295	0.0570
7.373631387	0.349	0.292	0.0570
7.375443262	0.349	0.292	0.0570
7.376467853	0.349	0.292	0.0570
7.380131363	0.348	0.291	0.0570
7.381751358	0.348	0.291	0.0570
7.386840308	0.347	0.29	0.0570
7.388297367	0.347	0.29	0.0570
4.699986666	0.869	0.812	0.0570
4.7028365	0.868	0.811	0.0570
4.704426325	0.868	0.811	0.0570
4.705882353	0.868	0.811	0.0570
4.716445111	0.866	0.809	0.0570
4.717464925	0.866	0.809	0.0570
4.718524921	0.866	0.809	0.0570
4.720065583	0.866	0.809	0.0570
4.724600553	0.865	0.808	0.0570
4.725651061	0.865	0.808	0.0570
4.726650062	0.865	0.808	0.0570
4.729399896	0.864	0.807	0.0570
4.744243851	0.861	0.804	0.0570

4.747155095	0.86	0.803	0.0570
4.74803905	0.86	0.803	0.0570
4.749301676	0.86	0.803	0.0570
4.750690608	0.86	0.803	0.0570
4.760760073	0.858	0.801	0.0570
4.76599007	0.857	0.8	0.0570
4.7668985	0.857	0.8	0.0570
4.767980189	0.857	0.8	0.0570
4.769316731	0.857	0.8	0.0570
4.770678386	0.857	0.8	0.0570
4.77329831	0.856	0.799	0.0570
4.777423921	0.855	0.798	0.0570
4.778594771	0.855	0.798	0.0570
4.779815772	0.855	0.798	0.0570
4.783777466	0.854	0.797	0.0570
4.785111155	0.854	0.797	0.0570
4.791042914	0.853	0.796	0.0570
4.793709978	0.852	0.795	0.0570
4.794921093	0.852	0.795	0.0570
4.797492579	0.851	0.794	0.0570
4.79794707	0.851	0.794	0.0570
4.800233083	0.851	0.794	0.0570
4.802445733	0.851	0.794	0.0570
4.803276574	0.851	0.794	0.0570
4.804195421	0.851	0.794	0.0570
4.808217739	0.85	0.793	0.0570
4.81382452	0.849	0.792	0.0570
4.815943993	0.848	0.791	0.0570
4.817295709	0.848	0.791	0.0570
4.818085549	0.848	0.791	0.0570
4.818964979	0.848	0.791	0.0570
4.82035284	0.847	0.79	0.0570
4.821881798	0.847	0.79	0.0570
4.822932219	0.847	0.79	0.0570
4.823825007	0.847	0.79	0.0570
4.836060678	0.845	0.788	0.0570
4.8378331	0.845	0.788	0.0570
4.838709677	0.845	0.788	0.0570
4.839567605	0.845	0.788	0.0570
4.840594932	0.844	0.787	0.0570
4.841765813	0.844	0.787	0.0570
4.843743896	0.844	0.787	0.0570
4.849242424	0.843	0.786	0.0570

4.850149701	0.843	0.786	0.0570
4.850522835	0.843	0.786	0.0570
4.852936088	0.842	0.785	0.0570
4.854085051	0.842	0.785	0.0570
4.854930131	0.842	0.785	0.0570
4.85563028	0.842	0.785	0.0570
4.856456044	0.842	0.785	0.0570
4.858955996	0.841	0.784	0.0570
4.86013302	0.841	0.784	0.0570
4.487492183	0.898	0.842	0.0560
4.488220621	0.898	0.842	0.0560
4.494026454	0.897	0.841	0.0560
4.494897433	0.897	0.841	0.0560
4.496227132	0.897	0.841	0.0560
4.554223028	0.889	0.833	0.0560
4.554714634	0.889	0.833	0.0560
4.570660522	0.887	0.831	0.0560
4.580825374	0.885	0.829	0.0560
4.582307061	0.884	0.828	0.0560
4.583333333	0.884	0.828	0.0560
4.58793208	0.883	0.827	0.0560
4.588803637	0.883	0.827	0.0560
4.589767958	0.883	0.827	0.0560
4.595433943	0.881	0.825	0.0560
4.596714845	0.881	0.825	0.0560
4.597428774	0.881	0.825	0.0560
4.598850575	0.881	0.825	0.0560
4.649165089	0.875	0.819	0.0560
4.655086353	0.874	0.818	0.0560
4.656809451	0.874	0.818	0.0560
4.65795967	0.874	0.818	0.0560
4.658738001	0.874	0.818	0.0560
4.660626536	0.874	0.818	0.0560
4.662541755	0.874	0.818	0.0560
4.663299239	0.873	0.817	0.0560
4.665171898	0.873	0.817	0.0560
4.668135095	0.873	0.817	0.0560
4.669707422	0.873	0.817	0.0560
4.688247608	0.87	0.814	0.0560
4.689130376	0.87	0.814	0.0560
4.689460355	0.87	0.814	0.0560
4.69079915	0.87	0.814	0.0560
4.692859943	0.869	0.813	0.0560

4.693430004	0.869	0.813	0.0560
4.695932064	0.869	0.813	0.0560
7.122794915	0.395	0.339	0.0560
7.123735339	0.394	0.338	0.0560
7.124591503	0.394	0.338	0.0560
7.125374251	0.394	0.338	0.0560
7.132610037	0.393	0.337	0.0560
7.133100233	0.392	0.336	0.0560
7.133545648	0.392	0.336	0.0560
7.275604143	0.367	0.311	0.0560
7.278696289	0.367	0.311	0.0560
7.279705882	0.366	0.31	0.0560
7.282384106	0.366	0.31	0.0560
7.285241249	0.366	0.31	0.0560
7.289739604	0.365	0.309	0.0560
7.290994624	0.365	0.309	0.0560
7.293025381	0.364	0.308	0.0560
7.294415283	0.364	0.308	0.0560
7.296447391	0.364	0.308	0.0560
7.298073936	0.364	0.308	0.0560
7.299060324	0.364	0.308	0.0560
7.309018568	0.362	0.306	0.0560
7.317138411	0.36	0.304	0.0560
7.319551009	0.36	0.304	0.0560
7.324392213	0.359	0.303	0.0560
7.32521108	0.359	0.303	0.0560
7.328505699	0.358	0.302	0.0560
7.38358286	0.347	0.291	0.0560
7.38511815	0.347	0.291	0.0560
7.38991969	0.346	0.29	0.0560
7.391304348	0.346	0.29	0.0560
7.392621871	0.346	0.29	0.0560
4.702183854	0.868	0.812	0.0560
4.702541828	0.868	0.812	0.0560
4.727766798	0.864	0.808	0.0560
4.730860859	0.863	0.807	0.0560
4.732258065	0.863	0.807	0.0560
4.733530572	0.863	0.807	0.0560
4.735284958	0.863	0.807	0.0560
4.736842105	0.863	0.807	0.0560
4.740072067	0.862	0.806	0.0560
4.745330336	0.86	0.804	0.0560
4.746299077	0.86	0.804	0.0560

4.752233818	0.859	0.803	0.0560
4.75359239	0.859	0.803	0.0560
4.754671558	0.859	0.803	0.0560
4.755671158	0.859	0.803	0.0560
4.757359125	0.858	0.802	0.0560
4.758828417	0.858	0.802	0.0560
4.759197217	0.858	0.802	0.0560
4.759486837	0.858	0.802	0.0560
4.763305322	0.857	0.801	0.0560
4.765165441	0.857	0.801	0.0560
4.771407717	0.856	0.8	0.0560
4.772150438	0.856	0.8	0.0560
4.780353793	0.854	0.798	0.0560
4.78154825	0.854	0.798	0.0560
4.782608696	0.854	0.798	0.0560
4.786577431	0.853	0.797	0.0560
4.788305591	0.853	0.797	0.0560
4.789103039	0.853	0.797	0.0560
4.789946423	0.853	0.797	0.0560
4.792283037	0.852	0.796	0.0560
4.809133489	0.849	0.793	0.0560
4.809825196	0.849	0.793	0.0560
4.811313291	0.849	0.793	0.0560
4.812667112	0.849	0.793	0.0560
4.480020492	0.899	0.844	0.0550
4.484692284	0.898	0.843	0.0550
4.485667486	0.898	0.843	0.0550
4.486832987	0.898	0.843	0.0550
4.489216141	0.897	0.842	0.0550
4.491274771	0.897	0.842	0.0550
4.493212255	0.897	0.842	0.0550
4.497197959	0.896	0.841	0.0550
4.498677249	0.896	0.841	0.0550
4.501184834	0.896	0.841	0.0550
4.52091227	0.893	0.838	0.0550
4.532705745	0.891	0.836	0.0550
4.536052246	0.89	0.835	0.0550
4.538407625	0.89	0.835	0.0550
4.540757144	0.89	0.835	0.0550
4.543369475	0.89	0.835	0.0550
4.545454545	0.89	0.835	0.0550
4.549678112	0.889	0.834	0.0550
4.550280899	0.889	0.834	0.0550

4.550730001	0.889	0.834	0.0550
4.551090127	0.889	0.834	0.0550
4.552636331	0.889	0.834	0.0550
4.555264689	0.888	0.833	0.0550
4.55625879	0.888	0.833	0.0550
4.557392577	0.888	0.833	0.0550
4.559131345	0.888	0.833	0.0550
4.560921535	0.888	0.833	0.0550
4.569210196	0.887	0.832	0.0550
4.573295985	0.886	0.831	0.0550
4.575317548	0.886	0.831	0.0550
4.575871442	0.886	0.831	0.0550
4.576692807	0.886	0.831	0.0550
4.579479207	0.885	0.83	0.0550
4.602631579	0.88	0.825	0.0550
4.6062965	0.88	0.825	0.0550
4.608859727	0.88	0.825	0.0550
4.610584027	0.88	0.825	0.0550
4.610944777	0.879	0.824	0.0550
4.611255037	0.879	0.824	0.0550
4.61339179	0.879	0.824	0.0550
4.615384615	0.879	0.824	0.0550
4.634781504	0.877	0.822	0.0550
4.635589128	0.877	0.822	0.0550
4.637712863	0.876	0.821	0.0550
4.638864737	0.876	0.821	0.0550
4.639849067	0.876	0.821	0.0550
4.640703427	0.876	0.821	0.0550
4.64187056	0.876	0.821	0.0550
4.646110057	0.875	0.82	0.0550
4.647853736	0.875	0.82	0.0550
4.670070496	0.872	0.817	0.0550
4.670494176	0.872	0.817	0.0550
4.672012758	0.872	0.817	0.0550
4.673639939	0.872	0.817	0.0550
4.674234628	0.872	0.817	0.0550
4.675970795	0.871	0.816	0.0550
4.677018135	0.871	0.816	0.0550
4.677890964	0.871	0.816	0.0550
4.678924876	0.871	0.816	0.0550
4.680169122	0.871	0.816	0.0550
4.684962406	0.87	0.815	0.0550
4.686607143	0.87	0.815	0.0550

4.692340279	0.869	0.814	0.0550
7.300428687	0.363	0.308	0.0550
7.30210944	0.363	0.308	0.0550
7.303798059	0.363	0.308	0.0550
7.306328423	0.362	0.307	0.0550
7.310634599	0.361	0.306	0.0550
7.311712185	0.361	0.306	0.0550
7.312966418	0.361	0.306	0.0550
7.314434539	0.36	0.305	0.0550
7.394152796	0.345	0.29	0.0550
7.395407951	0.345	0.29	0.0550
7.396854989	0.345	0.29	0.0550
4.738212719	0.862	0.807	0.0550
4.739733863	0.862	0.807	0.0550
4.741263891	0.861	0.806	0.0550
4.742562592	0.861	0.806	0.0550
4.743223443	0.861	0.806	0.0550
4.477635897	0.899	0.845	0.0540
4.478847137	0.899	0.845	0.0540
4.481003196	0.898	0.844	0.0540
4.481945348	0.898	0.844	0.0540
4.483647352	0.898	0.844	0.0540
4.502493735	0.895	0.841	0.0540
4.502770889	0.895	0.841	0.0540
4.503117617	0.895	0.841	0.0540
4.503907881	0.895	0.841	0.0540
4.5047275	0.895	0.841	0.0540
4.505561667	0.895	0.841	0.0540
4.50742168	0.894	0.84	0.0540
4.509237221	0.894	0.84	0.0540
4.510336743	0.894	0.84	0.0540
4.511845039	0.894	0.84	0.0540
4.513553114	0.894	0.84	0.0540
4.515207373	0.894	0.84	0.0540
4.516129032	0.894	0.84	0.0540
4.516947765	0.893	0.839	0.0540
4.517919393	0.893	0.839	0.0540
4.51892315	0.893	0.839	0.0540
4.520160978	0.893	0.839	0.0540
4.521784795	0.892	0.838	0.0540
4.522453029	0.892	0.838	0.0540
4.523211295	0.892	0.838	0.0540
4.529899253	0.891	0.837	0.0540

4.53081837	0.891	0.837	0.0540
4.534522606	0.89	0.836	0.0540
4.535201423	0.89	0.836	0.0540
4.547405384	0.889	0.835	0.0540
4.561951754	0.887	0.833	0.0540
4.562803398	0.887	0.833	0.0540
4.56360468	0.887	0.833	0.0540
4.564659978	0.887	0.833	0.0540
4.565217391	0.887	0.833	0.0540
4.566073263	0.887	0.833	0.0540
4.567728526	0.887	0.833	0.0540
4.577289608	0.885	0.831	0.0540
4.577889021	0.885	0.831	0.0540
4.617633828	0.878	0.824	0.0540
4.620068103	0.878	0.824	0.0540
4.62047141	0.878	0.824	0.0540
4.620950888	0.878	0.824	0.0540
4.62216385	0.878	0.824	0.0540
4.623385746	0.878	0.824	0.0540
4.623966685	0.878	0.824	0.0540
4.627898927	0.877	0.823	0.0540
4.630604288	0.877	0.823	0.0540
4.632303235	0.877	0.823	0.0540
4.633586932	0.877	0.823	0.0540
4.636316549	0.876	0.822	0.0540
4.643832943	0.875	0.821	0.0540
4.644985017	0.875	0.821	0.0540
4.674940444	0.871	0.817	0.0540
4.682197684	0.87	0.816	0.0540
4.683877415	0.87	0.816	0.0540
7.408739675	0.343	0.289	0.0540
7.413776699	0.342	0.288	0.0540
7.416092265	0.342	0.288	0.0540
7.423777654	0.341	0.287	0.0540
7.438262195	0.337	0.283	0.0540
7.439512195	0.337	0.283	0.0540
7.440930233	0.337	0.283	0.0540
7.398052102	0.344	0.29	0.0540
7.399421965	0.344	0.29	0.0540
7.400787402	0.344	0.29	0.0540
7.402086103	0.344	0.29	0.0540
4.468679425	0.901	0.848	0.0530
4.469930989	0.901	0.848	0.0530

4.475941154	0.9	0.847	0.0530
4.526055705	0.891	0.838	0.0530
4.528856826	0.891	0.838	0.0530
4.624638728	0.877	0.824	0.0530
4.625584112	0.877	0.824	0.0530
7.403588778	0.343	0.29	0.0530
7.404821722	0.343	0.29	0.0530
7.406235349	0.343	0.29	0.0530
7.411329678	0.342	0.289	0.0530
7.418286691	0.341	0.288	0.0530
7.420369243	0.341	0.288	0.0530
7.422348266	0.341	0.288	0.0530
7.425356506	0.34	0.287	0.0530
7.426685587	0.34	0.287	0.0530
7.429563492	0.339	0.286	0.0530
7.435053981	0.337	0.284	0.0530
7.436698718	0.337	0.284	0.0530
7.442734744	0.336	0.283	0.0530
7.444026734	0.336	0.283	0.0530
7.444849959	0.336	0.283	0.0530
4.461229744	0.902	0.85	0.0520
4.46547619	0.901	0.849	0.0520
4.467375887	0.901	0.849	0.0520
4.471318962	0.9	0.848	0.0520
4.47286695	0.9	0.848	0.0520
4.474411166	0.9	0.848	0.0520
7.427736007	0.339	0.287	0.0520
7.431493994	0.338	0.286	0.0520
7.433321479	0.338	0.286	0.0520
7.446031993	0.335	0.283	0.0520
7.447542186	0.335	0.283	0.0520
7.456780924	0.333	0.281	0.0520
7.459666927	0.333	0.281	0.0520
7.460927961	0.333	0.281	0.0520
7.463227342	0.332	0.28	0.0520
4.451043282	0.903	0.852	0.0510
4.451416208	0.903	0.852	0.0510
4.452880687	0.903	0.852	0.0510
4.454797008	0.903	0.852	0.0510
4.455702047	0.903	0.852	0.0510
4.457487091	0.902	0.851	0.0510
4.458215026	0.902	0.851	0.0510
4.459346311	0.902	0.851	0.0510

4.463325653	0.901	0.85	0.0510
7.448970146	0.334	0.283	0.0510
7.450322411	0.334	0.283	0.0510
7.451604846	0.334	0.283	0.0510
7.453980802	0.333	0.282	0.0510
7.462112514	0.332	0.281	0.0510
7.464278424	0.331	0.28	0.0510
7.465271079	0.331	0.28	0.0510
4.435429757	0.905	0.855	0.0500
4.43835246	0.904	0.854	0.0500
4.438763532	0.904	0.854	0.0500
4.441734417	0.904	0.854	0.0500
4.444444444	0.904	0.854	0.0500
4.450405615	0.903	0.853	0.0500
4.450708251	0.903	0.853	0.0500
4.456240144	0.902	0.852	0.0500
4.456832298	0.902	0.852	0.0500
7.452822724	0.333	0.283	0.0500
7.466210046	0.33	0.28	0.0500
7.467099567	0.33	0.28	0.0500
7.467943449	0.33	0.28	0.0500
4.366649163	0.914	0.865	0.0490
4.368118572	0.914	0.865	0.0490
4.39895288	0.91	0.861	0.0490
4.432711062	0.905	0.856	0.0490
4.438036035	0.904	0.855	0.0490
4.447353112	0.903	0.854	0.0490
7.469471333	0.329	0.28	0.0490
7.471249174	0.329	0.28	0.0490
7.473068458	0.329	0.28	0.0490
7.477270882	0.329	0.28	0.0490
7.480615496	0.329	0.28	0.0490
7.481198756	0.329	0.28	0.0490
7.483218453	0.328	0.279	0.0490
7.483657338	0.328	0.279	0.0490
7.484653335	0.327	0.278	0.0490
7.485205029	0.327	0.278	0.0490
7.485547201	0.327	0.278	0.0490
7.492857143	0.327	0.278	0.0490
7.514637904	0.324	0.275	0.0490
7.515339733	0.324	0.275	0.0490
7.515725759	0.324	0.275	0.0490
7.516131718	0.324	0.275	0.0490

7.516790624	0.324	0.275	0.0490
4.343145743	0.918	0.87	0.0480
4.346687913	0.917	0.869	0.0480
4.347826087	0.917	0.869	0.0480
4.348913043	0.917	0.869	0.0480
4.350141243	0.917	0.869	0.0480
4.351611831	0.917	0.869	0.0480
4.354452848	0.916	0.868	0.0480
4.355333465	0.916	0.868	0.0480
4.356131932	0.916	0.868	0.0480
4.356988772	0.916	0.868	0.0480
4.359719738	0.915	0.867	0.0480
4.361440612	0.915	0.867	0.0480
4.362580603	0.915	0.867	0.0480
4.363692991	0.915	0.867	0.0480
4.365061559	0.914	0.866	0.0480
4.369395712	0.913	0.865	0.0480
4.370615649	0.913	0.865	0.0480
4.371059206	0.913	0.865	0.0480
4.371421092	0.913	0.865	0.0480
4.371721998	0.913	0.865	0.0480
4.373429648	0.913	0.865	0.0480
4.376849112	0.912	0.864	0.0480
4.378891596	0.912	0.864	0.0480
4.38055372	0.912	0.864	0.0480
4.382369261	0.912	0.864	0.0480
4.38639092	0.911	0.863	0.0480
4.388321995	0.911	0.863	0.0480
4.389566396	0.911	0.863	0.0480
4.39774806	0.91	0.862	0.0480
4.400956938	0.909	0.861	0.0480
4.402043894	0.909	0.861	0.0480
4.403467909	0.909	0.861	0.0480
4.405770783	0.909	0.861	0.0480
4.4071813	0.909	0.861	0.0480
4.412360086	0.908	0.86	0.0480
4.413050503	0.908	0.86	0.0480
4.413276681	0.908	0.86	0.0480
4.413600462	0.908	0.86	0.0480
4.41434336	0.908	0.86	0.0480
4.42476489	0.906	0.858	0.0480
4.426185345	0.906	0.858	0.0480
4.429376973	0.905	0.857	0.0480

4.429954974	0.905	0.857	0.0480
4.431098964	0.905	0.857	0.0480
4.432125307	0.905	0.857	0.0480
7.481747935	0.328	0.28	0.0480
7.482265936	0.328	0.28	0.0480
7.48275534	0.328	0.28	0.0480
7.484073849	0.327	0.279	0.0480
7.512886939	0.324	0.276	0.0480
7.513968224	0.324	0.276	0.0480
7.517485938	0.323	0.275	0.0480
7.517989336	0.323	0.275	0.0480
7.549027985	0.318	0.27	0.0480
7.550344641	0.318	0.27	0.0480
7.55173398	0.317	0.269	0.0480
7.553202193	0.317	0.269	0.0480
4.344492041	0.917	0.87	0.0470
4.353504081	0.916	0.869	0.0470
4.358258129	0.915	0.868	0.0470
4.358974359	0.915	0.868	0.0470
4.383138847	0.911	0.864	0.0470
4.383899049	0.911	0.864	0.0470
4.384631596	0.911	0.864	0.0470
4.390889147	0.91	0.863	0.0470
4.393219425	0.91	0.863	0.0470
4.395254427	0.91	0.863	0.0470
4.396383758	0.91	0.863	0.0470
4.397376741	0.91	0.863	0.0470
4.409673822	0.908	0.861	0.0470
4.415239016	0.907	0.86	0.0470
4.415914035	0.907	0.86	0.0470
4.416710784	0.907	0.86	0.0470
4.417891283	0.907	0.86	0.0470
4.419247077	0.907	0.86	0.0470
4.420829105	0.906	0.859	0.0470
4.42193963	0.906	0.859	0.0470
4.422593738	0.906	0.859	0.0470
4.423659674	0.906	0.859	0.0470
4.427472015	0.905	0.858	0.0470
4.42854222	0.905	0.858	0.0470
7.505980861	0.324	0.277	0.0470
7.518522584	0.322	0.275	0.0470
7.519088419	0.322	0.275	0.0470
7.519689922	0.322	0.275	0.0470

7.542946893	0.318	0.271	0.0470
7.546039995	0.318	0.271	0.0470
7.547778454	0.318	0.271	0.0470
7.554756195	0.316	0.269	0.0470
7.556403732	0.316	0.269	0.0470
7.568314171	0.314	0.267	0.0470
7.570244672	0.314	0.267	0.0470
7.571841453	0.314	0.267	0.0470
7.572891874	0.314	0.267	0.0470
7.611001596	0.307	0.26	0.0470
7.634361461	0.303	0.256	0.0470
4.27872656	0.925	0.879	0.0460
4.28228022	0.925	0.879	0.0460
4.289083558	0.924	0.878	0.0460
4.292567879	0.924	0.878	0.0460
4.292938322	0.924	0.878	0.0460
4.293335989	0.924	0.878	0.0460
4.293631786	0.924	0.878	0.0460
4.297409461	0.923	0.877	0.0460
4.299758454	0.923	0.877	0.0460
4.336747705	0.919	0.873	0.0460
4.338429816	0.919	0.873	0.0460
4.340863952	0.918	0.872	0.0460
4.342481203	0.918	0.872	0.0460
7.523157895	0.321	0.275	0.0460
7.527863777	0.321	0.275	0.0460
7.530528667	0.321	0.275	0.0460
7.532056551	0.321	0.275	0.0460
7.532900433	0.321	0.275	0.0460
7.533789954	0.32	0.274	0.0460
7.534728921	0.32	0.274	0.0460
7.535721576	0.32	0.274	0.0460
7.536772658	0.319	0.273	0.0460
7.537887486	0.319	0.273	0.0460
7.539722573	0.318	0.272	0.0460
7.557695722	0.315	0.269	0.0460
7.558597326	0.315	0.269	0.0460
7.560015364	0.315	0.269	0.0460
7.56200041	0.315	0.269	0.0460
7.563563887	0.315	0.269	0.0460
7.564946019	0.315	0.269	0.0460
7.566678521	0.315	0.269	0.0460
7.574643494	0.313	0.267	0.0460

7.576941288	0.313	0.267	0.0460
7.578871417	0.313	0.267	0.0460
7.589506964	0.311	0.265	0.0460
7.590801161	0.311	0.265	0.0460
7.592612919	0.31	0.264	0.0460
7.594460836	0.31	0.264	0.0460
7.609379273	0.307	0.261	0.0460
7.612421762	0.306	0.26	0.0460
7.614143921	0.306	0.26	0.0460
7.620499419	0.305	0.259	0.0460
7.622164421	0.305	0.259	0.0460
7.62544964	0.304	0.258	0.0460
7.632583367	0.303	0.257	0.0460
7.636465205	0.302	0.256	0.0460
7.638342082	0.302	0.256	0.0460
4.278340916	0.925	0.88	0.0450
4.293951479	0.923	0.878	0.0450
4.294298087	0.923	0.878	0.0450
4.294675161	0.923	0.878	0.0450
4.295086904	0.923	0.878	0.0450
4.302173913	0.922	0.877	0.0450
4.304491794	0.922	0.877	0.0450
4.305095659	0.922	0.877	0.0450
4.305887826	0.922	0.877	0.0450
4.306575394	0.922	0.877	0.0450
4.3073115	0.922	0.877	0.0450
4.319394328	0.921	0.876	0.0450
4.322094343	0.921	0.876	0.0450
4.331503713	0.919	0.874	0.0450
4.332565284	0.919	0.874	0.0450
4.334154351	0.919	0.874	0.0450
4.335117743	0.919	0.874	0.0450
4.33912349	0.918	0.873	0.0450
4.339622642	0.918	0.873	0.0450
7.580131498	0.312	0.267	0.0450
7.582268889	0.312	0.267	0.0450
7.58408114	0.312	0.267	0.0450
7.58523828	0.312	0.267	0.0450
7.587429689	0.311	0.266	0.0450
7.595917967	0.309	0.264	0.0450
7.598449612	0.309	0.264	0.0450
7.601169591	0.309	0.264	0.0450
7.602539454	0.309	0.264	0.0450

7.603765073	0.308	0.263	0.0450
7.605212111	0.308	0.263	0.0450
7.608028808	0.307	0.262	0.0450
7.615639328	0.305	0.26	0.0450
7.617470829	0.305	0.26	0.0450
7.622664221	0.304	0.259	0.0450
7.62397541	0.304	0.259	0.0450
7.627052204	0.303	0.258	0.0450
7.628917379	0.303	0.258	0.0450
7.630604288	0.303	0.258	0.0450
7.639320221	0.301	0.256	0.0450
7.641304348	0.301	0.256	0.0450
4.243305144	0.929	0.885	0.0440
4.244998051	0.929	0.885	0.0440
4.246560942	0.929	0.885	0.0440
4.260859333	0.927	0.883	0.0440
4.266034755	0.926	0.882	0.0440
4.267091295	0.926	0.882	0.0440
4.267904303	0.926	0.882	0.0440
4.268649265	0.926	0.882	0.0440
4.270181316	0.926	0.882	0.0440
4.271600722	0.926	0.882	0.0440
4.277617213	0.925	0.881	0.0440
4.277926322	0.925	0.881	0.0440
4.308101473	0.921	0.877	0.0440
4.309427733	0.921	0.877	0.0440
4.310861037	0.921	0.877	0.0440
4.312551368	0.921	0.877	0.0440
4.315453053	0.921	0.877	0.0440
4.322748656	0.92	0.876	0.0440
4.323620495	0.92	0.876	0.0440
4.32508351	0.92	0.876	0.0440
4.326382887	0.92	0.876	0.0440
4.327640643	0.92	0.876	0.0440
4.328813251	0.92	0.876	0.0440
4.3295826	0.919	0.875	0.0440
4.330553549	0.919	0.875	0.0440
7.606497883	0.307	0.263	0.0440
7.643084622	0.3	0.256	0.0440
7.643495131	0.3	0.256	0.0440
7.645368492	0.3	0.256	0.0440
7.647058824	0.3	0.256	0.0440
7.649032767	0.299	0.255	0.0440

7.651260931	0.299	0.255	0.0440
4.22992206	0.931	0.888	0.0430
4.236870669	0.93	0.887	0.0430
4.237691687	0.929	0.886	0.0430
4.238252917	0.929	0.886	0.0430
4.238770515	0.929	0.886	0.0430
4.240777339	0.929	0.886	0.0430
4.248008246	0.928	0.885	0.0430
4.249352332	0.928	0.885	0.0430
4.258816233	0.927	0.884	0.0430
4.259807144	0.927	0.884	0.0430
4.261829359	0.926	0.883	0.0430
4.262726488	0.926	0.883	0.0430
4.264280369	0.926	0.883	0.0430
4.274650654	0.925	0.882	0.0430
7.785986914	0.277	0.234	0.0430
7.786572394	0.277	0.234	0.0430
7.789148144	0.277	0.234	0.0430
7.809219078	0.274	0.231	0.0430
7.810434933	0.274	0.231	0.0430
7.65291807	0.298	0.255	0.0430
7.654746701	0.298	0.255	0.0430
7.655711207	0.298	0.255	0.0430
4.198504118	0.934	0.892	0.0420
4.20003923	0.934	0.892	0.0420
4.202040991	0.934	0.892	0.0420
4.205931264	0.933	0.891	0.0420
4.207483673	0.933	0.891	0.0420
4.207785533	0.933	0.891	0.0420
4.209223554	0.933	0.891	0.0420
4.211862143	0.933	0.891	0.0420
4.213340558	0.933	0.891	0.0420
4.222912353	0.932	0.89	0.0420
4.228823159	0.931	0.889	0.0420
4.231786732	0.93	0.888	0.0420
4.232966534	0.93	0.888	0.0420
4.234211476	0.93	0.888	0.0420
4.23587366	0.93	0.888	0.0420
4.250748503	0.927	0.885	0.0420
4.252185285	0.927	0.885	0.0420
4.253508605	0.927	0.885	0.0420
4.255450202	0.927	0.885	0.0420
4.25709125	0.927	0.885	0.0420

4.257899474	0.927	0.885	0.0420
7.785022513	0.278	0.236	0.0420
7.792764345	0.276	0.234	0.0420
7.794696619	0.276	0.234	0.0420
7.79594288	0.276	0.234	0.0420
7.797114609	0.276	0.234	0.0420
7.802224371	0.275	0.233	0.0420
7.811575444	0.273	0.231	0.0420
7.814700704	0.273	0.231	0.0420
7.817541613	0.273	0.231	0.0420
7.657238924	0.297	0.255	0.0420
7.658901158	0.297	0.255	0.0420
7.661054518	0.296	0.254	0.0420
7.661813992	0.296	0.254	0.0420
7.663285619	0.296	0.254	0.0420
7.665450122	0.296	0.254	0.0420
7.672094426	0.295	0.253	0.0420
7.67368729	0.294	0.252	0.0420
7.675237471	0.294	0.252	0.0420
4.178159834	0.936	0.895	0.0410
4.184228039	0.935	0.894	0.0410
4.18541456	0.935	0.894	0.0410
4.194475343	0.934	0.893	0.0410
4.196466582	0.934	0.893	0.0410
4.197822036	0.934	0.893	0.0410
4.203360103	0.933	0.892	0.0410
4.204183555	0.933	0.892	0.0410
4.214849681	0.932	0.891	0.0410
4.216541843	0.932	0.891	0.0410
4.217808735	0.932	0.891	0.0410
4.219927764	0.932	0.891	0.0410
4.221663875	0.932	0.891	0.0410
4.224477299	0.931	0.89	0.0410
4.225771294	0.931	0.89	0.0410
4.2264973	0.931	0.89	0.0410
4.227496022	0.931	0.89	0.0410
4.228379674	0.931	0.89	0.0410
7.749172185	0.281	0.24	0.0410
7.750968992	0.281	0.24	0.0410
7.752780586	0.281	0.24	0.0410
7.756253909	0.28	0.239	0.0410
7.757515473	0.28	0.239	0.0410
7.75990736	0.28	0.239	0.0410

7.761715896	0.28	0.239	0.0410
7.762697828	0.28	0.239	0.0410
7.774024024	0.279	0.238	0.0410
7.781293952	0.278	0.237	0.0410
7.785474593	0.277	0.236	0.0410
7.798180593	0.275	0.234	0.0410
7.799371069	0.275	0.234	0.0410
7.80070922	0.275	0.234	0.0410
7.805624741	0.274	0.233	0.0410
7.818865345	0.272	0.231	0.0410
7.820030846	0.272	0.231	0.0410
7.821546733	0.272	0.231	0.0410
7.848029716	0.268	0.227	0.0410
7.850038439	0.268	0.227	0.0410
7.851545761	0.268	0.227	0.0410
7.660196591	0.296	0.255	0.0410
7.667919799	0.295	0.254	0.0410
7.670202905	0.295	0.254	0.0410
7.676737846	0.293	0.252	0.0410
7.677995392	0.292	0.251	0.0410
7.679285714	0.292	0.251	0.0410
7.68057971	0.292	0.251	0.0410
7.681639313	0.292	0.251	0.0410
7.682523017	0.292	0.251	0.0410
7.686820997	0.291	0.25	0.0410
7.687287415	0.291	0.25	0.0410
7.689903846	0.291	0.25	0.0410
7.692307692	0.291	0.25	0.0410
7.738476152	0.284	0.243	0.0410
7.740830756	0.283	0.242	0.0410
7.742918961	0.283	0.242	0.0410
7.744131671	0.283	0.242	0.0410
7.745419888	0.283	0.242	0.0410
7.74686581	0.282	0.241	0.0410
7.747798559	0.282	0.241	0.0410
4.170735193	0.937	0.897	0.0400
4.170988633	0.937	0.897	0.0400
4.171275783	0.936	0.896	0.0400
4.173626374	0.936	0.896	0.0400
4.176147382	0.936	0.896	0.0400
4.176842889	0.936	0.896	0.0400
4.179499329	0.935	0.895	0.0400
4.18034257	0.935	0.895	0.0400

4.181304571	0.935	0.895	0.0400
4.182745826	0.935	0.895	0.0400
4.187264094	0.934	0.894	0.0400
4.189212905	0.934	0.894	0.0400
4.191746261	0.934	0.894	0.0400
7.754860375	0.28	0.24	0.0400
7.766194332	0.279	0.239	0.0400
7.769507471	0.279	0.239	0.0400
7.770027221	0.279	0.239	0.0400
7.822854948	0.271	0.231	0.0400
7.823329332	0.271	0.231	0.0400
7.824808184	0.271	0.231	0.0400
7.829202366	0.27	0.23	0.0400
7.830072598	0.27	0.23	0.0400
7.831427831	0.27	0.23	0.0400
7.838918919	0.269	0.229	0.0400
7.840863309	0.269	0.229	0.0400
7.842431937	0.269	0.229	0.0400
7.846688034	0.268	0.228	0.0400
7.852100423	0.267	0.227	0.0400
7.852554865	0.267	0.227	0.0400
7.854951797	0.267	0.227	0.0400
7.862332193	0.266	0.226	0.0400
7.877750104	0.263	0.223	0.0400
7.879611331	0.263	0.223	0.0400
7.880614742	0.263	0.223	0.0400
7.881573822	0.263	0.223	0.0400
7.88278231	0.262	0.222	0.0400
7.883913532	0.262	0.222	0.0400
7.885969664	0.262	0.222	0.0400
7.935995904	0.255	0.215	0.0400
7.937003968	0.255	0.215	0.0400
7.938215649	0.255	0.215	0.0400
7.684746997	0.291	0.251	0.0400
7.694638695	0.29	0.25	0.0400
7.697169059	0.29	0.25	0.0400
7.697605074	0.29	0.25	0.0400
7.698127213	0.29	0.25	0.0400
7.700557701	0.29	0.25	0.0400
7.721053611	0.287	0.247	0.0400
7.725705329	0.286	0.246	0.0400
7.727272727	0.286	0.246	0.0400
7.736537667	0.284	0.244	0.0400

4.035616149	0.948	0.908	0.0400
4.037776723	0.948	0.908	0.0400
4.041926656	0.948	0.908	0.0400
4.045478036	0.948	0.908	0.0400
4.047065338	0.948	0.908	0.0400
4.129192746	0.941	0.901	0.0400
4.147034368	0.939	0.899	0.0400
4.148331721	0.939	0.899	0.0400
4.149468085	0.939	0.899	0.0400
4.150471698	0.938	0.898	0.0400
4.152394775	0.938	0.898	0.0400
4.154548648	0.938	0.898	0.0400
4.156277256	0.938	0.898	0.0400
4.157599054	0.938	0.898	0.0400
4.158386621	0.938	0.898	0.0400
4.160657526	0.937	0.898	0.0390
4.164551607	0.937	0.898	0.0390
4.166666667	0.937	0.898	0.0390
4.16864139	0.937	0.898	0.0390
7.827517162	0.27	0.231	0.0390
7.833281368	0.269	0.23	0.0390
7.8351079	0.269	0.23	0.0390
7.836829367	0.269	0.23	0.0390
7.844645551	0.268	0.229	0.0390
7.857142857	0.266	0.227	0.0390
7.859389039	0.266	0.227	0.0390
7.861852093	0.266	0.227	0.0390
7.86388198	0.265	0.226	0.0390
7.865511099	0.265	0.226	0.0390
7.866260163	0.265	0.226	0.0390
7.867156863	0.265	0.226	0.0390
7.868249759	0.265	0.226	0.0390
7.869337472	0.265	0.226	0.0390
7.871654084	0.264	0.225	0.0390
7.873670213	0.264	0.225	0.0390
7.888974472	0.261	0.222	0.0390
7.890890731	0.261	0.222	0.0390
7.892946652	0.261	0.222	0.0390
7.917870906	0.258	0.219	0.0390
7.919269116	0.258	0.219	0.0390
7.925227114	0.257	0.218	0.0390
7.927248677	0.257	0.218	0.0390
7.932183908	0.256	0.217	0.0390

7.933608815	0.255	0.216	0.0390
7.934684084	0.255	0.216	0.0390
7.940053884	0.254	0.215	0.0390
7.942219441	0.254	0.215	0.0390
7.703203203	0.289	0.25	0.0390
7.704310868	0.289	0.25	0.0390
7.706625683	0.289	0.25	0.0390
7.715167549	0.288	0.249	0.0390
7.716292408	0.288	0.249	0.0390
7.718709525	0.287	0.248	0.0390
7.71994324	0.287	0.248	0.0390
7.722828459	0.286	0.247	0.0390
7.728667038	0.285	0.246	0.0390
7.730278902	0.285	0.246	0.0390
7.731914894	0.285	0.246	0.0390
4.010555927	0.951	0.912	0.0390
4.011308459	0.95	0.911	0.0390
4.011801977	0.95	0.911	0.0390
4.013030277	0.95	0.911	0.0390
4.014210712	0.95	0.911	0.0390
4.015902083	0.95	0.911	0.0390
4.018303002	0.95	0.911	0.0390
4.022363818	0.949	0.91	0.0390
4.023328572	0.949	0.91	0.0390
4.031012929	0.948	0.909	0.0390
4.032752261	0.948	0.909	0.0390
4.034589314	0.948	0.909	0.0390
4.048199768	0.947	0.908	0.0390
4.05003127	0.947	0.908	0.0390
4.052668053	0.947	0.908	0.0390
4.056012534	0.947	0.908	0.0390
4.058397272	0.947	0.908	0.0390
4.060661765	0.947	0.908	0.0390
4.08569368	0.944	0.905	0.0390
4.08703591	0.944	0.905	0.0390
4.088224052	0.944	0.905	0.0390
4.08853387	0.944	0.905	0.0390
4.091314841	0.944	0.905	0.0390
4.106478567	0.943	0.904	0.0390
4.124002563	0.941	0.902	0.0390
4.124646893	0.941	0.902	0.0390
4.125437063	0.941	0.902	0.0390
4.126429126	0.941	0.902	0.0390

4.127445552	0.941	0.902	0.0390
4.128469617	0.941	0.902	0.0390
4.129894008	0.94	0.901	0.0390
4.132256497	0.94	0.901	0.0390
4.135312199	0.94	0.901	0.0390
4.13723861	0.94	0.901	0.0390
4.138732959	0.94	0.901	0.0390
4.139659915	0.94	0.901	0.0390
4.14089839	0.94	0.901	0.0390
4.144907281	0.939	0.9	0.0390
4.145709592	0.939	0.9	0.0390
7.870395114	0.264	0.226	0.0380
7.875856164	0.263	0.225	0.0380
7.894736842	0.26	0.222	0.0380
7.896643783	0.26	0.222	0.0380
7.898855194	0.26	0.222	0.0380
7.901192735	0.26	0.222	0.0380
7.907736738	0.259	0.221	0.0380
7.909472247	0.259	0.221	0.0380
7.910920083	0.259	0.221	0.0380
7.912530735	0.258	0.22	0.0380
7.915167866	0.258	0.22	0.0380
7.919731544	0.257	0.219	0.0380
7.921038961	0.257	0.219	0.0380
7.923303112	0.257	0.219	0.0380
7.929802956	0.256	0.218	0.0380
7.945141139	0.253	0.215	0.0380
7.94891977	0.253	0.215	0.0380
7.951787789	0.253	0.215	0.0380
7.95365068	0.253	0.215	0.0380
7.962927648	0.251	0.213	0.0380
7.964594068	0.251	0.213	0.0380
8.096698802	0.23	0.192	0.0380
8.098375529	0.23	0.192	0.0380
8.099928686	0.229	0.191	0.0380
8.101727802	0.229	0.191	0.0380
8.104125194	0.229	0.191	0.0380
8.107084357	0.229	0.191	0.0380
7.709128499	0.288	0.25	0.0380
7.710894035	0.288	0.25	0.0380
7.712141354	0.288	0.25	0.0380
7.713352007	0.288	0.25	0.0380
7.717328119	0.287	0.249	0.0380

7.733854167	0.284	0.246	0.0380
7.735112028	0.284	0.246	0.0380
4.009325276	0.951	0.913	0.0380
4.009667476	0.951	0.913	0.0380
4.010158828	0.951	0.913	0.0380
4.010842099	0.95	0.912	0.0380
4.019619629	0.949	0.911	0.0380
4.020919816	0.949	0.911	0.0380
4.024412936	0.948	0.91	0.0380
4.027884739	0.948	0.91	0.0380
4.06396028	0.946	0.908	0.0380
4.065677313	0.946	0.908	0.0380
4.066459856	0.946	0.908	0.0380
4.067391128	0.946	0.908	0.0380
4.068782026	0.946	0.908	0.0380
4.070281951	0.946	0.908	0.0380
4.071480704	0.946	0.908	0.0380
4.082243561	0.945	0.907	0.0380
4.084138338	0.944	0.906	0.0380
4.084936448	0.944	0.906	0.0380
4.095172637	0.943	0.905	0.0380
4.097373099	0.943	0.905	0.0380
4.09954004	0.943	0.905	0.0380
4.10092151	0.943	0.905	0.0380
4.10244859	0.943	0.905	0.0380
4.103909914	0.943	0.905	0.0380
4.110204574	0.942	0.904	0.0380
4.111586692	0.942	0.904	0.0380
4.112980421	0.942	0.904	0.0380
4.115966387	0.942	0.904	0.0380
4.119008715	0.942	0.904	0.0380
4.120624746	0.942	0.904	0.0380
4.121045621	0.942	0.904	0.0380
4.123467045	0.941	0.903	0.0380
4.142829122	0.939	0.901	0.0380
4.144191625	0.939	0.901	0.0380
7.905101275	0.259	0.222	0.0370
7.955374917	0.252	0.215	0.0370
7.958465076	0.251	0.214	0.0370
7.959854995	0.251	0.214	0.0370
7.961154878	0.251	0.214	0.0370
7.965608987	0.25	0.213	0.0370
7.966567331	0.25	0.213	0.0370

7.967891484	0.25	0.213	0.0370
8.086670839	0.231	0.194	0.0370
8.109172164	0.228	0.191	0.0370
8.111062166	0.228	0.191	0.0370
8.113980823	0.227	0.19	0.0370
8.115348064	0.227	0.19	0.0370
8.11812139	0.227	0.19	0.0370
8.123106061	0.226	0.189	0.0370
3.995192308	0.952	0.915	0.0370
4.004405286	0.951	0.914	0.0370
4.009013581	0.951	0.914	0.0370
4.073119511	0.945	0.908	0.0370
4.074951729	0.945	0.908	0.0370
4.07595817	0.945	0.908	0.0370
4.078402223	0.945	0.908	0.0370
4.122217435	0.941	0.904	0.0370
7.956975429	0.251	0.215	0.0360
7.969337406	0.249	0.213	0.0360
7.970469652	0.249	0.213	0.0360
7.973414591	0.248	0.212	0.0360
7.974269877	0.248	0.212	0.0360
8.028683047	0.24	0.204	0.0360
8.029750055	0.24	0.204	0.0360
8.071858864	0.233	0.197	0.0360
8.073181615	0.233	0.197	0.0360
8.074304118	0.233	0.197	0.0360
8.078196638	0.232	0.196	0.0360
8.079735099	0.232	0.196	0.0360
8.083649082	0.231	0.195	0.0360
8.089919174	0.23	0.194	0.0360
8.093420574	0.23	0.194	0.0360
8.11254783	0.227	0.191	0.0360
8.120553061	0.226	0.19	0.0360
8.121008745	0.226	0.19	0.0360
8.127016129	0.225	0.189	0.0360
8.12926433	0.225	0.189	0.0360
8.03739225	0.239	0.203	0.0360
8.038595185	0.239	0.203	0.0360
8.039878113	0.239	0.203	0.0360
8.043535412	0.238	0.202	0.0360
8.045544897	0.238	0.202	0.0360
8.046426006	0.238	0.202	0.0360
8.065591398	0.235	0.199	0.0360

3.989727827	0.952	0.916	0.0360
7.971521232	0.248	0.213	0.0350
7.972500473	0.248	0.213	0.0350
7.975071834	0.247	0.212	0.0350
7.9793267	0.247	0.212	0.0350
7.983532123	0.247	0.212	0.0350
7.984183546	0.247	0.212	0.0350
7.984785375	0.247	0.212	0.0350
8.019253474	0.241	0.206	0.0350
8.022288446	0.241	0.206	0.0350
8.02445733	0.241	0.206	0.0350
8.030899547	0.239	0.204	0.0350
8.032141474	0.239	0.204	0.0350
8.034798351	0.239	0.204	0.0350
8.066946779	0.234	0.199	0.0350
8.068096204	0.234	0.199	0.0350
8.070197044	0.234	0.199	0.0350
8.075728619	0.232	0.197	0.0350
8.08109589	0.231	0.196	0.0350
8.131414868	0.224	0.189	0.0350
8.133830846	0.224	0.189	0.0350
8.143284859	0.222	0.187	0.0350
8.144436933	0.222	0.187	0.0350
8.145428327	0.222	0.187	0.0350
8.146921756	0.222	0.187	0.0350
8.14941654	0.221	0.186	0.0350
8.154937261	0.22	0.185	0.0350
8.156961553	0.219	0.184	0.0350
8.158947368	0.219	0.184	0.0350
8.160882353	0.219	0.184	0.0350
8.041249291	0.238	0.203	0.0350
8.047827744	0.237	0.202	0.0350
8.049547477	0.237	0.202	0.0350
8.05200287	0.237	0.202	0.0350
8.054623415	0.236	0.201	0.0350
8.056349206	0.236	0.201	0.0350
8.057348407	0.236	0.201	0.0350
8.058627725	0.236	0.201	0.0350
8.060153777	0.235	0.2	0.0350
8.061310782	0.235	0.2	0.0350
8.063265816	0.235	0.2	0.0350
3.942016807	0.957	0.922	0.0350
3.943650794	0.957	0.922	0.0350

3.944699286	0.957	0.922	0.0350
3.945450037	0.957	0.922	0.0350
3.946657183	0.957	0.922	0.0350
3.948043185	0.957	0.922	0.0350
3.95039761	0.956	0.921	0.0350
3.952052828	0.956	0.921	0.0350
3.954016913	0.956	0.921	0.0350
3.955294705	0.956	0.921	0.0350
3.957188645	0.956	0.921	0.0350
3.958333333	0.956	0.921	0.0350
3.961195809	0.955	0.92	0.0350
3.961808404	0.955	0.92	0.0350
3.962698896	0.955	0.92	0.0350
3.963548802	0.955	0.92	0.0350
3.965998844	0.954	0.919	0.0350
3.969051607	0.953	0.918	0.0350
3.970905932	0.953	0.918	0.0350
3.972282678	0.953	0.918	0.0350
3.973749069	0.953	0.918	0.0350
3.975399506	0.953	0.918	0.0350
3.976588171	0.953	0.918	0.0350
3.977272727	0.953	0.918	0.0350
3.982386916	0.952	0.917	0.0350
3.986008051	0.952	0.917	0.0350
3.988052909	0.952	0.917	0.0350
3.988917542	0.952	0.917	0.0350
7.985343069	0.246	0.212	0.0340
8.013941514	0.242	0.208	0.0340
8.01444514	0.242	0.208	0.0340
8.014986529	0.241	0.207	0.0340
8.015570096	0.241	0.207	0.0340
8.016200971	0.241	0.207	0.0340
8.026763337	0.24	0.206	0.0340
8.027689949	0.24	0.206	0.0340
8.13548716	0.223	0.189	0.0340
8.137288499	0.223	0.189	0.0340
8.138732959	0.223	0.189	0.0340
8.140280262	0.223	0.189	0.0340
8.141941392	0.223	0.189	0.0340
8.151775587	0.22	0.186	0.0340
8.153356198	0.22	0.186	0.0340
8.162515006	0.218	0.184	0.0340
8.163911134	0.218	0.184	0.0340

8.165611814	0.218	0.184	0.0340
8.167302799	0.218	0.184	0.0340
3.911967284	0.959	0.925	0.0340
3.915981199	0.959	0.925	0.0340
3.919258454	0.959	0.925	0.0340
3.938723518	0.957	0.923	0.0340
3.940285205	0.957	0.923	0.0340
3.949358974	0.956	0.922	0.0340
3.950088968	0.956	0.922	0.0340
3.959032438	0.955	0.921	0.0340
3.960385252	0.955	0.921	0.0340
3.964230503	0.954	0.92	0.0340
3.965007141	0.954	0.92	0.0340
3.967367208	0.953	0.919	0.0340
3.977883675	0.952	0.918	0.0340
3.979297063	0.952	0.918	0.0340
7.985861311	0.245	0.212	0.0330
7.986344146	0.245	0.212	0.0330
7.986795084	0.244	0.211	0.0330
7.987217185	0.244	0.211	0.0330
7.993710692	0.244	0.211	0.0330
8.006410256	0.242	0.209	0.0330
8.013032773	0.242	0.209	0.0330
8.013471832	0.242	0.209	0.0330
8.168476508	0.217	0.184	0.0330
8.174892828	0.216	0.183	0.0330
8.175429079	0.216	0.183	0.0330
8.175888152	0.216	0.183	0.0330
8.178959405	0.216	0.183	0.0330
8.184868822	0.215	0.182	0.0330
3.901412942	0.96	0.927	0.0330
3.921124962	0.958	0.925	0.0330
3.924516151	0.958	0.925	0.0330
3.927475898	0.958	0.925	0.0330
3.928571429	0.958	0.925	0.0330
3.929459844	0.958	0.925	0.0330
3.932387244	0.958	0.925	0.0330
3.936342593	0.957	0.924	0.0330
3.937661917	0.957	0.924	0.0330
3.937938466	0.957	0.924	0.0330
8.17180863	0.216	0.184	0.0320
8.18816263	0.214	0.182	0.0320
8.188691088	0.214	0.182	0.0320

8.190873731	0.214	0.182	0.0320
8.193159736	0.214	0.182	0.0320
8.193996416	0.214	0.182	0.0320
8.204126603	0.212	0.18	0.0320
8.206012378	0.212	0.18	0.0320
8.207925888	0.212	0.18	0.0320
8.210168669	0.211	0.179	0.0320
8.211651322	0.211	0.179	0.0320
8.213103122	0.211	0.179	0.0320
8.225317283	0.209	0.177	0.0320
8.228859793	0.209	0.177	0.0320
3.896392367	0.96	0.928	0.0320
3.897870491	0.96	0.928	0.0320
3.898693827	0.96	0.928	0.0320
3.903926452	0.959	0.927	0.0320
3.904435747	0.959	0.927	0.0320
3.905170652	0.959	0.927	0.0320
3.90710442	0.959	0.927	0.0320
3.909760265	0.959	0.927	0.0320
3.934805707	0.957	0.925	0.0320
8.194966583	0.213	0.182	0.0310
8.197123243	0.213	0.182	0.0310
8.200098306	0.213	0.182	0.0310
8.216731898	0.21	0.179	0.0310
8.222953216	0.209	0.178	0.0310
8.231030874	0.208	0.177	0.0310
8.231499917	0.208	0.177	0.0310
8.233500717	0.208	0.177	0.0310
8.235294118	0.208	0.177	0.0310
3.879143047	0.961	0.93	0.0310
3.881854996	0.961	0.93	0.0310
3.885195727	0.961	0.93	0.0310
3.887675885	0.961	0.93	0.0310
3.890145802	0.961	0.93	0.0310
3.891758754	0.96	0.929	0.0310
3.89205373	0.96	0.929	0.0310
3.893223169	0.96	0.929	0.0310
3.894483806	0.96	0.929	0.0310
3.89504284	0.96	0.929	0.0310
8.202281924	0.212	0.182	0.0300
8.219758533	0.209	0.179	0.0300
8.221280603	0.209	0.179	0.0300
8.237143914	0.207	0.177	0.0300

8.239215165	0.207	0.177	0.0300
8.243759026	0.206	0.176	0.0300
8.245514028	0.206	0.176	0.0300
8.247464215	0.206	0.176	0.0300
3.819660098	0.965	0.935	0.0300
3.821877065	0.965	0.935	0.0300
3.823529412	0.965	0.935	0.0300
3.824643494	0.965	0.935	0.0300
3.829046453	0.965	0.935	0.0300
3.834406658	0.965	0.935	0.0300
3.868486074	0.962	0.932	0.0300
3.877578328	0.961	0.931	0.0300
3.891514165	0.96	0.93	0.0300
8.23971831	0.206	0.177	0.0290
8.241621622	0.206	0.177	0.0290
8.249961717	0.205	0.176	0.0290
8.252858253	0.205	0.176	0.0290
8.254500906	0.205	0.176	0.0290
3.837430913	0.964	0.935	0.0290
3.838623199	0.964	0.935	0.0290
3.840335234	0.964	0.935	0.0290
3.842086221	0.964	0.935	0.0290
3.842579646	0.964	0.935	0.0290
3.842884983	0.964	0.935	0.0290
3.85013369	0.963	0.934	0.0290
3.86005712	0.962	0.933	0.0290
3.863222249	0.962	0.933	0.0290
3.866951955	0.962	0.933	0.0290
3.869197176	0.961	0.932	0.0290
3.870157238	0.961	0.932	0.0290
3.873809862	0.961	0.932	0.0290
3.784415256	0.968	0.94	0.0280
3.785178732	0.968	0.94	0.0280
3.802450117	0.966	0.938	0.0280
3.803248942	0.966	0.938	0.0280
3.806602396	0.966	0.938	0.0280
3.810839253	0.966	0.938	0.0280
3.812327348	0.966	0.938	0.0280
3.813764451	0.966	0.938	0.0280
8.277003882	0.202	0.174	0.0280
8.282416045	0.201	0.173	0.0280
8.255423755	0.204	0.176	0.0280
8.256694856	0.204	0.176	0.0280

8.257820137	0.204	0.176	0.0280
8.259467041	0.204	0.176	0.0280
8.29543497	0.199	0.171	0.0280
8.297084318	0.198	0.17	0.0280
3.81769306	0.965	0.937	0.0280
3.818638648	0.965	0.937	0.0280
3.844564526	0.963	0.935	0.0280
3.846153846	0.963	0.935	0.0280
3.847959552	0.963	0.935	0.0280
3.849882629	0.963	0.935	0.0280
3.850421046	0.962	0.934	0.0280
3.852661658	0.962	0.934	0.0280
3.856308312	0.962	0.934	0.0280
3.858758572	0.962	0.934	0.0280
3.759750177	0.969	0.942	0.0270
3.763163729	0.969	0.942	0.0270
3.763742902	0.969	0.942	0.0270
3.764738521	0.969	0.942	0.0270
3.76643698	0.969	0.942	0.0270
3.767778901	0.969	0.942	0.0270
3.771609207	0.968	0.941	0.0270
3.775681342	0.968	0.941	0.0270
3.778423773	0.968	0.941	0.0270
3.780726594	0.968	0.941	0.0270
3.783083602	0.968	0.941	0.0270
3.785859251	0.967	0.94	0.0270
3.787940726	0.967	0.94	0.0270
3.790341238	0.967	0.94	0.0270
3.79215612	0.967	0.94	0.0270
3.793873153	0.967	0.94	0.0270
3.79571715	0.967	0.94	0.0270
3.798297496	0.966	0.939	0.0270
3.799563319	0.966	0.939	0.0270
3.801041667	0.966	0.939	0.0270
3.815727011	0.965	0.938	0.0270
3.816814711	0.965	0.938	0.0270
8.262379227	0.203	0.176	0.0270
8.264175849	0.203	0.176	0.0270
8.265564738	0.203	0.176	0.0270
8.269196614	0.202	0.175	0.0270
8.272028993	0.202	0.175	0.0270
8.274621682	0.202	0.175	0.0270
8.27841711	0.201	0.174	0.0270

8.279969262	0.201	0.174	0.0270
8.284648188	0.2	0.173	0.0270
8.289405237	0.199	0.172	0.0270
8.291911084	0.199	0.172	0.0270
8.293628285	0.199	0.172	0.0270
8.298596034	0.197	0.17	0.0270
8.299986661	0.197	0.17	0.0270
8.303552604	0.197	0.17	0.0270
8.307637571	0.197	0.17	0.0270
8.309341342	0.196	0.169	0.0270
8.320889285	0.195	0.168	0.0270
8.321912986	0.194	0.167	0.0270
3.75	0.97	0.944	0.0260
3.756479467	0.969	0.943	0.0260
3.768874725	0.968	0.942	0.0260
3.797129899	0.966	0.94	0.0260
8.267191601	0.202	0.176	0.0260
8.286692759	0.199	0.173	0.0260
8.310773733	0.195	0.169	0.0260
8.315508021	0.195	0.169	0.0260
8.319969209	0.195	0.169	0.0260
8.321423102	0.194	0.168	0.0260
8.322364148	0.193	0.167	0.0260
8.327956989	0.193	0.167	0.0260
8.333333333	0.193	0.167	0.0260
8.3382643	0.192	0.166	0.0260
8.345075664	0.191	0.165	0.0260
8.345594201	0.191	0.165	0.0260
8.369582348	0.189	0.163	0.0260
3.744465898	0.97	0.945	0.0250
3.747427984	0.97	0.945	0.0250
3.753172589	0.969	0.944	0.0250
8.343376774	0.191	0.166	0.0250
8.343753663	0.191	0.166	0.0250
8.344159953	0.191	0.166	0.0250
8.344599224	0.191	0.166	0.0250
8.346160677	0.19	0.165	0.0250
8.346782065	0.19	0.165	0.0250
8.351185298	0.19	0.165	0.0250
8.355713771	0.19	0.165	0.0250
8.35665362	0.19	0.165	0.0250
8.357675906	0.19	0.165	0.0250
8.371231697	0.188	0.163	0.0250

8.373038382	0.188	0.163	0.0250
8.51982264	0.171	0.147	0.0240
8.361494415	0.189	0.165	0.0240
8.365396473	0.189	0.165	0.0240
8.366680005	0.189	0.165	0.0240
8.368070633	0.189	0.165	0.0240
8.37449187	0.187	0.163	0.0240
8.375811688	0.187	0.163	0.0240
8.377500878	0.187	0.163	0.0240
8.379330034	0.187	0.163	0.0240
8.381317316	0.187	0.163	0.0240
8.38817926	0.186	0.162	0.0240
8.390435068	0.186	0.162	0.0240
8.392884488	0.186	0.162	0.0240
8.487028559	0.176	0.152	0.0240
8.493563294	0.174	0.15	0.0240
8.495108253	0.174	0.15	0.0240
8.496486314	0.174	0.15	0.0240
8.496920926	0.174	0.15	0.0240
8.50170068	0.173	0.149	0.0240
8.581824918	0.164	0.14	0.0240
3.713127117	0.972	0.949	0.0230
3.733026114	0.971	0.948	0.0230
3.735087719	0.971	0.948	0.0230
3.738724096	0.97	0.947	0.0230
3.739483917	0.97	0.947	0.0230
3.741263728	0.97	0.947	0.0230
3.743002783	0.97	0.947	0.0230
3.743695669	0.97	0.947	0.0230
8.516054258	0.171	0.148	0.0230
8.517501018	0.171	0.148	0.0230
8.522308347	0.17	0.147	0.0230
8.524040048	0.17	0.147	0.0230
8.525115595	0.17	0.147	0.0230
8.526386404	0.17	0.147	0.0230
8.549002709	0.168	0.145	0.0230
8.550171479	0.168	0.145	0.0230
8.561867669	0.166	0.143	0.0230
8.562295752	0.166	0.143	0.0230
8.566964286	0.166	0.143	0.0230
8.575837743	0.165	0.142	0.0230
8.383484163	0.186	0.163	0.0230
8.384854276	0.186	0.163	0.0230

8.386094971	0.186	0.163	0.0230
8.395553574	0.185	0.162	0.0230
8.397191231	0.185	0.162	0.0230
8.398717949	0.185	0.162	0.0230
8.407444005	0.184	0.161	0.0230
8.483803553	0.176	0.153	0.0230
8.489887335	0.175	0.152	0.0230
8.491314765	0.175	0.152	0.0230
8.492607088	0.175	0.152	0.0230
8.498554913	0.173	0.15	0.0230
8.503669184	0.172	0.149	0.0230
8.506626889	0.172	0.149	0.0230
8.509977534	0.172	0.149	0.0230
8.512075906	0.172	0.149	0.0230
8.582383359	0.163	0.14	0.0230
8.583005249	0.163	0.14	0.0230
8.585784314	0.163	0.14	0.0230
8.588989442	0.163	0.14	0.0230
8.590173808	0.163	0.14	0.0230
3.71004233	0.972	0.95	0.0220
3.718388614	0.971	0.949	0.0220
3.722167244	0.971	0.949	0.0220
3.723947026	0.971	0.949	0.0220
3.724989996	0.971	0.949	0.0220
3.727609963	0.971	0.949	0.0220
3.730893573	0.971	0.949	0.0220
3.732388155	0.971	0.949	0.0220
3.737107921	0.97	0.948	0.0220
3.737845747	0.97	0.948	0.0220
8.514569257	0.171	0.149	0.0220
8.528271774	0.169	0.147	0.0220
8.530440148	0.169	0.147	0.0220
8.533250189	0.169	0.147	0.0220
8.536746693	0.169	0.147	0.0220
8.539303762	0.169	0.147	0.0220
8.540906326	0.168	0.146	0.0220
8.542985232	0.168	0.146	0.0220
8.544879171	0.168	0.146	0.0220
8.546920821	0.168	0.146	0.0220
8.551224388	0.167	0.145	0.0220
8.556165099	0.167	0.145	0.0220
8.56087857	0.167	0.145	0.0220
8.561397457	0.166	0.144	0.0220

8.580663997	0.164	0.142	0.0220
8.581320682	0.164	0.142	0.0220
8.401388889	0.184	0.162	0.0220
8.40428744	0.184	0.162	0.0220
8.411442006	0.183	0.161	0.0220
8.415529645	0.183	0.161	0.0220
8.417493853	0.182	0.16	0.0220
8.419387075	0.182	0.16	0.0220
8.422647528	0.182	0.16	0.0220
8.424449979	0.182	0.16	0.0220
8.429401993	0.181	0.159	0.0220
8.430802554	0.181	0.159	0.0220
8.434436275	0.181	0.159	0.0220
8.456129096	0.179	0.157	0.0220
8.45895715	0.179	0.157	0.0220
8.477541031	0.177	0.155	0.0220
8.480506329	0.176	0.154	0.0220
8.481885639	0.176	0.154	0.0220
8.591076661	0.162	0.14	0.0220
8.592070944	0.162	0.14	0.0220
3.709298804	0.972	0.951	0.0210
8.424927192	0.181	0.16	0.0210
8.426434903	0.181	0.16	0.0210
8.428122192	0.181	0.16	0.0210
8.440306886	0.18	0.159	0.0210
8.443779108	0.18	0.159	0.0210
8.447028424	0.18	0.159	0.0210
8.450158314	0.18	0.159	0.0210
8.451158564	0.18	0.159	0.0210
8.453448728	0.179	0.158	0.0210
8.455583453	0.179	0.158	0.0210
8.464102564	0.178	0.157	0.0210
8.466909976	0.178	0.157	0.0210
8.46887076	0.178	0.157	0.0210
8.474521707	0.177	0.156	0.0210
8.479130435	0.176	0.155	0.0210
8.593171296	0.161	0.14	0.0210
8.594395661	0.161	0.14	0.0210
8.596883718	0.161	0.14	0.0210
8.599363057	0.161	0.14	0.0210
3.705733055	0.972	0.952	0.0200
3.706599713	0.972	0.952	0.0200
3.70811863	0.972	0.952	0.0200

8.471405229	0.177	0.157	0.0200
8.600699301	0.16	0.14	0.0200
8.602169889	0.16	0.14	0.0200
8.607076157	0.159	0.139	0.0200
8.60843323	0.159	0.139	0.0200
8.610191317	0.159	0.139	0.0200
8.612124899	0.159	0.139	0.0200
8.613798259	0.159	0.139	0.0200
8.614921223	0.159	0.139	0.0200
8.626702589	0.157	0.137	0.0200
3.636363636	0.976	0.957	0.0190
3.657462125	0.974	0.955	0.0190
3.6591652	0.974	0.955	0.0190
3.661580076	0.974	0.955	0.0190
3.663981305	0.974	0.955	0.0190
3.666468991	0.974	0.955	0.0190
3.669613892	0.974	0.955	0.0190
3.675444596	0.973	0.954	0.0190
3.677857936	0.973	0.954	0.0190
3.680113439	0.973	0.954	0.0190
3.681286817	0.973	0.954	0.0190
3.681705111	0.973	0.954	0.0190
3.683014354	0.973	0.954	0.0190
3.685539607	0.973	0.954	0.0190
3.702502861	0.972	0.953	0.0190
8.604749277	0.159	0.14	0.0190
8.615868408	0.158	0.139	0.0190
8.61711919	0.158	0.139	0.0190
8.619555354	0.157	0.138	0.0190
8.62193903	0.157	0.138	0.0190
8.624571302	0.157	0.138	0.0190
8.628241619	0.156	0.137	0.0190
8.629584622	0.156	0.137	0.0190
8.630544684	0.156	0.137	0.0190
8.632022953	0.156	0.137	0.0190
8.633316949	0.156	0.137	0.0190
8.638181818	0.155	0.136	0.0190
8.641428571	0.155	0.136	0.0190
8.644736842	0.155	0.136	0.0190
3.611160771	0.978	0.96	0.0180
3.619910115	0.977	0.959	0.0180
3.622293791	0.977	0.959	0.0180
3.624094203	0.977	0.959	0.0180

3.625686813	0.977	0.959	0.0180
3.627424991	0.976	0.958	0.0180
3.629240113	0.976	0.958	0.0180
3.630762815	0.976	0.958	0.0180
3.631802721	0.976	0.958	0.0180
3.634508349	0.976	0.958	0.0180
3.638458316	0.975	0.957	0.0180
3.640664847	0.975	0.957	0.0180
3.641197598	0.975	0.957	0.0180
3.642843147	0.975	0.957	0.0180
3.644256121	0.975	0.957	0.0180
3.644652129	0.975	0.957	0.0180
3.645090005	0.975	0.957	0.0180
3.648926237	0.974	0.956	0.0180
3.653590658	0.974	0.956	0.0180
3.67265234	0.973	0.955	0.0180
3.687878788	0.972	0.954	0.0180
3.690598291	0.972	0.954	0.0180
3.693979933	0.972	0.954	0.0180
3.69678442	0.972	0.954	0.0180
3.698273402	0.972	0.954	0.0180
3.699026051	0.972	0.954	0.0180
8.618153616	0.157	0.139	0.0180
8.634952005	0.155	0.137	0.0180
8.647632595	0.154	0.136	0.0180
8.650565459	0.154	0.136	0.0180
8.658127353	0.153	0.135	0.0180
8.659976954	0.153	0.135	0.0180
8.661694577	0.153	0.135	0.0180
3.609225875	0.978	0.961	0.0170
3.612734335	0.977	0.96	0.0170
3.615662139	0.977	0.96	0.0170
3.626658316	0.976	0.959	0.0170
3.645864518	0.974	0.957	0.0170
3.646733832	0.974	0.957	0.0170
8.655100195	0.153	0.136	0.0170
8.664319249	0.152	0.135	0.0170
8.668997669	0.152	0.135	0.0170
8.685170957	0.15	0.133	0.0170
8.68921954	0.15	0.133	0.0170
8.693979933	0.149	0.132	0.0170
8.697141155	0.149	0.132	0.0170
8.698908564	0.149	0.132	0.0170

8.700738534	0.149	0.132	0.0170
3.598307845	0.978	0.962	0.0160
3.601568006	0.978	0.962	0.0160
3.60560427	0.978	0.962	0.0160
3.6074024	0.978	0.962	0.0160
3.608471537	0.978	0.962	0.0160
8.671601836	0.151	0.135	0.0160
8.673685844	0.151	0.135	0.0160
8.675983638	0.151	0.135	0.0160
8.677078269	0.151	0.135	0.0160
8.679120753	0.151	0.135	0.0160
8.681363049	0.151	0.135	0.0160
8.682402637	0.15	0.134	0.0160
8.683422628	0.15	0.134	0.0160
8.703663024	0.148	0.132	0.0160
8.706259482	0.148	0.132	0.0160
3.549212098	0.98	0.965	0.0150
3.55260603	0.98	0.965	0.0150
3.570665673	0.979	0.964	0.0150
3.579786302	0.979	0.964	0.0150
3.586779309	0.979	0.964	0.0150
3.591408591	0.979	0.964	0.0150
3.59632742	0.978	0.963	0.0150
3.59800722	0.978	0.963	0.0150
8.708580206	0.147	0.132	0.0150
8.710899316	0.147	0.132	0.0150
8.717082467	0.146	0.131	0.0150
8.718974359	0.146	0.131	0.0150
8.720902256	0.145	0.13	0.0150
8.722604383	0.145	0.13	0.0150
8.724118235	0.145	0.13	0.0150
8.725473432	0.145	0.13	0.0150
8.72813669	0.145	0.13	0.0150
8.730751007	0.145	0.13	0.0150
3.543167318	0.98	0.966	0.0140
3.554427524	0.979	0.965	0.0140
3.559386973	0.979	0.965	0.0140
3.563787413	0.979	0.965	0.0140
3.564965103	0.979	0.965	0.0140
3.593922417	0.978	0.964	0.0140
8.808735415	0.137	0.123	0.0140
8.813001813	0.136	0.122	0.0140
8.815302144	0.136	0.122	0.0140

8.817616959	0.136	0.122	0.0140
8.825557809	0.135	0.121	0.0140
8.827855603	0.135	0.121	0.0140
8.837163058	0.134	0.12	0.0140
8.840842442	0.134	0.12	0.0140
8.844564526	0.134	0.12	0.0140
8.713203463	0.146	0.132	0.0140
8.715250965	0.146	0.132	0.0140
8.731868825	0.144	0.13	0.0140
8.73286385	0.144	0.13	0.0140
8.736745407	0.144	0.13	0.0140
8.740449111	0.144	0.13	0.0140
3.503729393	0.981	0.968	0.0130
3.511371289	0.981	0.968	0.0130
3.518570506	0.981	0.968	0.0130
3.527863777	0.98	0.967	0.0130
3.53101023	0.98	0.967	0.0130
3.535172272	0.98	0.967	0.0130
3.538098694	0.98	0.967	0.0130
3.539142274	0.98	0.967	0.0130
8.768673356	0.14	0.127	0.0130
8.771712159	0.14	0.127	0.0130
8.774851876	0.14	0.127	0.0130
8.777802186	0.139	0.126	0.0130
8.77955688	0.139	0.126	0.0130
8.781269543	0.139	0.126	0.0130
8.782917533	0.139	0.126	0.0130
8.786796537	0.138	0.125	0.0130
8.788843853	0.138	0.125	0.0130
8.792067579	0.138	0.125	0.0130
8.797163121	0.138	0.125	0.0130
8.801408451	0.138	0.125	0.0130
8.806958585	0.137	0.124	0.0130
8.81035631	0.136	0.123	0.0130
8.821486928	0.135	0.122	0.0130
8.823529412	0.135	0.122	0.0130
8.830120894	0.134	0.121	0.0130
8.833866613	0.134	0.121	0.0130
8.847537355	0.133	0.12	0.0130
8.850136107	0.133	0.12	0.0130
8.851905184	0.133	0.12	0.0130
8.852981101	0.133	0.12	0.0130
8.855323021	0.133	0.12	0.0130

8.740999741	0.143	0.13	0.0130
8.741698553	0.143	0.13	0.0130
8.746069182	0.143	0.13	0.0130
8.765832932	0.141	0.128	0.0130
8.766678527	0.141	0.128	0.0130
8.767619615	0.141	0.128	0.0130
3.488977713	0.981	0.969	0.0120
3.493425546	0.981	0.969	0.0120
3.499785954	0.981	0.969	0.0120
3.519980618	0.98	0.968	0.0120
3.520767462	0.98	0.968	0.0120
3.523721275	0.98	0.968	0.0120
8.776244311	0.139	0.127	0.0120
8.784749035	0.138	0.126	0.0120
8.804393525	0.137	0.125	0.0120
8.86038961	0.132	0.12	0.0120
8.864442295	0.132	0.12	0.0120
8.865957447	0.132	0.12	0.0120
8.867857143	0.132	0.12	0.0120
8.754084967	0.142	0.13	0.0120
8.758395312	0.142	0.13	0.0120
8.758872389	0.142	0.13	0.0120
8.762197902	0.141	0.129	0.0120
8.765068991	0.141	0.129	0.0120
3.408085278	0.985	0.974	0.0110
3.410427807	0.985	0.974	0.0110
3.419069029	0.984	0.973	0.0110
3.422064777	0.984	0.973	0.0110
3.423867229	0.984	0.973	0.0110
3.426614481	0.984	0.973	0.0110
3.430703625	0.984	0.973	0.0110
3.433156108	0.984	0.973	0.0110
3.444917152	0.983	0.972	0.0110
3.45224853	0.983	0.972	0.0110
3.456834003	0.983	0.972	0.0110
3.457695367	0.983	0.972	0.0110
3.48777579	0.981	0.97	0.0110
8.870614035	0.131	0.12	0.0110
8.872709944	0.131	0.12	0.0110
8.876619718	0.131	0.12	0.0110
8.880298507	0.131	0.12	0.0110
8.881348914	0.13	0.119	0.0110
8.885233918	0.13	0.119	0.0110

8.759407005	0.141	0.13	0.0110
3.379536371	0.986	0.976	0.0100
3.381468738	0.986	0.976	0.0100
3.382363545	0.986	0.976	0.0100
3.393125263	0.985	0.975	0.0100
3.398969452	0.985	0.975	0.0100
3.403944681	0.985	0.975	0.0100
3.412469179	0.984	0.974	0.0100
3.41512954	0.984	0.974	0.0100
3.434529608	0.983	0.973	0.0100
3.435740129	0.983	0.973	0.0100
3.438378825	0.983	0.973	0.0100
3.441209328	0.983	0.973	0.0100
3.458701692	0.982	0.972	0.0100
3.46049896	0.982	0.972	0.0100
3.462612806	0.982	0.972	0.0100
3.465511917	0.982	0.972	0.0100
3.468362219	0.982	0.972	0.0100
3.469853695	0.982	0.972	0.0100
3.471270928	0.982	0.972	0.0100
3.47489408	0.981	0.971	0.0100
3.477731618	0.981	0.971	0.0100
3.482350982	0.981	0.971	0.0100
3.485281385	0.981	0.971	0.0100
3.486446886	0.981	0.971	0.0100
8.880857948	0.13	0.12	0.0100
8.892720307	0.129	0.119	0.0100
8.896805274	0.129	0.119	0.0100
8.90003841	0.128	0.118	0.0100
8.902832415	0.128	0.118	0.0100
8.903667698	0.128	0.118	0.0100
8.904609539	0.128	0.118	0.0100
8.921970116	0.126	0.116	0.0100
8.925897166	0.125	0.115	0.0100
8.927372963	0.125	0.115	0.0100
8.929934569	0.125	0.115	0.0100
8.949208686	0.123	0.113	0.0100
8.953136416	0.123	0.113	0.0100
8.956778607	0.123	0.113	0.0100
8.959166667	0.123	0.113	0.0100
8.961481481	0.123	0.113	0.0100
3.350126904	0.987	0.978	0.0090
3.350802579	0.987	0.978	0.0090

3.368085566	0.986	0.977	0.0090
3.371901195	0.986	0.977	0.0090
3.373750735	0.986	0.977	0.0090
3.376131687	0.986	0.977	0.0090
3.383271612	0.985	0.976	0.0090
3.384642709	0.985	0.976	0.0090
3.386461773	0.985	0.976	0.0090
3.387537458	0.985	0.976	0.0090
3.38854974	0.985	0.976	0.0090
3.473200313	0.981	0.972	0.0090
8.897348309	0.128	0.119	0.0090
8.905679745	0.127	0.118	0.0090
8.909646739	0.127	0.118	0.0090
8.91388608	0.127	0.118	0.0090
8.918058186	0.126	0.117	0.0090
8.919891114	0.126	0.117	0.0090
8.923563778	0.125	0.116	0.0090
8.924835234	0.125	0.116	0.0090
8.93286197	0.124	0.115	0.0090
8.935298221	0.124	0.115	0.0090
8.937782076	0.124	0.115	0.0090
8.941241685	0.124	0.115	0.0090
8.943880675	0.123	0.114	0.0090
8.9457339	0.123	0.114	0.0090
8.964240102	0.122	0.113	0.0090
8.966885605	0.122	0.113	0.0090
8.972315961	0.122	0.113	0.0090
8.977240071	0.122	0.113	0.0090
8.985996866	0.121	0.112	0.0090
8.986914129	0.121	0.112	0.0090
3.341666667	0.987	0.979	0.0080
3.352146264	0.986	0.978	0.0080
3.353117295	0.986	0.978	0.0080
3.358062636	0.986	0.978	0.0080
3.363234111	0.986	0.978	0.0080
3.36451049	0.986	0.978	0.0080
8.915963067	0.126	0.118	0.0080
8.943375701	0.123	0.115	0.0080
8.978847013	0.121	0.113	0.0080
8.981321342	0.121	0.113	0.0080
8.984279047	0.121	0.113	0.0080
8.989794917	0.12	0.112	0.0080
8.992526909	0.12	0.112	0.0080

8.993047173	0.12	0.112	0.0080
9.010859047	0.118	0.11	0.0080
9.014618011	0.118	0.11	0.0080
9.103962944	0.108	0.1	0.0080
9.105084334	0.108	0.1	0.0080
9.107640049	0.108	0.1	0.0080
9.122854606	0.107	0.099	0.0080
9.125535859	0.107	0.099	0.0080
9.127250453	0.107	0.099	0.0080
3.295771965	0.988	0.981	0.0070
3.297259352	0.988	0.981	0.0070
3.298699196	0.988	0.981	0.0070
3.300202239	0.988	0.981	0.0070
3.302700082	0.988	0.981	0.0070
3.304156292	0.988	0.981	0.0070
3.317223913	0.987	0.98	0.0070
3.318675584	0.987	0.98	0.0070
3.326149425	0.987	0.98	0.0070
3.333333333	0.987	0.98	0.0070
9.018065268	0.117	0.11	0.0070
9.022684632	0.117	0.11	0.0070
9.025182135	0.117	0.11	0.0070
9.027912386	0.117	0.11	0.0070
9.031054405	0.116	0.109	0.0070
9.033370412	0.116	0.109	0.0070
9.035313668	0.116	0.109	0.0070
9.036590808	0.116	0.109	0.0070
9.042606769	0.115	0.108	0.0070
9.052574472	0.114	0.107	0.0070
9.054586082	0.114	0.107	0.0070
9.063624101	0.113	0.106	0.0070
9.065707434	0.112	0.105	0.0070
9.067231638	0.112	0.105	0.0070
9.068782026	0.112	0.105	0.0070
9.070598007	0.112	0.105	0.0070
9.072138127	0.112	0.105	0.0070
9.074238967	0.112	0.105	0.0070
9.076276665	0.112	0.105	0.0070
9.078480776	0.111	0.104	0.0070
9.081458417	0.111	0.104	0.0070
9.084737181	0.11	0.103	0.0070
9.087938206	0.11	0.103	0.0070
9.090909091	0.11	0.103	0.0070

9.097483292	0.109	0.102	0.0070
8.996644295	0.119	0.112	0.0070
9.007362893	0.118	0.111	0.0070
9.100462379	0.108	0.101	0.0070
9.103006189	0.108	0.101	0.0070
9.110350076	0.107	0.1	0.0070
9.114379085	0.107	0.1	0.0070
9.11963434	0.107	0.1	0.0070
9.128975781	0.106	0.099	0.0070
9.133562715	0.106	0.099	0.0070
9.138657824	0.105	0.098	0.0070
9.141741071	0.105	0.098	0.0070
9.145071982	0.105	0.098	0.0070
9.164171657	0.103	0.096	0.0070
3.291871417	0.988	0.982	0.0060
3.295454545	0.988	0.982	0.0060
3.309477284	0.987	0.981	0.0060
3.315334459	0.987	0.981	0.0060
9.038518519	0.115	0.109	0.0060
9.040547945	0.115	0.109	0.0060
9.045868347	0.114	0.108	0.0060
9.04912598	0.114	0.108	0.0060
9.050863901	0.114	0.108	0.0060
9.056544562	0.113	0.107	0.0060
9.060235507	0.113	0.107	0.0060
9.077468631	0.111	0.105	0.0060
9.084238254	0.11	0.104	0.0060
9.094065657	0.109	0.103	0.0060
9.098052508	0.108	0.102	0.0060
9.452589897	0.082	0.076	0.0060
9.455244671	0.082	0.076	0.0060
9.464368052	0.081	0.075	0.0060
9.466157761	0.081	0.075	0.0060
9.470175439	0.081	0.075	0.0060
9.473684211	0.081	0.075	0.0060
9.490902359	0.079	0.073	0.0060
9.49457825	0.079	0.073	0.0060
9.003311258	0.118	0.112	0.0060
9.006857358	0.118	0.112	0.0060
9.148111496	0.104	0.098	0.0060
9.149631484	0.104	0.098	0.0060
9.151434585	0.104	0.098	0.0060
9.153194263	0.104	0.098	0.0060

9.15761746	0.103	0.097	0.0060
9.160572252	0.103	0.097	0.0060
9.161257904	0.103	0.097	0.0060
9.166666667	0.102	0.096	0.0060
9.16954023	0.102	0.096	0.0060
9.172673062	0.102	0.096	0.0060
3.287336632	0.988	0.983	0.0050
9.173243025	0.101	0.096	0.0050
9.175817955	0.101	0.096	0.0050
9.204623879	0.099	0.094	0.0050
9.216517857	0.098	0.093	0.0050
9.219304078	0.098	0.093	0.0050
9.222332179	0.098	0.093	0.0050
9.225771294	0.097	0.092	0.0050
9.228479853	0.097	0.092	0.0050
9.230769231	0.097	0.092	0.0050
9.25524405	0.094	0.089	0.0050
9.259250862	0.094	0.089	0.0050
9.263225424	0.094	0.089	0.0050
9.265686275	0.094	0.089	0.0050
9.267479675	0.094	0.089	0.0050
9.269182838	0.094	0.089	0.0050
9.275839054	0.093	0.088	0.0050
9.278157895	0.093	0.088	0.0050
9.28028777	0.093	0.088	0.0050
9.390602434	0.085	0.08	0.0050
9.395194066	0.085	0.08	0.0050
9.400740658	0.085	0.08	0.0050
9.404882368	0.085	0.08	0.0050
9.422375143	0.084	0.079	0.0050
9.429475588	0.083	0.078	0.0050
9.43150193	0.083	0.078	0.0050
9.434621916	0.083	0.078	0.0050
9.438589579	0.083	0.078	0.0050
9.44854738	0.082	0.077	0.0050
9.450436846	0.082	0.077	0.0050
9.459451401	0.081	0.076	0.0050
9.462312855	0.081	0.076	0.0050
9.475648075	0.08	0.075	0.0050
9.479546711	0.08	0.075	0.0050
9.485266288	0.079	0.074	0.0050
9.498201439	0.078	0.073	0.0050
9.501552795	0.078	0.073	0.0050

9.503325845	0.078	0.073	0.0050
9.505294176	0.078	0.073	0.0050
9.515565134	0.077	0.072	0.0050
9.51862069	0.077	0.072	0.0050
9.521904762	0.077	0.072	0.0050
9.531706871	0.076	0.071	0.0050
9.534293792	0.076	0.071	0.0050
9.53744269	0.076	0.071	0.0050
9.540223136	0.076	0.071	0.0050
9.649118487	0.068	0.063	0.0050
9.651563714	0.068	0.063	0.0050
9.653975096	0.068	0.063	0.0050
9.69348209	0.066	0.061	0.0050
9.695813093	0.066	0.061	0.0050
9.698108909	0.065	0.06	0.0050
9.700370329	0.065	0.06	0.0050
9.702598121	0.065	0.06	0.0050
9.778594771	0.06	0.055	0.0050
9.154387866	0.103	0.098	0.0050
9.379577653	0.086	0.081	0.0050
9.381703303	0.086	0.081	0.0050
9.385658373	0.086	0.081	0.0050
9.598657718	0.072	0.067	0.0050
3.241968602	0.99	0.986	0.0040
3.248183914	0.99	0.986	0.0040
3.255265467	0.989	0.985	0.0040
3.262148222	0.989	0.985	0.0040
3.26885663	0.989	0.985	0.0040
3.270979021	0.989	0.985	0.0040
3.283817488	0.988	0.984	0.0040
9.178593335	0.1	0.096	0.0040
9.182144831	0.1	0.096	0.0040
9.186088528	0.1	0.096	0.0040
9.187245935	0.1	0.096	0.0040
9.189338235	0.1	0.096	0.0040
9.192362429	0.1	0.096	0.0040
9.19408963	0.1	0.096	0.0040
9.195855582	0.099	0.095	0.0040
9.198540146	0.099	0.095	0.0040
9.201449275	0.099	0.095	0.0040
9.208437761	0.098	0.094	0.0040
9.212406015	0.098	0.094	0.0040
9.225079157	0.097	0.093	0.0040

9.233440171	0.096	0.092	0.0040
9.236376166	0.096	0.092	0.0040
9.239010266	0.096	0.092	0.0040
9.24906015	0.095	0.091	0.0040
9.252716012	0.094	0.09	0.0040
9.270798086	0.093	0.089	0.0040
9.273442749	0.093	0.089	0.0040
9.285677841	0.092	0.088	0.0040
9.291059362	0.092	0.088	0.0040
9.292408422	0.092	0.088	0.0040
9.297088781	0.092	0.088	0.0040
9.301512441	0.092	0.088	0.0040
9.318954855	0.09	0.086	0.0040
9.38853404	0.085	0.081	0.0040
9.407093534	0.084	0.08	0.0040
9.409586057	0.084	0.08	0.0040
9.41391155	0.084	0.08	0.0040
9.418174125	0.084	0.08	0.0040
9.42534497	0.083	0.079	0.0040
9.427400468	0.083	0.079	0.0040
9.442501943	0.082	0.078	0.0040
9.446360153	0.082	0.078	0.0040
9.508765882	0.077	0.073	0.0040
9.511342316	0.077	0.073	0.0040
9.513042005	0.077	0.073	0.0040
9.527005433	0.076	0.072	0.0040
9.530725671	0.076	0.072	0.0040
9.543719639	0.075	0.071	0.0040
9.547163363	0.075	0.071	0.0040
9.619766366	0.07	0.066	0.0040
9.622636136	0.07	0.066	0.0040
9.637662133	0.069	0.065	0.0040
9.646638697	0.068	0.064	0.0040
9.65635333	0.067	0.063	0.0040
9.658699096	0.067	0.063	0.0040
9.663265306	0.067	0.063	0.0040
9.668044077	0.067	0.063	0.0040
9.670776318	0.067	0.063	0.0040
9.678709677	0.066	0.062	0.0040
9.681269841	0.066	0.062	0.0040
9.685019841	0.066	0.062	0.0040
9.68871124	0.066	0.062	0.0040
9.691115086	0.066	0.062	0.0040

9.704793028	0.064	0.06	0.0040
9.706955775	0.063	0.059	0.0040
9.709087062	0.063	0.059	0.0040
9.711187572	0.063	0.059	0.0040
9.714271136	0.062	0.058	0.0040
9.718295889	0.062	0.058	0.0040
9.721250971	0.062	0.058	0.0040
9.723180077	0.062	0.058	0.0040
9.725082664	0.062	0.058	0.0040
9.727878563	0.062	0.058	0.0040
9.731531532	0.062	0.058	0.0040
9.774423338	0.06	0.056	0.0040
9.77694859	0.06	0.056	0.0040
9.780216831	0.059	0.055	0.0040
9.781815297	0.059	0.055	0.0040
9.785670545	0.059	0.055	0.0040
9.789471092	0.059	0.055	0.0040
9.358058608	0.087	0.083	0.0040
9.359487179	0.087	0.083	0.0040
9.362539683	0.087	0.083	0.0040
9.367579053	0.087	0.083	0.0040
9.370354055	0.087	0.083	0.0040
9.372814685	0.087	0.083	0.0040
9.377155172	0.086	0.082	0.0040
9.550555493	0.074	0.07	0.0040
9.555531168	0.074	0.07	0.0040
9.560433663	0.074	0.07	0.0040
9.563630593	0.074	0.07	0.0040
9.575966437	0.073	0.069	0.0040
9.578942185	0.073	0.069	0.0040
9.581876457	0.073	0.069	0.0040
9.584770115	0.073	0.069	0.0040
9.595684394	0.072	0.068	0.0040
9.597044815	0.072	0.068	0.0040
9.601587302	0.071	0.067	0.0040
9.604218881	0.071	0.067	0.0040
9.605781185	0.071	0.067	0.0040
9.607071175	0.071	0.067	0.0040
9.608609069	0.071	0.067	0.0040
3.118270618	0.992	0.989	0.0030
3.126352665	0.992	0.989	0.0030
3.135151056	0.992	0.989	0.0030
3.140056022	0.992	0.989	0.0030

3.227076454	0.99	0.987	0.0030
3.229557844	0.99	0.987	0.0030
3.231546232	0.99	0.987	0.0030
3.233182893	0.99	0.987	0.0030
3.235278538	0.99	0.987	0.0030
3.250088337	0.989	0.986	0.0030
3.252974254	0.989	0.986	0.0030
3.276148583	0.988	0.985	0.0030
3.280409946	0.988	0.985	0.0030
9.241901776	0.095	0.092	0.0030
9.244499792	0.095	0.092	0.0030
9.247347822	0.095	0.092	0.0030
9.25085034	0.094	0.091	0.0030
9.305008945	0.091	0.088	0.0030
9.309018568	0.091	0.088	0.0030
9.311660963	0.091	0.088	0.0030
9.316625156	0.09	0.087	0.0030
9.321518081	0.089	0.086	0.0030
9.32583324	0.089	0.086	0.0030
9.330845771	0.089	0.086	0.0030
9.61088905	0.07	0.067	0.0030
9.613893858	0.07	0.067	0.0030
9.616852613	0.07	0.067	0.0030
9.624530075	0.069	0.066	0.0030
9.627314815	0.069	0.066	0.0030
9.630991285	0.069	0.066	0.0030
9.633694719	0.069	0.066	0.0030
9.641572456	0.068	0.065	0.0030
9.644123607	0.068	0.065	0.0030
9.674775251	0.066	0.063	0.0030
9.791656619	0.058	0.055	0.0030
9.795880583	0.058	0.055	0.0030
9.815295246	0.058	0.055	0.0030
9.832633053	0.058	0.055	0.0030
9.834022039	0.058	0.055	0.0030
9.836054559	0.058	0.055	0.0030
9.839334108	0.058	0.055	0.0030
9.734216336	0.061	0.058	0.0030
9.735970722	0.061	0.058	0.0030
9.738550923	0.061	0.058	0.0030
9.75012987	0.061	0.058	0.0030
9.760952381	0.06	0.057	0.0030
9.764673311	0.06	0.057	0.0030

9.770084567	0.06	0.057	0.0030
9.344735461	0.088	0.085	0.0030
9.347115658	0.088	0.085	0.0030
9.355990783	0.087	0.084	0.0030
9.569842738	0.073	0.07	0.0030
9.588185415	0.072	0.069	0.0030
9.591829935	0.072	0.069	0.0030
9.594045265	0.072	0.069	0.0030
3.006001551	0.994	0.992	0.0020
3.113465482	0.992	0.99	0.0020
3.144198524	0.991	0.989	0.0020
3.146045815	0.991	0.989	0.0020
3.15105364	0.991	0.989	0.0020
3.157777778	0.991	0.989	0.0020
3.161027668	0.991	0.989	0.0020
3.162224249	0.991	0.989	0.0020
3.167017477	0.991	0.989	0.0020
3.190915451	0.99	0.988	0.0020
3.197363799	0.99	0.988	0.0020
3.207230484	0.99	0.988	0.0020
3.219610449	0.99	0.988	0.0020
3.224473474	0.99	0.988	0.0020
9.314022796	0.09	0.088	0.0020
9.335540839	0.088	0.086	0.0020
9.337991819	0.088	0.086	0.0020
9.340650494	0.088	0.086	0.0020
9.841894763	0.057	0.055	0.0020
9.843134843	0.057	0.055	0.0020
9.84435562	0.057	0.055	0.0020
9.845557543	0.057	0.055	0.0020
12.10158263	0.004	0.002	0.0020
12.10896961	0.004	0.002	0.0020
12.11694409	0.004	0.002	0.0020
12.2009857	0.003	0.001	0.0020
12.20405712	0.003	0.001	0.0020
12.2273622	0.003	0.001	0.0020
9.351332398	0.087	0.085	0.0020
1.96045354	1	0.999	0.0010
2.999417294	0.994	0.993	0.0010
3.008861439	0.993	0.992	0.0010
3.011512297	0.993	0.992	0.0010
3.016311247	0.993	0.992	0.0010
3.025738094	0.993	0.992	0.0010

3.035444947	0.993	0.992	0.0010
3.043672014	0.993	0.992	0.0010
3.049064171	0.993	0.992	0.0010
3.051315789	0.993	0.992	0.0010
3.058979106	0.993	0.992	0.0010
3.080128205	0.992	0.991	0.0010
3.084219858	0.992	0.991	0.0010
3.090172239	0.992	0.991	0.0010
3.102642971	0.992	0.991	0.0010
3.178562831	0.99	0.989	0.0010
9.846741045	0.056	0.055	0.0010
9.847906546	0.056	0.055	0.0010
9.849054454	0.056	0.055	0.0010
9.850185164	0.056	0.055	0.0010
9.868329868	0.054	0.053	0.0010
10.22557666	0.04	0.039	0.0010
11.38071349	0.01	0.009	0.0010
11.39543269	0.01	0.009	0.0010
11.41741071	0.01	0.009	0.0010
11.4389234	0.01	0.009	0.0010
11.44982852	0.01	0.009	0.0010
11.45168657	0.01	0.009	0.0010
11.52475845	0.008	0.007	0.0010
11.53594771	0.008	0.007	0.0010
11.55330882	0.008	0.007	0.0010
11.57490079	0.008	0.007	0.0010
11.64766718	0.007	0.006	0.0010
11.94356852	0.005	0.004	0.0010
11.95818283	0.005	0.004	0.0010
12.09733815	0.004	0.003	0.0010
12.12957158	0.003	0.002	0.0010
12.14588859	0.003	0.002	0.0010
12.15692308	0.003	0.002	0.0010
12.16208955	0.003	0.002	0.0010
12.1805744	0.003	0.002	0.0010
12.19777563	0.003	0.002	0.0010
12.25403226	0.002	0.001	0.0010
12.26539589	0.002	0.001	0.0010
12.29578393	0.002	0.001	0.0010
12.32483382	0.002	0.001	0.0010
12.33329675	0.002	0.001	0.0010
11.4980315	0.009	0.008	0.0010
11.50396825	0.009	0.008	0.0010

11.51154401	0.009	0.008	0.0010
10.14652211	0.043	0.042	0.0010
10.14760349	0.043	0.042	0.0010
10.14870094	0.043	0.042	0.0010
0.195652174	1	1	0.0000
1.435035389	1	1	0.0000
1.725639054	1	1	0.0000
1.817633292	1	1	0.0000
2.13744469	0.999	0.999	0.0000
2.226884345	0.999	0.999	0.0000
2.264757452	0.999	0.999	0.0000
2.310145535	0.999	0.999	0.0000
2.36505733	0.999	0.999	0.0000
2.409505908	0.999	0.999	0.0000
2.432748538	0.999	0.999	0.0000
2.472222222	0.999	0.999	0.0000
2.772693236	0.997	0.997	0.0000
2.775443511	0.997	0.997	0.0000
2.803938859	0.996	0.996	0.0000
2.81016731	0.996	0.996	0.0000
2.814135564	0.996	0.996	0.0000
2.819621248	0.996	0.996	0.0000
2.827557756	0.996	0.996	0.0000
2.898261278	0.995	0.995	0.0000
2.905438312	0.995	0.995	0.0000
2.909463487	0.995	0.995	0.0000
2.918332667	0.995	0.995	0.0000
2.984785375	0.994	0.994	0.0000
2.989712455	0.994	0.994	0.0000
3.071124855	0.992	0.992	0.0000
9.85129906	0.055	0.055	0.0000
9.852396514	0.055	0.055	0.0000
9.853477888	0.055	0.055	0.0000
9.858655479	0.054	0.054	0.0000
9.86013302	0.054	0.054	0.0000
9.862987988	0.054	0.054	0.0000
9.876017196	0.053	0.053	0.0000
9.881980226	0.053	0.053	0.0000
9.899843658	0.053	0.053	0.0000
9.916316527	0.053	0.053	0.0000
9.918963255	0.053	0.053	0.0000
9.921870231	0.053	0.053	0.0000
9.923370523	0.052	0.052	0.0000

9.923953273	0.052	0.052	0.0000
9.924527227	0.052	0.052	0.0000
10.14440621	0.043	0.043	0.0000
10.14545647	0.043	0.043	0.0000
10.14981484	0.042	0.042	0.0000
10.15094555	0.042	0.042	0.0000
10.15327696	0.042	0.042	0.0000
10.15625954	0.042	0.042	0.0000
10.17095266	0.041	0.041	0.0000
10.17656834	0.041	0.041	0.0000
10.18840066	0.041	0.041	0.0000
10.22305141	0.04	0.04	0.0000
10.22814018	0.039	0.039	0.0000
10.22988843	0.039	0.039	0.0000
10.23257212	0.039	0.039	0.0000
10.23529774	0.039	0.039	0.0000
10.23715786	0.039	0.039	0.0000
10.25105042	0.038	0.038	0.0000
10.28268904	0.037	0.037	0.0000
10.28470111	0.037	0.037	0.0000
10.28674203	0.037	0.037	0.0000
10.31873016	0.035	0.035	0.0000
10.495701	0.029	0.029	0.0000
10.50542175	0.029	0.029	0.0000
10.51097613	0.029	0.029	0.0000
10.5166122	0.029	0.029	0.0000
10.80145985	0.021	0.021	0.0000
10.80796392	0.021	0.021	0.0000
10.82063055	0.02	0.02	0.0000
10.82675698	0.02	0.02	0.0000
10.82732694	0.02	0.02	0.0000
10.83045977	0.02	0.02	0.0000
10.83624709	0.02	0.02	0.0000
10.83974849	0.02	0.02	0.0000
10.84324499	0.02	0.02	0.0000
10.96662959	0.016	0.016	0.0000
10.97167585	0.016	0.016	0.0000
10.98022912	0.016	0.016	0.0000
10.98829201	0.016	0.016	0.0000
11.3754287	0.01	0.01	0.0000
11.45642273	0.009	0.009	0.0000
11.47066775	0.009	0.009	0.0000
11.48148148	0.009	0.009	0.0000

11.48292824	0.009	0.009	0.0000
11.48686835	0.009	0.009	0.0000
11.49094951	0.009	0.009	0.0000
11.49430015	0.009	0.009	0.0000
11.51844532	0.008	0.008	0.0000
11.58910534	0.007	0.007	0.0000
11.59965035	0.007	0.007	0.0000
11.61188811	0.007	0.007	0.0000
11.62329514	0.007	0.007	0.0000
11.63591534	0.007	0.007	0.0000
11.64120802	0.007	0.007	0.0000
11.65383932	0.006	0.006	0.0000
11.660401	0.006	0.006	0.0000
11.67892157	0.006	0.006	0.0000
11.69924677	0.006	0.006	0.0000
11.73365854	0.006	0.006	0.0000
11.76888889	0.006	0.006	0.0000
11.86842569	0.005	0.005	0.0000
11.8999383	0.005	0.005	0.0000
11.91588235	0.005	0.005	0.0000
11.92296296	0.005	0.005	0.0000
11.93195521	0.005	0.005	0.0000
11.96785853	0.004	0.004	0.0000
11.99144621	0.004	0.004	0.0000
12.02832101	0.004	0.004	0.0000
12.04571692	0.004	0.004	0.0000
12.06117553	0.004	0.004	0.0000
12.08497246	0.004	0.004	0.0000
12.36623032	0.001	0.001	0.0000
12.43865718	0.001	0.001	0.0000
12.49031008	0.001	0.001	0.0000
12.5101626	0.001	0.001	0.0000
12.5306544	0.001	0.001	0.0000
12.63412817	0.001	0.001	0.0000
12.74423338	0.001	0.001	0.0000
12.88059701	0.001	0.001	0.0000
13.02	0.001	0.001	0.0000
13.57777778	0	0	0.0000
13.80819672	0	0	0.0000
15.01639344	0	0	0.0000
9.929823697	0.05	0.051	-0.0010
9.930312743	0.05	0.051	-0.0010
9.931031202	0.05	0.051	-0.0010

9.931739819	0.05	0.051	-0.0010
9.932653061	0.05	0.051	-0.0010
10.30189109	0.035	0.036	-0.0010
10.30418691	0.035	0.036	-0.0010
10.30651791	0.035	0.036	-0.0010
10.30888491	0.035	0.036	-0.0010
10.31251907	0.035	0.036	-0.0010
10.31621047	0.035	0.036	-0.0010
10.48695799	0.029	0.03	-0.0010
10.77739487	0.021	0.022	-0.0010
10.78069592	0.021	0.022	-0.0010
10.78432579	0.021	0.022	-0.0010
10.79225151	0.021	0.022	-0.0010
10.79855072	0.021	0.022	-0.0010
10.92051821	0.017	0.018	-0.0010
10.92647059	0.017	0.018	-0.0010
10.92940199	0.017	0.018	-0.0010
10.93274218	0.017	0.018	-0.0010
11.03439153	0.014	0.015	-0.0010
11.03935185	0.014	0.015	-0.0010
11.04230573	0.014	0.015	-0.0010
11.05177542	0.014	0.015	-0.0010
11.06465418	0.014	0.015	-0.0010
11.07601781	0.014	0.015	-0.0010
11.08430233	0.014	0.015	-0.0010
11.09410625	0.014	0.015	-0.0010
11.36730205	0.01	0.011	-0.0010
11.37250677	0.01	0.011	-0.0010
11.78319783	0.005	0.006	-0.0010
11.78983133	0.005	0.006	-0.0010
11.80793115	0.005	0.006	-0.0010
11.83677851	0.005	0.006	-0.0010
13.24932331	0	0.001	-0.0010
13.50710109	0	0.001	-0.0010
2.504416961	0.998	0.999	-0.0010
2.528001867	0.998	0.999	-0.0010
2.547470256	0.998	0.999	-0.0010
2.549395554	0.998	0.999	-0.0010
2.561742088	0.998	0.999	-0.0010
2.588913876	0.998	0.999	-0.0010
2.606253421	0.998	0.999	-0.0010
2.625410509	0.998	0.999	-0.0010
2.681233116	0.997	0.998	-0.0010

2.696178938	0.997	0.998	-0.0010
2.705518018	0.997	0.998	-0.0010
2.709005376	0.997	0.998	-0.0010
2.729340702	0.997	0.998	-0.0010
2.760640606	0.997	0.998	-0.0010
2.782694199	0.996	0.997	-0.0010
2.788655095	0.996	0.997	-0.0010
2.792699248	0.996	0.997	-0.0010
2.797026417	0.996	0.997	-0.0010
2.84178744	0.995	0.996	-0.0010
2.851113553	0.995	0.996	-0.0010
2.858425212	0.995	0.996	-0.0010
2.870200673	0.995	0.996	-0.0010
2.885136661	0.995	0.996	-0.0010
2.927292961	0.994	0.995	-0.0010
2.934466562	0.994	0.995	-0.0010
2.943802521	0.994	0.995	-0.0010
2.94757326	0.994	0.995	-0.0010
2.958229942	0.994	0.995	-0.0010
2.971966206	0.994	0.995	-0.0010
2.977891156	0.994	0.995	-0.0010
2.98204398	0.994	0.995	-0.0010
9.854543531	0.054	0.055	-0.0010
9.85610766	0.054	0.055	-0.0010
9.857649443	0.054	0.055	-0.0010
9.922778772	0.052	0.053	-0.0010
9.925092582	0.051	0.052	-0.0010
9.92564953	0.051	0.052	-0.0010
9.926466613	0.051	0.052	-0.0010
9.927532427	0.051	0.052	-0.0010
9.928314491	0.051	0.052	-0.0010
9.94130809	0.049	0.05	-0.0010
9.971428571	0.049	0.05	-0.0010
10.06920498	0.046	0.047	-0.0010
10.06968726	0.046	0.047	-0.0010
10.07168551	0.045	0.046	-0.0010
10.07220311	0.045	0.046	-0.0010
10.07272823	0.045	0.046	-0.0010
10.07409034	0.045	0.046	-0.0010
10.08163401	0.044	0.045	-0.0010
10.08230592	0.044	0.045	-0.0010
10.10461345	0.044	0.045	-0.0010
10.14286443	0.043	0.044	-0.0010

10.15810524	0.041	0.042	-0.0010
10.15936508	0.041	0.042	-0.0010
10.16130081	0.041	0.042	-0.0010
10.16394544	0.041	0.042	-0.0010
10.16739039	0.041	0.042	-0.0010
10.19672343	0.04	0.041	-0.0010
10.20142394	0.04	0.041	-0.0010
10.206188	0.04	0.041	-0.0010
10.21136194	0.04	0.041	-0.0010
10.21820779	0.04	0.041	-0.0010
10.22140523	0.04	0.041	-0.0010
10.24001536	0.038	0.039	-0.0010
10.24391856	0.038	0.039	-0.0010
10.24795082	0.038	0.039	-0.0010
10.2526327	0.037	0.038	-0.0010
10.25816123	0.037	0.038	-0.0010
10.27124375	0.037	0.038	-0.0010
10.27952494	0.037	0.038	-0.0010
10.28070521	0.037	0.038	-0.0010
10.28987029	0.036	0.037	-0.0010
10.29304422	0.036	0.037	-0.0010
10.29631255	0.036	0.037	-0.0010
10.32666667	0.034	0.035	-0.0010
10.33558559	0.034	0.035	-0.0010
10.34497526	0.034	0.035	-0.0010
10.35591245	0.034	0.035	-0.0010
10.36101554	0.034	0.035	-0.0010
10.36498295	0.034	0.035	-0.0010
10.36900871	0.034	0.035	-0.0010
10.37175235	0.034	0.035	-0.0010
10.3759611	0.033	0.034	-0.0010
10.38023363	0.033	0.034	-0.0010
10.38314739	0.033	0.034	-0.0010
10.38610614	0.033	0.034	-0.0010
10.38911095	0.033	0.034	-0.0010
10.39216289	0.033	0.034	-0.0010
10.45628036	0.031	0.032	-0.0010
10.46955381	0.03	0.031	-0.0010
10.47270696	0.03	0.031	-0.0010
10.47621251	0.03	0.031	-0.0010
10.48110534	0.03	0.031	-0.0010
10.52241715	0.028	0.029	-0.0010
10.52830941	0.028	0.029	-0.0010

10.53438228	0.028	0.029	-0.0010
10.5405486	0.028	0.029	-0.0010
10.54342667	0.028	0.029	-0.0010
10.54608145	0.028	0.029	-0.0010
10.7422712	0.022	0.023	-0.0010
10.74861316	0.022	0.023	-0.0010
10.75550021	0.022	0.023	-0.0010
10.75809822	0.022	0.023	-0.0010
10.76098973	0.022	0.023	-0.0010
10.76629477	0.022	0.023	-0.0010
10.81391147	0.02	0.021	-0.0010
10.84943351	0.019	0.02	-0.0010
10.85398817	0.019	0.02	-0.0010
10.85620301	0.019	0.02	-0.0010
10.859035	0.019	0.02	-0.0010
10.86211825	0.019	0.02	-0.0010
10.86961088	0.019	0.02	-0.0010
10.89159892	0.018	0.019	-0.0010
10.89828229	0.018	0.019	-0.0010
10.90567327	0.018	0.019	-0.0010
10.95619048	0.016	0.017	-0.0010
10.96148148	0.016	0.017	-0.0010
10.9642401	0.016	0.017	-0.0010
10.99205097	0.015	0.016	-0.0010
10.99618321	0.015	0.016	-0.0010
11.01282051	0.015	0.016	-0.0010
11.0275264	0.015	0.016	-0.0010
11.17878648	0.013	0.014	-0.0010
11.27588176	0.011	0.012	-0.0010
11.28018976	0.011	0.012	-0.0010
11.29074685	0.011	0.012	-0.0010
11.30270112	0.011	0.012	-0.0010
11.31276088	0.011	0.012	-0.0010
11.33748616	0.011	0.012	-0.0010
11.35857143	0.011	0.012	-0.0010
9.928824721	0.05	0.052	-0.0020
9.929327739	0.05	0.052	-0.0020
9.934199134	0.049	0.051	-0.0020
9.936085927	0.049	0.051	-0.0020
9.938432977	0.049	0.051	-0.0020
10.29962967	0.035	0.037	-0.0020
10.48331479	0.029	0.031	-0.0020
10.48499104	0.029	0.031	-0.0020

10.77193933	0.021	0.023	-0.0020
10.91229193	0.017	0.019	-0.0020
10.91576175	0.017	0.019	-0.0020
10.9163486	0.017	0.019	-0.0020
10.94006684	0.016	0.018	-0.0020
10.94863142	0.016	0.018	-0.0020
11.0305789	0.014	0.016	-0.0020
11.10702614	0.013	0.015	-0.0020
11.11499611	0.013	0.015	-0.0020
11.11944056	0.013	0.015	-0.0020
11.12338028	0.013	0.015	-0.0020
11.1315621	0.013	0.015	-0.0020
11.15641711	0.013	0.015	-0.0020
11.18756611	0.012	0.014	-0.0020
11.19701493	0.012	0.014	-0.0020
11.20150376	0.012	0.014	-0.0020
11.20756437	0.012	0.014	-0.0020
11.21320346	0.012	0.014	-0.0020
11.21782988	0.012	0.014	-0.0020
11.22607164	0.012	0.014	-0.0020
11.23553965	0.012	0.014	-0.0020
11.24515504	0.012	0.014	-0.0020
11.25880282	0.011	0.013	-0.0020
11.27138671	0.011	0.013	-0.0020
11.36181818	0.01	0.012	-0.0020
2.64963991	0.997	0.999	-0.0020
2.66420636	0.997	0.999	-0.0020
10.03267974	0.046	0.048	-0.0020
10.06601307	0.046	0.048	-0.0020
10.06734694	0.046	0.048	-0.0020
10.06826018	0.046	0.048	-0.0020
10.06872933	0.046	0.048	-0.0020
10.0701763	0.045	0.047	-0.0020
10.07092555	0.045	0.047	-0.0020
10.07605552	0.044	0.046	-0.0020
10.07783162	0.044	0.046	-0.0020
10.08002049	0.044	0.046	-0.0020
10.12780727	0.043	0.045	-0.0020
10.12987561	0.043	0.045	-0.0020
10.13528955	0.043	0.045	-0.0020
10.14085206	0.043	0.045	-0.0020
10.39819268	0.032	0.034	-0.0020
10.40295519	0.032	0.034	-0.0020

10.40850945	0.032	0.034	-0.0020
10.41816416	0.032	0.034	-0.0020
10.42403356	0.032	0.034	-0.0020
10.43961353	0.031	0.033	-0.0020
10.44778613	0.031	0.033	-0.0020
10.45283664	0.031	0.033	-0.0020
10.45927079	0.03	0.032	-0.0020
10.46103239	0.03	0.032	-0.0020
10.46332737	0.03	0.032	-0.0020
10.46589147	0.03	0.032	-0.0020
10.55175038	0.027	0.029	-0.0020
10.55777778	0.027	0.029	-0.0020
10.56368794	0.027	0.029	-0.0020
10.56940223	0.027	0.029	-0.0020
10.57556936	0.027	0.029	-0.0020
10.71175279	0.023	0.025	-0.0020
10.71685509	0.023	0.025	-0.0020
10.8779562	0.018	0.02	-0.0020
10.88354839	0.018	0.02	-0.0020
10.88799283	0.018	0.02	-0.0020
10.62007934	0.025	0.028	-0.0030
10.62284483	0.025	0.028	-0.0030
10.62718531	0.025	0.028	-0.0030
10.62964594	0.025	0.028	-0.0030
10.64104759	0.025	0.028	-0.0030
10.65288434	0.025	0.028	-0.0030
10.65767974	0.025	0.028	-0.0030
10.68104515	0.024	0.027	-0.0030
10.68337484	0.024	0.027	-0.0030
10.69130296	0.024	0.027	-0.0030
10.69848756	0.023	0.026	-0.0030
10.70176303	0.023	0.026	-0.0030
10.70644338	0.023	0.026	-0.0030
10.70894064	0.023	0.026	-0.0030
10.42705167	0.031	0.034	-0.0030
10.43167702	0.031	0.034	-0.0030
10.59060695	0.026	0.029	-0.0030
10.6027653	0.026	0.029	-0.0030
10.60504373	0.026	0.029	-0.0030
10.60606061	0.026	0.029	-0.0030
10.60837381	0.026	0.029	-0.0030
10.61303582	0.026	0.029	-0.0030
10.72467573	0.022	0.025	-0.0030

10.73261056	0.022	0.025	-0.0030
10.73677458	0.022	0.025	-0.0030
10.73949789	0.022	0.025	-0.0030
10.61742682	0.025	0.029	-0.0040
10.66421569	0.024	0.028	-0.0040
10.6716792	0.024	0.028	-0.0040
10.67848192	0.024	0.028	-0.0040

Table S2 | Univariate cox regression analyses for in-hospital mortality in septic patients with atrial fibrillation.

Variables	HR (95% CI)	*p*-value
Age	1.02 (1.01~1.02)	<0.001
Gender	1.20 (1.08~1.34)	0.001
Ethnicity		
White	1.21 (0.85~1.74)	0.289
Black	1.24 (0.82~1.86)	0.306
Other	1.84 (1.27~2.67)	0.001
Myocardial infarct	1.22 (1.09~1.38)	0.001
Congestive heart failure	1.26 (1.14~1.40)	<0.001
Peripheral vascular disease	1.13 (0.99~1.30)	0.078
Cerebrovascular disease	1.38 (1.20~1.58)	<0.001
Dementia	1.18 (0.95~1.47)	0.14
Chronic pulmonary disease	1.09 (0.97~1.22)	0.134
Rheumatic disease	1.06 (0.82~1.38)	0.638
Peptic ulcer disease	1.59 (1.21~2.10)	0.001
Paraplegia	1.48 (1.17~1.85)	0.001
Renal disease	1.47 (1.32~1.64)	<0.001
Malignant cancer	1.90 (1.65~2.19)	<0.001
Metastatic solid tumor	2.67 (2.18~3.29)	<0.001
Diabetes	0.97 (0.85~1.12)	0.705
Hypertension	11.81 (9.77~14.27)	<0.001
Hemoglobin	1.00 (0.98~1.03)	0.759
RDW	1.13 (1.11~1.15)	<0.001
HRR	0.92 (0.89~0.95)	<0.001
Hematocrit	0.99 (0.98~1.00)	0.159
Platelets	1.00 (1.00~1.00)	<0.001
WBC	1.00 (1.00~1.01)	0.344
Albumin	0.91 (0.8~1.03)	0.146
Anion gap	1.07 (1.06~1.08)	<0.001
Bicarbonate	1.00(0.99~1.01)	0.536
Bun	1.01 (1.01~1.01)	<0.001
Calcium	1.22 (1.14~1.29)	<0.001
Chloride	0.97 (0.96~0.98)	<0.001

Creatinine	1.14 (1.11~1.17)	<0.001
Sodium	1.01 (1.00~1.02)	0.013
Lymphocytes	1.00 (1.00~1.00)	0.092
Monocytes	1.00 (1.00~1.00)	0.6
Neutrophils	1.00 (1.00~1.00)	0.69
INR	1.09 (1.06~1.12)	<0.001
PT	1.01 (1.01~1.01)	<0.001
APTT	1.00 (1.00~1.00)	<0.001
ALT	1.00 (1.00~1.00)	0.356
ALP	1.00 (1.00~1.00)	0.399
AST	1.00 (1.00~1.00)	0.4
CKCPK	1.00 (1.00~1.00)	0.16
CKMB	1.00 (1.00~1.00)	0.837
HR	1.01 (1.01~1.01)	<0.001
SBP	1.03 (1.02~1.03)	<0.001
DBP	1.03 (1.03~1.04)	<0.001
MBP	1.04 (1.03~1.05)	<0.001
RR	1.08 (1.06~1.09)	<0.001
Temperature	1.07 (0.97~1.19)	0.163
Spo2	0.94 (0.92~0.96)	<0.001
Glucose	1.00 (1.00~1.00)	0.552
Warfarin	0.52 (0.41~0.66)	<0.001
Amiodarone	0.94 (0.82~1.07)	0.346
Dopamine	1.47 (1.19~1.81)	<0.001
Epinephrine	0.90 (0.75~1.08)	0.255
Vasopressin	1.88 (1.64~2.16)	<0.001
Antibiotic	0.83 (0.72~0.95)	0.007
Ventilation	1.08 (0.97~1.20)	0.14
APSIII	1.02 (1.02~1.02)	<0.001
SOFA	1.03 (1.00~1.06)	0.022
GCS	0.92 (0.91~0.93)	<0.001
OASIS	1.04 (1.03~1.04)	<0.001
Length of ICU stay	1.04 (1.03~1.05)	<0.001
Length of hospital stay	1.00 (1.00~1.00)	0.302

Abbreviations: HR, heart rate; SBP, systolic blood pressure; DBP, diastolic blood pressure; MBP, mean blood pressure; RR, respiratory rate; SpO2, percutaneous oxygen saturation; RDW, red cell distribution width; HRR, hemoglobin/red cell distribution width; Bun, blood urea nitrogen; INR, international normalized ratio; PT, prothrombin time; APTT, activated partial thromboplastin time; ALT, alanine aminotransferase; AST, aspartate aminotransferase; CK-MB, creatine kinase-MB isoenzyme; APS III, acute physiology score III; SOFA, sequential organ failure assessment score; GCS, Glasgow coma score; OASIS, oxford acute severity of illness score; ICU, Intensive care unit.
